# USP20, a Super-enhancer Regulated Gene, Promotes Acute Myeloid Leukemia Progression through CTNNB1 Deubiquitination

**DOI:** 10.7150/ijbs.122898

**Published:** 2026-02-11

**Authors:** Jia Cheng, Fang Fang, Zhiheng Li, Jianwei Wang, Linbo Cai, Ling Xu, Yanfang Tao, Juanjuan Yu, Gen Li, Zimu Zhang, Zexi Cui, Yang Yang, Tiandan Li, Di Wu, Xiaolu Li, Yifang Ding, Zong Zhai, Mengmeng Gu, Xue Li, Xingxing Wu, Pengju Yang, Chunxia Shi, Huike Bai, Xiaodong Wang, Lanlan Han, Lei Shi, Jianqin Li, Jian Pan

**Affiliations:** 1 Children's Hospital of Soochow University, Suzhou 215003, China.; 2 Institute of Pediatric Research, Children's Hospital of Soochow University, Suzhou, Jiangsu 215003, China.; 3 Department of Traditional Chinese Medicine, Children's Hospital of Soochow University, Suzhou, Jiangsu 215003, China.; 4 Department of Hematology, Children's Hospital of Soochow University, Suzhou, Jiangsu 215003, China.; 5 Department of Orthopaedics, Children's Hospital of Soochow University, Suzhou, Jiangsu 215003, China.; 6 Department of Pediatrics, The Affiliated Zhangjiagang Hospital of Soochow University, Suzhou 215600, China.; 7 Department of Medicinal Chemistry, Jiangsu Key Laboratory of Drug Design and Optimization, China Pharmaceutical University, Nanjing 210009, China; 8 Department of Pediatrics, The Affiliated Hospital of Xuzhou Medical University, Xuzhou 221000, China.

**Keywords:** USP20, super-enhancer, AML, CTNNB1, AS1517499

## Abstract

Acute Myeloid Leukemia (AML) is a heterogeneous hematologic malignancy driven by genetic and epigenetic alterations, where super-enhancers (SEs) play key oncogenic roles, representing promising therapeutic targets in AML. Through H3K27ac ChIP-seq profiling of 7 AML cell lines and 13 primary samples, we identified USP20 as the deubiquitinase that most frequently associated with super-enhancers. Public database analysis confirmed USP20 overexpression in AML and its correlation with adverse prognosis. Genetic knockdown of USP20 via shRNA significantly induced apoptosis and suppressed proliferation in AML cells *in vitro*, while *in vivo* depletion of USP20 attenuated leukemia development and improved overall survival. AS1517499, a novel USP20 inhibitor identified via virtual screening, recapitulated these anti-leukemic effects *in vitro* and *in vivo* with low toxicity. Mechanistically, USP20 interacts with CTNNB1 and stabilizes the CTNNB1 protein via deubiquitination. Cut&tag analysis indicated that USP20 co-localizes with CTNNB1 on the genome, where they jointly regulate target genes in AML. Collectively, our study identified USP20 as a super-enhancer-regulated oncogene maintaining AML cell survival and proliferation through CTNNB1 stabilization. Pharmacologic targeting of USP20 with AS1517499 presents a promising therapeutic strategy targeting the SE-USP20- CTNNB1 axis.

## Introduction

Acute Myeloid Leukemia (AML) is an aggressive hematologic malignancy characterized by uncontrolled proliferation and abnormal differentiation of hematopoietic cells, resulting in blasts infiltrating the bone marrow, blood, and other tissues^1^. AML exhibits significant heterogeneity in its presentation and pathogenesis, involving various genetic and epigenetic abnormalities. These include recurrent gene mutations in FLT3, IDH1/2, NPM1; genes involving RNA methylation modifications (METTL13-mediated m6A); and dysregulation of signaling pathways aberrant MYC expression^2^. Current treatment modalities for AML encompass conventional chemotherapy, targeted therapy, immunotherapy, and stem cell transplantation^3^. Although these approaches have improved outcomes for specific subsets of AML, the overall prognosis remains poor—reflected in a 5-year survival rate of only 29 percent^4^. Despite notable advancements in new therapeutic strategies and combinations, challenges persist due to the high heterogeneity of AML cases. Consequently, these unmet clinical needs highlight the imperative to identify novel therapeutic vulnerabilities and develop precision strategies to improve patient survival.

Super-enhancers (SEs) formed through the clustering of multiple typical enhancers along with their associated transcriptional cofactors, creating regions with exceptionally high densities of transcription factors like OCT4 and SOX2, cofactors (including the Mediator complex), and epigenetic markers such as H3K27ac that drive the expression of key genes essential for cellular identity^5^. The aberrant expression of genes induced by super-enhancers is implicated in various biological processes, including their role in the development and differentiation of embryonic stem cells through the activation of core genes that determine cell fate^6^. In acute myeloid leukemia, these regulatory elements are frequently hijacked to drive aberrant oncogenic transcription programs that maintain malignant characteristics^7^. The BRD4 inhibitor JQ1 has demonstrated therapeutic potential by obstructing BRD4's binding to acetylated histones, thereby disrupting the SE complex^8^. Recent studies have revealed critical roles for super-enhancer (SE)-mediated oncogenic programs in acute myeloid leukemia (AML) pathogenesis. Notably, we discovered a specific super-enhancer requiring BRG1 maintenance at the transcriptional start site of the proto-oncogene MYC, which is critical for MYC expression in AML and facilitates leukemia progression^9^. And studies have identified that the inv(3)/t(3; Chromosomal rearrangement of the 3) fragment generates a super enhancer for the oncogenic driver EVI1, simultaneously dysregulates both the GATA2 transcription factor and the EVI1 oncogene, which subsequently promotes the development of leukemia^10^. Further investigations in AML have identified the SE associated gene CAPG which can activate the NF-κB pathway, thereby influencing AML progression. Therefore, investigating super enhancers that drive AML onset and exploring novel drugs targeting super enhancers represent promising strategies for AML treatment.

Deubiquitinating enzymes (DUBs), specifically isopeptidases that cleave ubiquitin's carboxyl terminus, comprise five major classes, with the ubiquitin-specific protease (USP) family representing the largest and most functionally diverse subgroup^11^. Numerous USP family members have been implicated in in cancer through diverse mechanisms. For instance, USP8 has been shown to promotes chemotherapy resistance in aggressive breast cancer by directly deubiquitinating and stabilizing the type II TGF-β receptor (TβRII) on cell membranes and extracellular vesicles (TEVs) of tumor cells, thereby enhancing TβRII+ circulating extracellular vesicles (crEVs)-mediated T cell depletion^12^. USP7 (Ubiquitin-specific protease 7) directly interacts with NOTCH1 at super-enhancer sites in T-cell acute lymphoblastic leukemia (T-ALL), promoting leukemic cell proliferation both *in vitro* and *in vivo*^13^. These findings highlight the multifaceted roles of USPs in cancer development and their potential as therapeutic targets.

Ubiquitin-specific protease 20 (USP20), a member of the USP subfamily of deubiquitinating enzymes (DUBs), is encoded by the *USP20* gene located at the q34.11 locus on chromosome 9 and regulated by von Hippel-Lindau protein (pVHL)^14^. Emerging evidence implicates that USP20 plays significant physiological roles in antiviral immunity, cancer, metabolic disorders, and neurological conditions^15^. Notably, USP20 enriched in cardiomyocytes can regulate K63 ubiquitination of STAT3, forming a cardiomyocyte-specific USP20-STAT3-CARM1 axis that exerts protective effects against myocardial hypertrophy^16^. Analysis of USP20 expression across various cancers has revealed significant differences between tumor tissues and normal tissues, suggesting that USP20 may serve as a prognostic biomarker and tumor immunotherapy^17^. However, the specific mechanisms by which USP20 functions as a super-enhancer-associated regulator in acute myeloid leukemia (AML) remain poorly understood. This study systematically investigates USP20's leukemogenic potential in AML, with particular focus on its super-enhancer-mediated regulatory networks and their therapeutic implications.

## Methods

### Cell lines

The cell lines CMK, kasumi-1, NB4, MV4-11, THP-1, MOLM13, HL60, and HEK293FT were obtained from the National Collection of Authenticated Cell Cultures in Shanghai, China. All cells mentioned above have been sourced from the National Collection of Authenticated Cell Cultures in Shanghai, China, with authenticated STR profiling and tested negative for mycoplasma contamination. All acute myeloid leukemia (AML) cells were cultured in RPMI 1640 medium (VivaCell, China), supplemented with 10% fetal bovine serum (FBS; Sigma-Aldrich, Germany) and 1% penicillin-streptomycin (Beyotime, China). HEK293FT cells were maintained in DMEM medium (VivaCell, China), also supplemented with 10% FBS (Sigma-Aldrich, Germany) and 1% penicillin-streptomycin (Beyotime, China).

### ChIP-Seq (Chromatin immunoprecipitation followed by sequencing) analysis

In the present study, raw data of ChIP-Seq H3K27ac datasets were aligned to UCSC hg38 (the reference genome) with Bowtie2 (v 2.4.1)^18^. Peaks were called with MACS2 (v 3.0.2)^19^. The bigwig files for these datasets were subsequently visualized using Integrative Genomics Viewer (IGV)^20^. Then, we identified super enhancers (SEs) by the ROSE (Rank Order of Super Enhancers) method^21^.

### Dual-luciferase reporter assay

The USP20 super enhancers were subdivided into three fragments: E1, E2, and E3. Each enhancer plasmid fragment, along with 2 μg of the Relina internal reference, was transfected into kasumi-1 MV4-11 and CMK cells using M5 Hiper Lipo2000 Transfection Reagent (mei5bio, China). After a 48-hour incubation period, luciferase activity was measured utilizing the Dual-Luciferase Reporter Assay System (Promega, USA). The results were normalized by calculating the ratio of firefly luciferase activity to Renilla luciferase activity. The sequences of the enhancers are provided in Supplementary Table 1.

### Apoptosis and cell cycle assay

To assess apoptosis, we collected 1-5 × 10^5 cells, washed them with PBS (1×) (Servicebio, China), and utilized the Annexin V-FITC/PI Apoptosis Detection Kit (Vazyme, China) for detection on a Beckman Gallios™ Flow Cytometer (Beckman, Germany). For the cell cycle analysis, we collected 5-10 × 10^5 cells, washed them with PBS, and treated them overnight in 70% ethanol at 4 °C. The Cell Cycle and Apoptosis Analysis Kit (PI staining) (MCE, USA) were employed for detection on the same Beckman Gallios™ Flow Cytometer. The resulting data were analyzed and processed using FlowJo software.

### Cell proliferation and cytotoxicity

Virus-infected cells were screened using puromycin, with 2-3 × 10^3 cells seeded per well in 96-well plates containing 100 μL of RPMI 1640 medium. On days 1, 3, 5, and 7, the cell counting kit (APExBIO, USA) was added to each well. The plates were then incubated in a cell incubator for three hours before measuring the absorbance at 450 nm using a spectrometer (Thermo, USA). Subsequently, after adding 1-2 × 10^4 cells per well along with a specific concentration of inhibitor to each well in the same 96-well plate, the plates were incubated at 37 °C with an atmosphere of 5% CO2 for durations of either 24, 48, or 72 hours. Following this incubation period and subsequent addition of the cell counting kit (APExBIO, USA), absorbance measurements at a wavelength of 450 nm were again taken using a spectrometer (Thermo, USA).

### Western blotting

Cells were collected and washed with PBS (1×) (Servicebio, China), followed by the addition of RIPA lysis buffer (Beyotime, China) to lyse the cells. The samples were then loaded onto SDS-PAGE gels (gelGenScript, China) at a protein concentration of 20-30 μg per well for electrophoresis. Transfer was conducted using PVDF membranes (Merck Millipore, USA). After blocking at room temperature with TBST containing 5% skimmed milk for one hour, the primary antibody was incubated overnight at 4 °C. Finally, chemiluminescence detection was performed using the AI600 image gel imaging analyzer (GE, USA). Details regarding the primary antibody can be found in Supplementary Table 2.

### Co-immunoprecipitation

The co-immunoprecipitation (Co-IP) experiment was performed using the Protein A/G Magnetic IP/Co-IP Kit (Vazyme, China). Specifically, 1 × 10^6 cells were incubated with USP20 antibody at room temperature for 30 minutes, followed by incubation with NP-40 washed beads at room temperature for an additional 30 minutes. Finally, denaturing elution was carried out using SDS-PAGE Sample Loading buffer, and the resulting supernatant was utilized for western blot detection.

### *In vivo* experiments

Four to six-week-old NSG mice (SHANGHAI MODEL ORGANISMS, China) were randomly assigned to two groups. One group received an injection of 1 × 10^6 firefly luciferase-labeled kasumi-1 cells transfected with sh-NC, while the other group was injected with sh-USP20 via the tail vein. On days 15, 20, and 25 post-injection, D-luciferin sodium salt (GoldBio, USA) was administered intraperitoneally into the mice. *In vivo* imaging was conducted using a Berthold imager (Berthold, Germany) under anesthesia approximately 5-15 minutes after D-luciferin administration on day 30. Following euthanasia through CO2 inhalation anesthesia, various tissues including liver, spleen, and bone were harvested for subsequent analyses such as hematoxylin-eosin staining, detection of CD45-positive cells, and immunohistochemical analysis. In another *in vivo* experiment involving AS1517499 (TargetMol, China), female NSG mice were also randomly divided into two groups; each group received an injection of 1 × 10^6 MV4-11 cells expressing firefly luciferase via the tail vein. Five days later, the experimental group underwent intraperitoneal injection of AS1517499 at a dosage of 5 mg/kg. The control group received an equivalent volume of normal saline. *In vivo* imaging was performed every seven days using a Berthold imager (Berthold, Germany), followed by tissue collection from liver, spleen and bone for further experimentation.

### Clone formation assay

The sub gel was prepared using 1.2% agarose gel (SIGMA, USA) supplemented with 20% FBS, 2× RPMI1640 (Pricella, China), and bispecific antibodies. Following coagulation, cells transfected with sh-NC and sh-USP20 were mixed with 0.7% agarose gel. The mixture was cultured in a cell incubator at 37 °C with 5% CO2, and the medium was changed every two days. After three weeks, clone formation staining was conducted using Wright-Giemsa Staining Solution (Beyotime, China), and the number of clones formed was counted.

### RNAseq analysis

RNA-Seq analysis was detected by Novogene Bioinformatics Technology Co., Ltd. (Beijing, China). Differentially expressed genes (DEGs) were identified using DESeq2 analysis (|log2FoldChange| > 1.0 and p < 0.05). Enrichment analysis was conducted using GSEA software, which was jointly developed by UC San Diego and the Broad Institute, to obtain a deeper understanding of the biological processes linked to the differentially expressed genes (DEGs).

### Quantitative real-time PCR

RNA extraction was performed using the Fast Pure Cell/Tissue Total RNA Isolation Kit V2 (Vazyme, China). The extracted RNA was subsequently reverse transcribed into complementary DNA (cDNA) utilizing the Hifair® III 1st Strand cDNA Synthesis Kit (YEASEN, China). Quantitative real-time PCR (qRT-PCR) analysis was conducted with the Hifair® III One Step RT-qPCR SYBR Green Kit (YEASEN, China) on the LightCycler 480 real-time fluorescence quantification system (Roche, Germany). All gene expressions were calculated based on the average of three biological replicates. The primers used in this study are detailed in Supplementary Table 3.

### Lentivirus packaging and transfection

The shRNA targeting USP20 was synthesized by Aiki Biotechnology Co., Ltd. (China) and subsequently cloned into the pLKO.1-puro vector. The pre-mixed packaged plasmids, prepared using PEI (Sigma-Aldrich, USA), consisted of 8 μg psPAX2, 6 μg pMD2.G (Cambridge, USA), and 2 μg of the USP20 knockdown plasmid. These components were introduced into HEK293FT cells at a density of 70-80%. Following incubation in a cell incubator at 37 °C with 5% CO2 for 48 hours, a concentrated solution of PEG8000 was utilized as a pre-packaged lentivirus reserve. For transfection with the lentivirus, it was mixed with AML cells that had been resuspended in RPMI-1640 medium containing 10 μg/mL polybrene (Sigma-Aldrich) and then placed in 12-well plates. After an incubation period of 12 hours, the medium was replaced with fresh RPMI-1640 medium. Selection began after an additional period of 48 hours post-transfection using puromycin at a concentration of 1 µg/mL (Beyotime, China). The sequences for shRNA are provided in Supplementary Table 4.

### USP20 inhibition assay

Test compounds were prepared as 10 mM stocks in 100% DMSO and serially diluted into the assay plate using an Echo liquid handler, achieving a final reaction concentration of 1% DMSO. Enzyme and substrate solutions were prepared in a modified Tris assay buffer, with the latter containing Ubiquitin Rhodamine 110 Protein (Ub-Rho). Then, 10 µL of the enzyme solution was aliquoted into the assay plate, with 1x assay buffer used in place of enzyme for the low-control wells. After a 1-hour incubation at room temperature, the reaction was initiated by adding 10 µL of the substrate solution. The plate was briefly centrifuged and shaken for 30 seconds each to mix. Reaction kinetics were monitored for 30 minutes on a SpectraMax Paradigm plate reader (excitation/emission: 480/540 nm), with data collected directly by the instrument's software.

### Molecular dynamics (MD) simulations of GSK2643943A and AS1517499 with USP20

To further elucidate the binding stability and dynamic interaction characteristics of AS1517499 with USP20, we performed 100-nanosecond Molecular Dynamics (MD) simulations using Maestro (Schrodinger LLC., New York City; 2021) with GSK2643943A as a reference ligand. All simulations employed the OPLS4 force field and the SPC water model. The system was solvated using the System Builder module with SPC water molecules supplemented with 0.15 M NaCl. Na/Cl ions were added to maintain charge neutrality. The system was loaded into the Molecular Dynamics module, and 100 ns all-atom simulations were executed under the NPT ensemble using the OPLS4 force field. To eliminate boundary effects, the simulations were conducted under periodic boundary conditions. The system temperature and pressure were maintained at 300 K and 1.01325 bar, respectively, with trajectory files and energy data collected at 10 ps intervals, yielding 10,000 frames. Van der Waals and short-range electrostatic interactions were truncated at 9 Å, while long-range electrostatics were calculated using the Particle Mesh Ewald method. Hydrogen bond constraints were applied via the Matrix-inversion SHAKE algorithm, and integration was advanced with a 2 fs time step using the Reversible Reference System Propagator Algorithm, updating long-range electrostatics every 6 fs. System stability and interaction behaviors were assessed using multiple metrics, including Root Mean Square Deviation (RMSD) for overall conformational stability, Root Mean Square Fluctuation (RMSF) for residue-level flexibility, and Radius of Gyration (Rg) for protein compactness. Hydrogen bond analysis monitored the number and persistence of intermolecular hydrogen bonds during the simulation, and free energy landscapes were examined to confirm thermodynamic equilibrium.

### CRISPR-mediated silencing of USP20

CRISPR-Mediated Silencing of USP20 Initially, we established the CRISPR/Cas9 system by transferring lentiviruses containing Cas9 into MV4-11 and CMK cells, utilizing a screening concentration of 6 µg/ml Blasticidin S. Subsequently, the sgRNA targeting USP20 was cloned into the lenti-crispr plasmid synthesized by IGE Biotechnology LTD (Guangzhou, China). Finally, both the sg-RNA-USP20 and control sequences were introduced into cells stably expressing Cas9 through lentivirus packaging and transfection. The sequence of the sgRNA is provided in Supplementary Table 5.

### Cut&tag (cleavage under targets and tagmentation)

The raw data from the Cut&tag experiments were aligned to the UCSC hg38 reference genome using Bowtie2 (v 2.4.1)^18^. Peaks representing regions of interest were identified using MACS2 (v3.0.2)^19^. Detailed antibody information can be found in Supplementary Table 2.

### Statistics and analysis

All experiments were conducted independently, with a minimum of three biological replicates for each condition. Statistical analyses were primarily performed using t-tests and one-way ANOVA, utilizing GraphPad Prism 9.5.0 software for data evaluation. RNA-seq differential expression analysis was performed with False Discovery Rate (FDR) correction. Significance levels are indicated as follows: no significant difference was observed (ns), * P < 0.05, ** P < 0.01, *** P < 0.001, **** P < 0.0001. All error bars represent standard deviations, and n ≥ 3.

## Results

### USP20 is an SE-regulated gene and correlates with poor prognosis in AML

**Figure 1:**
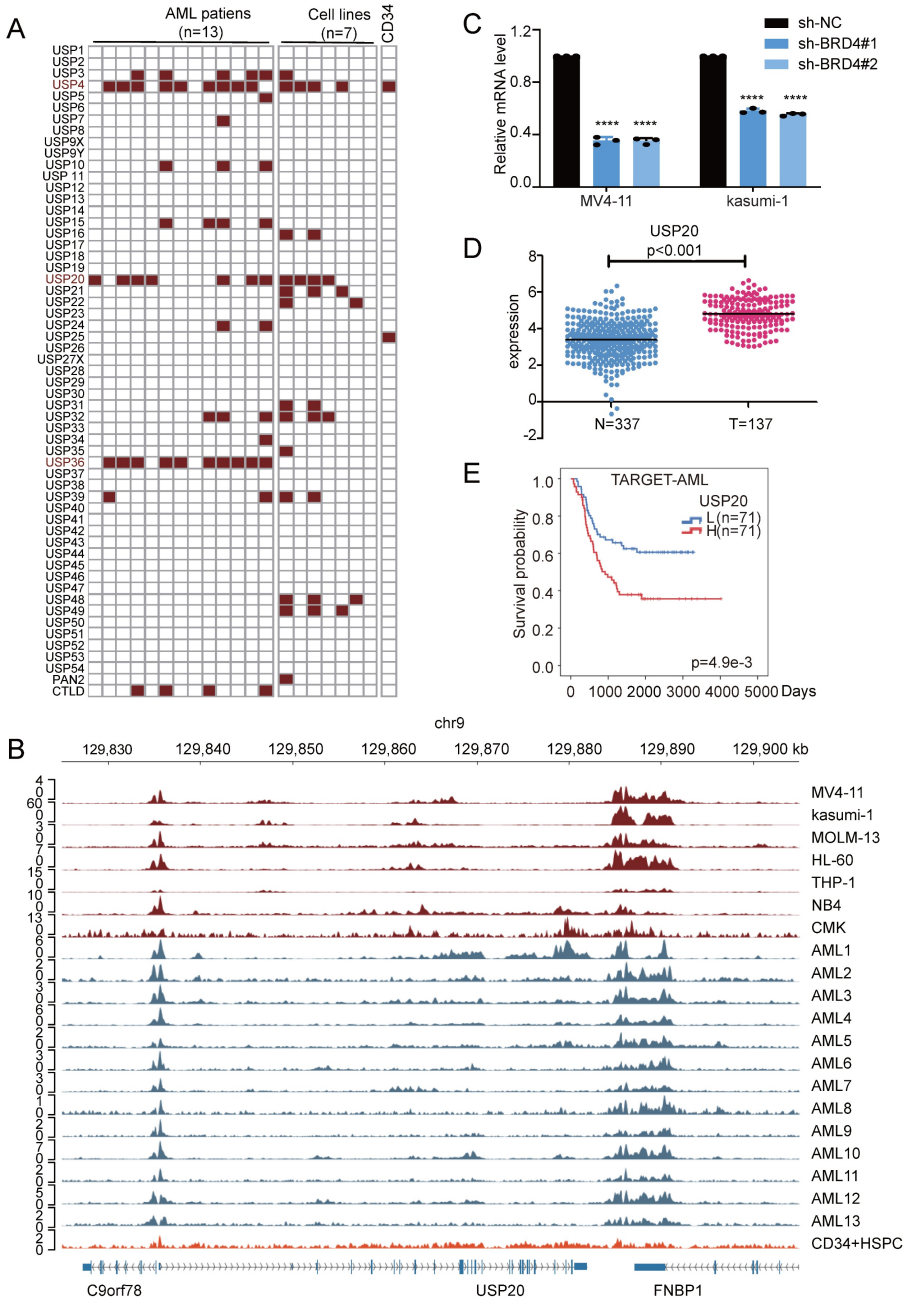
Identification of USP20 as a super-enhancer regulatory gene in acute myeloid leukemia (AML) and its high expression correlating with poor prognosis in AML. (A) Screening for potential super enhancers within the USP family was conducted by analyzing H3K27ac ChIP-seq data from seven AML cell lines and thirteen AML patient samples. (B) By utilizing the IGV visualization tool to compare the H3K27ac ChIP-seq results from our seven AML cell lines and thirteen patients, we identified an enrichment region near the USP20 gene, suggesting a strong likelihood of a super enhancer's presence. Notably, no such enrichment near USP20 was observed in H3K27ac ChIP-seq data derived from CD34-positive hematopoietic stem cells (HSCs) available in the GEO database. (C) The knockdown of BRD4 in AML cells resulted in a significant decrease in USP20 mRNA expression levels. Data obtained from the GEO dataset GSE26713 indicated that T-ALL patients exhibited significantly higher mRNA expression levels of USP20 compared to bone marrow samples collected from healthy donors. (D) According to public databases, mRNA expression levels of USP20 were markedly elevated in AML samples when compared to those found in healthy bone marrow specimens. (E) Analysis using data from the TARGET database revealed that AML patients with elevated levels of USP20 expression had poorer survival rates. Data are presented as mean ± standard deviation (mean ± SD); no significant difference was observed (ns), * P < 0.05, ** P < 0.01, *** P < 0.001, **** P < 0.0001. All error bars in the text represent standard deviations.

To systematically identify the ubiquitin-specific protease (USP) family genes which potentially regulated by SEs in AML, we analyzed H3K27ac ChIP-seq data from 13 AML^22^ patient samples (GSE188605, GSE299363) and 7 AML cell lines, including MV4-11 (GSE80779)^23^, kasumi-1 (GSE242532)^24^, MOLM-13 (GSE80779)^25^, HL-60 (GSE106359)^26^, THP-1 (GSE123872)^27^, NB4 (GSE188750)^28^ and CMK cells (GSE294132, Supplementary Table 6-7) and CD34+ (GSE131459)^29^. Following the acquisition of additional CHIP-seq data from 66 AML patients and four HSPC+ samples based on research by Ravindra Majeti et al.^30^, we employed ROSE analysis to generate corresponding super-enhancer (SE) distribution maps. Results showed that among USP family members, gene loci for USP4, USP15, USP36, USP48, and USP20 overlapped with SE regions across multiple samples. (Figure 1A, Supplementary Figure 1) Among these highly overlapping USP family genes, USP4, as a gene regulated by super-enhancers (SEs), has been demonstrated to play a significant role in the initiation and progression of AML^31^, USP15 exhibits low specificity, while USP48 has already been investigated in our parallel projects. Therefore, we selected USP20 as the target for further research. Further comparison of H3K27ac ChIP-seq data from AML patients, AML cell lines, and HSPC+ samples using the IGV visualization tool revealed significant enrichment of H3K27ac signals near the USP20 gene in most AML samples and AML cell lines, whereas this enrichment pattern was not observed in HSPC+ samples (Figure 1B, Supplementary Figure 2), it indicates that a potential super-enhancer region may form near the USP20 gene in AML. The above results indicate that a potential super-enhancer region may form near the USP20 gene in AML.

BRD4 is a well-recognized inhibitor of super-enhancers (SEs) and regulates their expression^8^. In MV4-11 and kasumi-1 cells, knockdown of the SE core regulator BRD4 resulted in significantly reduced USP20 mRNA and protein levels, which was confirmed by treatment with the BRD4 inhibitor GNE987 in AML cells, confirming SE-mediated regulation of USP20 in AML (Figure 1C; Supplementary Figure 3A). Analyses of USP20 expression using data from TCGA and GTEX databases revealed that USP20 gene expression was significantly elevated in AML compared to normal tissues (Figure 1D). Furthermore, high USP20 expression were consistently observed across various AML cell lines (Supplementary Figure 3B). Clinically, high USP20 expression correlated with inferior prognosis in AML patients based on data from the TARGET and TCGA databases (Figure 1E, Supplementary Figure 3C). Collectively, these results suggest that USP20 functions as an SE-driven oncogenic factor regulator in AML and can serve as a potential prognostic marker.

### Key AML transcription factors regulate USP20 through super-enhancer binding

**Figure 2:**
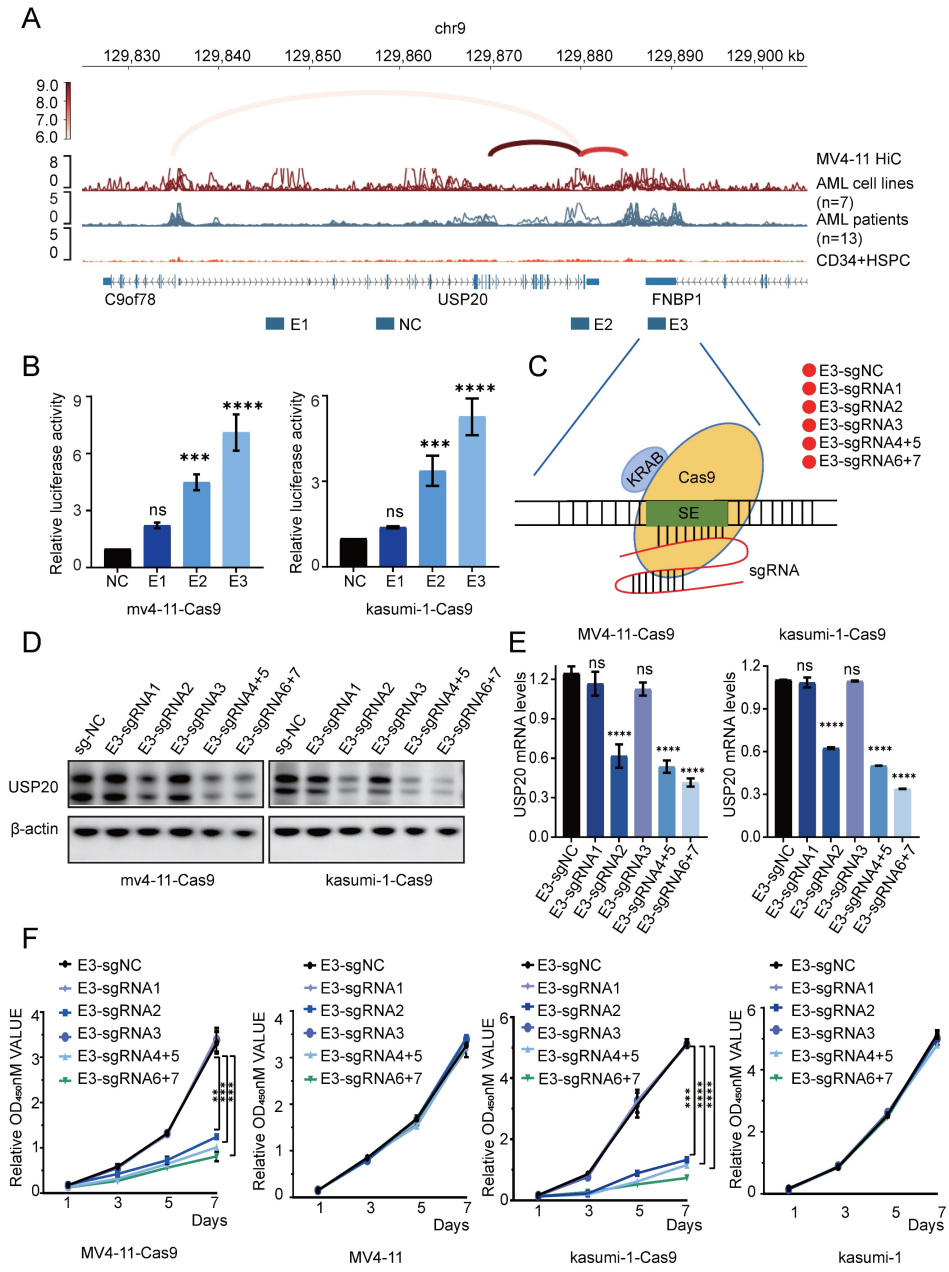
Regulation of USP20 by associated super enhancers. (A) ChIP-seq gene mapping reveals the H3K27ac signal at the USP20 gene locus. The map illustrates Hi-C interactions in acute myeloid leukemia (AML) and identifies three candidate enhancer regions of USP20 (E1, E2, and E3). (B) This study assessed the efficiency of transfecting MV4-11 and kasumi-1 cells with dual-luciferase reporter vectors containing either negative controls or USP20 enhancer elements. (C) A schematic diagram depicts a method for inhibiting the constitutive enhancer of USP20 using an inactive Cas9 fused with a transcriptional inhibitory domain (KRAB). (D) Western blotting results are presented for MV4-11 and kasumi-1 cells that were transfected with the sgRNA-Cas9 vector. (E) The mRNA expression levels of USP20 in MV4-11 and kasumi-1 cells following transfection with the sgRNA-Cas9 vector are shown. (F) Cell viability assessments for MV4-11-Cas9, MV4-11, Kasumi-1-Cas9, and kasumi-1 cells after transfection with non-targeted negative control sgRNA or E3-targeted sgRNA were conducted using the CCK8 assay. no significant difference was observed (ns), * P < 0.05, ** P < 0.01, *** P < 0.001, **** P < 0.0001. All error bars in the text represent standard deviations.

To characterize the interactions between the enhancer and promoter regions of the USP20 gene, we reanalyzed public Hi-C (GSE147123)^32^ data obtained from MV4-11 cell. The results revealed robust long-range chromatin interactions at the USP20 locus. We employed linkers superimposed on the ChIP-seq peak map to illustrate key enhancer-promoter interaction linkers. Ultimately, we pinpointed three potential enhancer components with core functional activity: E1, E2, and E3 within the identified super enhancer region (USP20-SE), additionally, a region devoid of enhancer activity was selected as a negative control (Figure 2A).

To functionally characterize the candidate enhancers, we cloned E1-E3 into the PGL3 promoter luciferase reporter vector and subsequently transfected into the MV4-11 and kasumi-1 cells. Luciferase assays revealed that both E2 and E3 exhibited reporter gene activity, with E3 demonstrating stronger activity than E2, which is consistent with its pronounced H3K27ac signal (Figure 2B). These results identified E3 as the core functional unit of the USP20 super enhancer. To further investigate the transcriptional regulatory function of E3 on USP20, we employed CRISPR-mediated inhibition using sgRNA pairs targeting the USP20 E3 element (Figure 2C). Western blotting (WB) and PCR results indicated that sgRNA2, sgRNA4+5, and sgRNA6+7 significantly reduced both USP20 protein and mRNA levels in MV4-11-Cas9, kasumi-1-Cas9 and CMK-Cas9 cells. (Figures 2D, 2E, Supplementary Figure 5A, 5B). Additionally, cells treated with sgRNA2, sgRNA4+5, or sgRNA6+7 exhibited substantial cell death (Supplementary Figure 4). In MV4-11-Cas9 cells, kasumi-1-Cas9 cells and CMK-Cas9 cells CRISPR-mediated knockout of USP20 super enhancer activity markedly inhibited cell proliferation. In contrast, transfection of sgRNAs alone showed no a significant effect in either MV4-11, CMK or kasumi-1 cells (Figure 2F, Supplmentary Figure 5C), These results demonstrate that the USP20-SE, particularly its E3 component, is essential for USP20 expression and AML cell survival.

**Figure 3:**
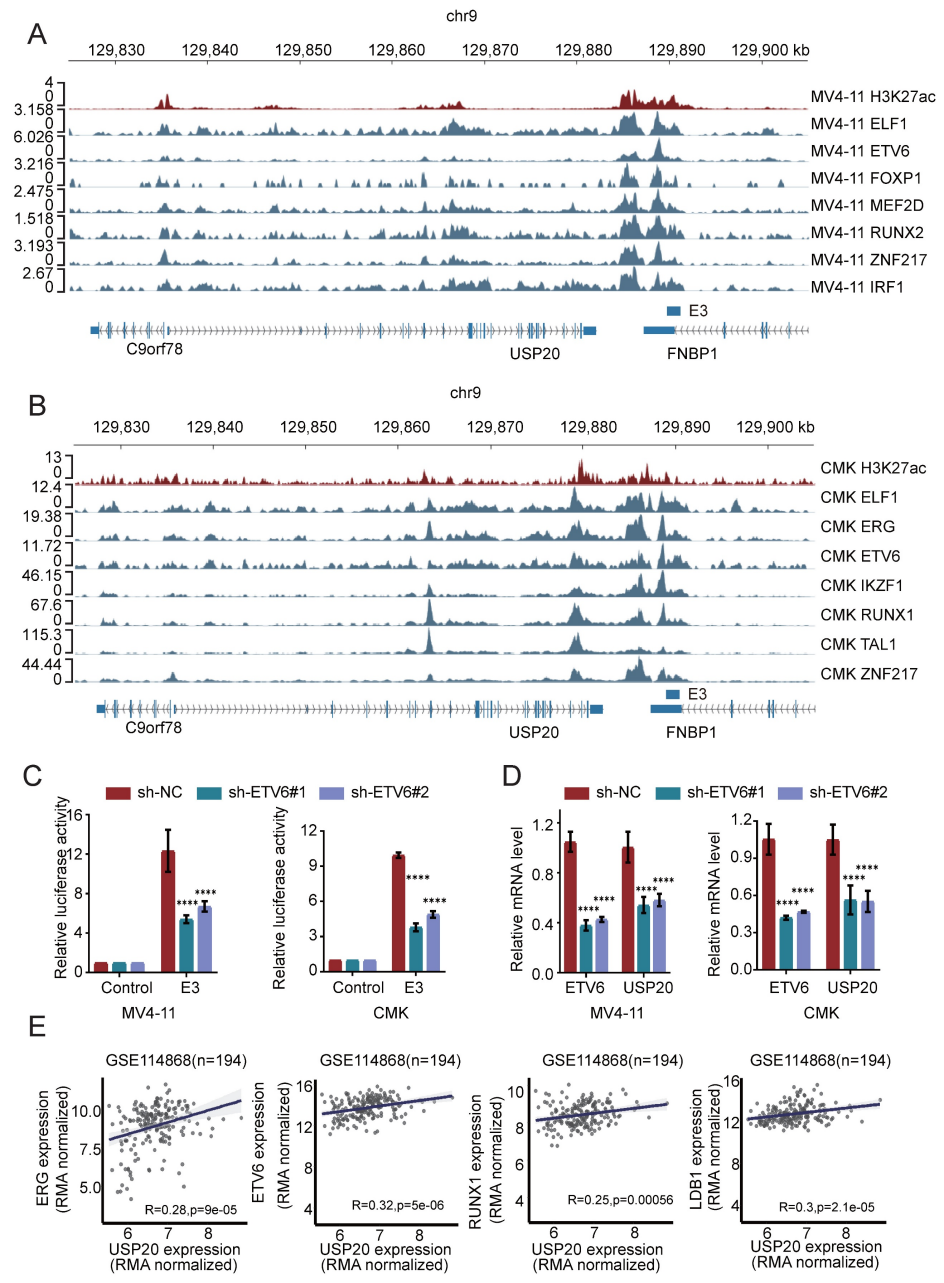
The crucial role of the master transcription factor in regulating super enhancer-driven USP20 expression. (A) The IGV profile from Cut&tag demonstrates the signaling of transcription factors (TFs) within the super enhancer region of the USP20 gene locus in acute myeloid leukemia (AML) cell lines, specifically CMK. (B) The IGV profile from Cut&tag illustrates the signal of transcription factors (TFs) located at the super enhancer region of the USP20 locus in another AML cell line, MV4-11. (C) Assessment of E3 luciferase reporter gene activity following knockdown of ETV6 in both MV4-11 and CMK cells. (D) Quantitative RT-PCR analysis revealed mRNA levels of USP20 in MV4-11 and CMK cells subsequent to ETV6 transcription factor knockdown. (E) A scatter plot indicated a strong positive correlation between transcript levels of USP20, ERG, ETV6, RUNX1, and LDB1 across AML cases (GSE114868; n=194). Data are presented as mean ± standard deviation (mean ± SD); n Data are expressed as mean ± standard deviation (mean ± SD); no significant difference was observed (ns), * P < 0.05, ** P < 0.01, *** P < 0.001, **** P < 0.0001. All error bars in the text represent standard deviations.

According to previous study, highlights the roles of ELF1^33^, ERG^34^, ETV6^35^, IKZF1^36^, RUNX1^35^, TAL1^37^, and ZNF217^38^ in promoting AML proliferation, we then investigated their potential role in regulating USP20 expression. Cut&tag analyses on these master transcription factors were performed in MV4-11 and CMK cells and identified multiple enrichment of ETV6, ELF1, and ZNF217 within the super enhancer region of USP20, with strong peak binding observed at the E3 enhancer region (Figure 3A-B; Supplementary Table 8-14). To investigate whether these key transcription factors directly regulate USP20 expression through super enhancers (SE), we knocked down ETV6, ELF1, and ZNF217 in MV4-11 and CMK cells transfected with a PGL3 promoter luciferase reporter vector containing the USP20-E3 enhancer. Each knockdown significantly reduced reporter activity of USP20-E3 (Figure 3C; Supplementary Figure 5D) and decreased endogenous USP20 mRNA expression in both MV4-11 and CMK cells (Figure 3D; Supplementary Figure 5E). Consistent with these findings, analysis of AML patient data obtained from the GEO database (GSE114868) indicated that USP20 exhibited positive correlations with ERG, ETV6, RUNX1, and LDB1 (Figure 3E). These findings suggest that the ETV6, ELF1, and ZNF217 transcription factors synergistically regulate USP20 expression by directly occupying the super enhancer region.

### Knockdown of USP20 impairs AML cell proliferation *in vivo* and *in vitro*

**Figure 4:**
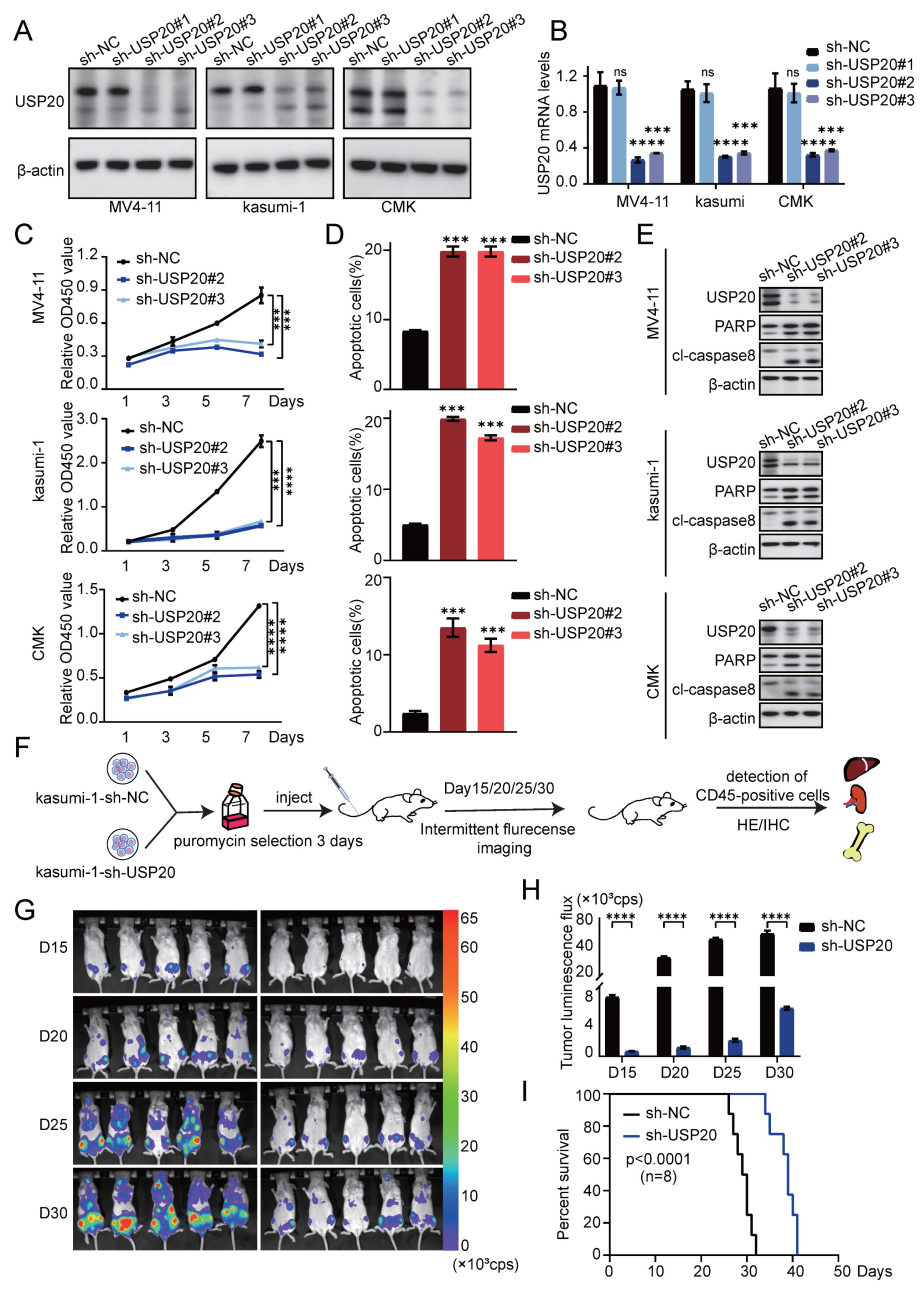
Knockdown of USP20 inhibits proliferation and progression of AML both *in vitro* and *in vivo*. (A) Western blot analysis confirmed the knockdown efficiency of USP20 in MV4-11, kasumi-1, and CMK cells. (B) The mRNA levels of USP20 in MV4-11, Kasumi-1, and CMK cells infected with sh-NC, sh-USP20#2, and sh-USP20#3 were assessed using quantitative reverse transcription PCR (qRT-PCR). (C) The proliferation rates of MV4-11, Kasumi-1, and CMK cells following USP20 knockdown were evaluated through the CCK-8 assay. (D) Apoptosis levels in MV4-11, kasumi-1, and CMK cells after USP20 interference were determined via flow cytometry. (E) Western blot analysis revealed that following the knockdown of USP20, the expressions of PARP and cleaved caspase-8 were upregulated in MV4-11, kasumi-1, and CMK cells. (F) Flowchart illustrating the design of the *in vivo* experimental study. (G) Relevant bioluminescence imaging results for both the USP20 knockdown group and control group mice obtained on days 15, 20, 25, and 30 are presented. (H) The bar chart displays bioluminescence signal values for both mouse groups at various time points. (I) Mice treated with sh-USP20 exhibited a longer survival duration compared to those in the control group. Data are presented as mean ± standard deviation (mean ± SD); no significant difference was observed (ns), * P < 0.05, ** P < 0.01, *** P < 0.001, **** P < 0.0001. All error bars in the text represent standard deviations.

To investigate the role of USP20 in acute myeloid leukemia, we selected MV4-11, kasumi-1, and CMK cells based on the expression levels of USP20 in AML cell lines (Supplementary Figure 3B). *In vitro* experiments were conducted using sh-RNA specifically targeting USP20 (sh-USP20 #1, sh-USP20 #2, sh-USP20 #3) alongside a control sh-RNA (sh-NC). Efficient knockdown of USP20 using specific shRNAs (#2 and #3 showing strongest efficacy) was confirmed at both protein and mRNA levels by Western blot and qRT-PCR respectively (Figures 4A-B). In colony formation assays, depletion of USP20 markedly impaired colony formation capacity (Supplementary Figure 6A). CCK8 assay reveal that compared to the control group (sh-NC), reduction of USP20 significantly inhibited proliferation across all tested cell lines—CMK, MV4-11, and kasumi-1 (Figure 4C). White light imaging confirmed USP20 knockdown (sh-USP20) induced cell death compared to controls (sh-NC) (Supplementary Figure 6B). Propidium iodide staining demonstrated that knockdown of USP20 led to G2/M phase cell cycle arrest (Supplementary Figure 7A) Additionally, flow cytometry results indicated an increase in apoptotic cell populations following knockdown of USP20 (Figure 4D; Supplementary Figure 7B), further supported by Western blot showing increased cleavage of PARP and caspase-8 (Figure 4E), and Bcl-2 protein levels are downregulated (Supplementary Figure 7C). Additionally, the reduction in Cyclin E1 protein levels upon USP20 knockdown provides further evidence that loss of USP20 induces cell cycle arrest (Supplementary Figure 7C), these results collectively demonstrating USP20 plays a critical role in maintaining the proliferative and survival potential of AML cells *in vitro*. We next performed knockout experiments targeting the USP20 protein in primary acute myeloid leukemia cells derived from patients, Western blot and PCR analyses conducted on samples from AML patients and normal controls demonstrated that both protein and mRNA levels were significantly elevated in AML patients relative to normal tissues (Supplementary Figure 8A).” Subsequently, we performed shRNA knockdown experiments in AML patient-derived cells, with the knockdown efficiency illustrated in panel (Supplementary Figure 8B). Flow cytometry results indicated that interference with USP20 expression in these cells led to increased apoptosis (Supplementary Figure 8C). Additionally, propidium iodide staining revealed that USP20 knockdown resulted in cell cycle arrest at the G2/M phase (Supplementary Figure 8D).

We further validated the role of USP20 in acute myeloid leukemia (AML) through *in vivo* experiments. luciferase-labeled kasumi-1 cells with sh-USP20 or sh-NC were intravenously injected into NSG mice (Figure 4F). Bioluminescence imaging revealed significant reduced fluorescence fluxes in mice injected with sh-USP20 compared to those in the control group (sh-NC) at day 15, 20, 25, and 30 post - injection (Figure 4G-H). At the endpoint, decreased fluorescence were observed in liver, spleen, and bone in sh- USP20 mice relative to the control group (Supplementary Figure 9A). Additionally, a lower CD45+ infiltration was detected in the sh-USP20 group than that in the control group (Supplementary Figure 9B). Survival analysis revealed that mice with USP20 knockdown exhibited prolonged median survival (Figure 4I). H&E staining and Ki67 immunohistochemical staining of bone marrow, liver, and spleen demonstrated markedly reduced tumor proliferation compared to controls (Supplementary Figure 9D; Supplementary Figure 10). Together, these findings establish that USP20 depletion substantially inhibits AML proliferation both *in vitro* and *in vivo*.

### Virtual screening of USP20 inhibitors

Protein Preparation and Binding Site Prediction. Considering the absence of reported full-length crystal structures for USP20 in the Protein Data Bank (PDB), the structure utilized for virtual screening was derived from predictions made by AlphaFold 2^39^-^40^. Given that the catalytic activity of the deubiquitinase USP20 is contingent upon the residues Cys154 and His643, we employed MOE to predict potential binding sites on the AlphaFold 2-derived USP20 structure. Site1, which is situated near His643, was selected for virtual screening. The amino acids constituting Site1 include Gln229, Gln230, Asp231, Glu234, Phe589, His591, His635, Thr638, Ala639, Gly640, Ser641, Gly642, His643, and Tyr644 (Supplementary Figure 11A). The preparation of the USP20 structure was conducted using the Protein Preparation Wizard module within Maestro (Schrodinger LLC., New York City; 2021)^41^, encompassing bond order assignment as well as hydrogen addition and disulfide bond assignment at a pH of 7.0 via the PROPKA method^42^-^43^. Energy minimization was performed utilizing the OPLS4 force field with positional restraints to resolve atomic clashes while achieving a heavy atom root mean square deviation (RMSD) convergence of 0.3 Å. Additionally, side chain optimization was carried out to refine side chain conformations resulting in a prepared receptor structure suitable for subsequent screening.

The virtual screening utilized the T001 compound library (TargetMol, comprising 26,836 compounds with known activities). The compound library was processed using the LigPrep module in Maestro, which involved protonation at pH 7.0 ± 2.0 via the Epik method, desalting, tautomer generation, and retention of original atomic chirality. To accommodate global conformational flexibility, up to 32 conformations per molecule were generated. The prepared compound library served as the ligand input for virtual screening. As illustrated in Supplementary Figure 11B, following protein preparation, binding site prediction, and small molecule compound library preparation, molecular docking-based virtual screening was conducted to identify novel small molecules targeting the USP20 protein. The prepared receptor and ligand files were imported into Maestro where Glide SP docking mode was employed to dock each compound. Post-docking energy optimization and glide gscore calculations were performed accordingly^44^-^46^. The top 50% of molecules (13,418 compounds) exhibiting glide gscores ranging from -9.8233 to -4.0739 kcal/mol were retained (Supplementary Figure 11C). Based on affinity distribution analysis, a selection of 262 compounds with glide gscores < -7 kcal/mol was made. Protein-Ligand Interaction Fingerprint (PLIF) analysis revealed interaction frequencies—including hydrogen bonds, ionic bonds, π-π stacking interactions, π-cation stacking interactions, and π-hydrogen stacking interactions—between these compounds and residues of USP20 while excluding van der Waals interactions.

Notably, Asp231 and Glu234 demonstrated higher interaction frequencies compared to other residues (Supplementary Figure 11D). A structural diversity analysis conducted using MOE with Jarvis-Patrick clustering at a 70% similarity threshold classified the 262 compounds into 167 distinct clusters. The highest-affinity compound from each cluster was retained for further evaluation. Through visual inspection of binding poses, adherence to Lipinski's rule of five, and exclusion of sugars, peptides, and structurally unreasonable compounds, we identified nine candidates for bioactivity testing. The final 2D and 3D representations of the interaction between our selected candidate compound AS1517499 and USP20 are illustrated in Supplementary Figure 11E.

**Figure 5:**
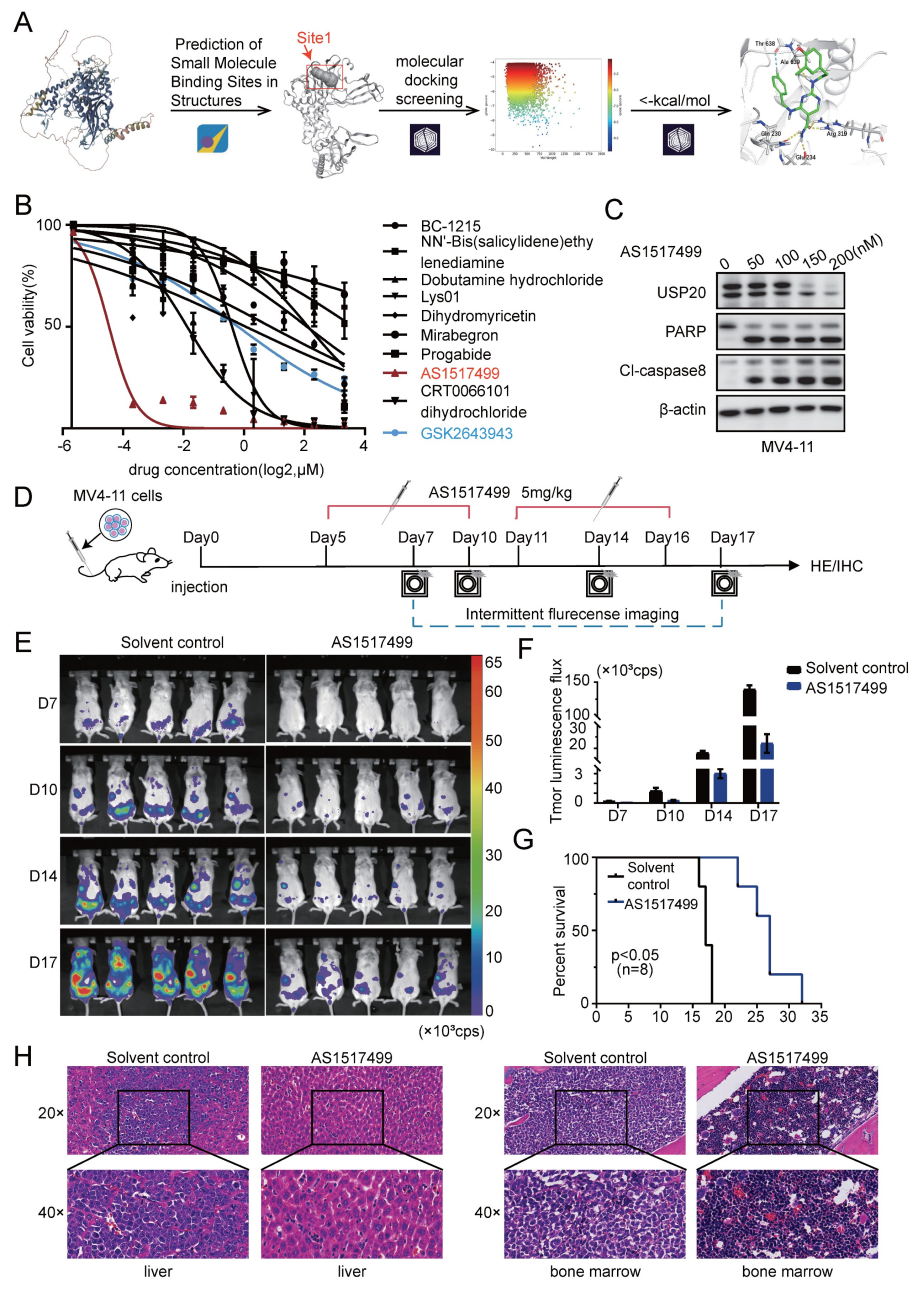
The virtually screened inhibitor AS1517499 demonstrates inhibitory effects on AML proliferation both *in vivo* and *in vitro*. (A) Detailed interaction patterns between compound AS1517499 and USP20 are illustrated, with hydrogen bonds indicated by yellow dotted lines and aromatic hydrogen bonds represented by cyan dotted lines. (B) The impact of nine inhibitors from the virtual compound library, along with the commercial USP20 inhibitor GSK2643943A, on MV4-11 cells was assessed using the CCK-8 assay. Results indicated that AS1517499 exhibited the most potent inhibitory effect on MV4-11 cell growth. (C) Western blot analysis revealed alterations in protein levels of USP20, PARP, and cleaved caspase-8 in MV4-11 cells following treatment with AS1517499. (D) A flowchart depicting the experimental design is presented. (E) Bioluminescence imaging conducted on days 7, 10, 14, and 17 demonstrated significant differences between the AS1517499 treatment group and the solvent control group. (F) The bar chart illustrates bioluminescence signal values for both mouse groups at various time points. (G) Mice treated with AS1517499 exhibited prolonged survival compared to those in the untreated group. (H) HE staining data from mice in both untreated and AS1517499-treated groups are expressed as mean ± standard deviation (mean ± SD). no significant difference was observed (ns), * P < 0.05, ** P < 0.01, *** P < 0.001, **** P < 0.0001. All error bars in the text represent standard deviations.

Through biological activity screening of the nine candidate compounds, we identified AS1517499 as exhibiting the most potent activity among them. To elucidate the structural basis for its optimal efficacy, we performed molecular docking studies of AS1517499 with the USP20 protein using the Ligand Docking module in Maestro, with results illustrated in Figure 5A. Compound AS1517499 exhibited interactions with multiple amino acid residues within Site1: specifically, hydrogen atoms from its amide group formed hydrogen bonds with Gln 230 and Glu 234, while a nitrogen-bound hydrogen atom from the phenethylamine side chain established a hydrogen bond with Ala 639. Additionally, a hydrogen atom on the aromatic ring of the benzylamine side chain formed an aromatic hydrogen bond with Thr 638. Notably, docking results revealed further interactions beyond Site1; specifically, an oxygen atom on the amide group of AS1517499 formed hydrogen bonds with Arg 319. Collectively, these findings suggest that compound AS1517499 may inhibit USP20's deubiquitinating activity by forming multiple hydrogen bonds not only with residues at Site1 but also with peripheral amino acids, thereby obstructing substrate binding to USP20. Through biological activity screening of the nine candidate compounds, we identified AS1517499 as exhibiting the most potent activity among them. To elucidate the structural basis for its optimal efficacy, we performed molecular docking studies of AS1517499 with the USP20 protein using the Ligand Docking module in Maestro, with results illustrated in Figure 5A. Compound AS1517499 exhibited interactions with multiple amino acid residues within Site1: specifically, hydrogen atoms from its amide group formed hydrogen bonds with Gln 230 and Glu 234, while a nitrogen-bound hydrogen atom from the phenethylamine side chain established a hydrogen bond with Ala 639. Additionally, a hydrogen atom on the aromatic ring of the benzylamine side chain formed an aromatic hydrogen bond with Thr 638. Notably, docking results revealed further interactions beyond Site1; specifically, an oxygen atom on the amide group of AS1517499 formed hydrogen bonds with Arg 319. Collectively, these findings suggest that compound AS1517499 may inhibit USP20's deubiquitinating activity by forming multiple hydrogen bonds not only with residues at Site1 but also with peripheral amino acids, thereby obstructing substrate binding to USP20. This interference subsequently affects downstream pathways, ultimately resulting in enhanced biological activity. In summary, compound AS1517499 may inhibit the deubiquitination activity of USP20 by forming multiple hydrogen bonds with both the Site1 residue and adjacent amino acids, thereby obstructing substrate binding to USP20. This disruption further influences downstream pathways, culminating in increased biological activity.

Subsequently, we performed biochemical assays on AS1517499 and USP20 protein, revealing that AS1517499 achieved 50% inhibition of USP20 enzymatic activity at a concentration of 626.7 nM (Supplementary Figure 11F). Next, we performed 100-nanosecond molecular dynamics (MD) simulations using Maestro (Schrodinger LLC., New York City; 2021) with GSK2643943A as the reference ligand. System stability and interaction behavior were evaluated using multiple simulation metrics, including root mean square deviation (RMSD) for overall conformational stability, root mean square flexibility (RMSF) for residue-level fluctuations, and radius of gyration (Rg) for protein compactness (Supplementary Figure 12A-C). Additionally, hydrogen bond analysis monitored the number and persistence of intermolecular hydrogen bonds throughout the simulation, while free energy distributions confirmed thermodynamic equilibrium (Supplementary Figure 12D-E). Results indicate that AS1517499 exhibits comparable binding stability and dynamic interactions with USP20 to those of GSK2643943A. This aligns with in vitro enzyme activity assays (AS1517499 IC_50_=626.7 nM, GSK2643943A IC_50_=160 nM), indicating AS1517499's potential as a novel USP20 inhibitor with anti-AML activity.

### AS1517499, a novel USP20 inhibitor, exhibited ant-leukemic activity in AML

We added the nine compounds (BC-1215, NN'-Bis (salicylidene) ethy Iendiamine, Dobutamine hydrochloride, Lys01, Dihydromyricetin, Mirabegron, Progabide, AS1517499, CRT0066101, dihydrochloride) obtained from Supplementary Figure 11B together with GSK2643943A to MV4-11 cells. The results from the CCK8 assay indicated that AS1517499, a newly identified small molecule inhibitor, demonstrated superior potency with significantly lower IC50 values compared to other candidate inhibitors and the commercial USP20 inhibitor GSK2643943A (Figure 5B, Supplementary Table 15). Treatment with AS1517499 induced decreased USP20 protein in a concentration-dependent manner. In consistent with the effect of USP20 knockdown, AS1517499 treatment promoted cell death (Supplementary Figure 13A), led to an increase in apoptosis of MV4-11 cells with increased cleavage of PARP and caspase-8 were observed upon AS1517499 treatment (Figure 5C; Supplementary Figure 13B), and G2 phase cell cycle arrest (Supplementary Figure 13C). Furthermore, AS1517499 significantly reduced the clonogenic ability of MV4-11 cells in a dose-dependent manner (Supplementary Figure 13D).

Next, we investigated the anti-tumor effects of AS1517499 in a murine AML model established by tail vein injection of luciferase-labeled MV4-11 cells. Subsequently, 5 mg/kg of AS1517499 was administered daily to the experimental group starting five days post-modeling. The leukemic burden was monitored by bioluminescent imaging on days 6, 10, 14, and 17 during the experiment (Figure 5E). The results indicated that the fluorescence flux in AS1517499-treated mice was significantly decreased compared to saline-treated control group (Figure 5F), suggesting that AS1517499 effectively delayed the proliferation of MV4-11 cells *in vivo*. Survival curves demonstrated that AS1517499-treated mice exhibited prolonged survival compared to controls (Figure 5G). At the endpoint, AS1517499-treated mice showed markedly reduced leukemic infiltration in liver, spleen, and bone marrow, demonstrated by lower bioluminescent signals, decreased CD45+ cell frequencies, and diminished tumor cell infiltration on hematoxylin-eosin staining and immunohistochemical analyses (Supplementary Figure 14A-C; Figure 5H; Supplementary Figure 15). Importantly, AS1517499 treatment caused no morphological changes in major tissues or detectable pathological lesions in kidneys and intestines (Supplementary Figure 14D-E), indicating selective anti-leukemic activity with minimal toxicity. Moreover, we measured the body weights of mice in the Solvent control and AS1517499 treatment groups on days 7, 10, 14, and 17. Results showed no significant difference in body weight between the AS1517499 treatment group and the Solvent control group (Supplementary Figure 16A). Additionally, On the fifth day of drug treatment, whole blood and serum were collected from solvent control and AS1517499-treated mice to perform complete blood cell counts and evaluate major indicators of hepatic and renal function. Results demonstrated that white blood cell, red blood cell, and platelet count remained within normal physiological ranges in AS1517499-treated mice, with only a marginal elevation in neutrophil levels (Supplementary Figure 16B). With respect to key markers of liver and kidney function, CREA and UA levels were slightly elevated compared to the control group (Supplementary Figure 16C), but both parameters remained within the normal reference ranges. These findings suggest that AS1517499 exerts minimal hepatotoxic and nephrotoxic effects in mice.

We also obtained results of commercially available USP20 inhibitor GSK2643943A which yielded results consistent with those observed with the virtual screening inhibitor in AML. First, varying concentrations of GSK2643943A significantly inhibited the proliferation of AML cells (Supplementary Figure 17A). a concentration-dependent increase in PARP cleavage was observed in MV4-11 cells treated with GSK2643943A (Supplementary Figure 17B). However, in contrast to AS1517499, GSK2643943A did not reduce USP20 protein levels^49^. Flow cytometry confirmed an increase in apoptosis in GSK2643943A-treated cells (Supplementary Figure 17C). These findings suggest that both virtual screening inhibitors and commercial inhibitors of USP20 can effectively induce cell death in AML; furthermore, our virtual screening inhibitor AS1517499 exhibits a superior anti-leukemic efficacy compared to the commercial compound GSK2643943A.

### Mechanism analysis of USP20 inhibition in AML pathogenesis

**Figure 6:**
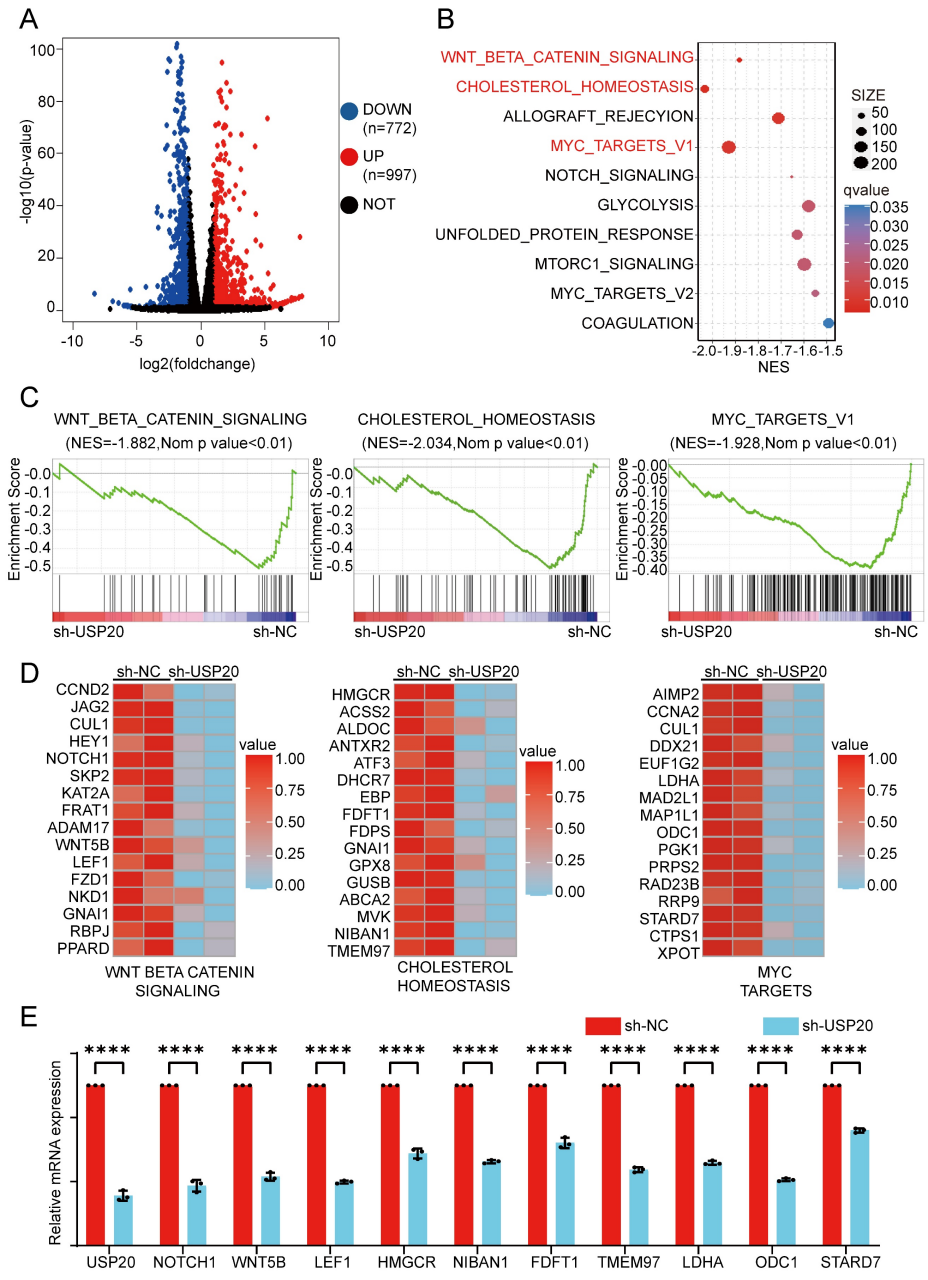
Analysis of the molecular mechanisms by which USP20 inhibits proliferation and progression in AML cells. (A) Differentially expressed genes between the USP20 knockdown group and the untreated group were analyzed using RNA-seq data, represented in a volcano plot; up-regulated genes are indicated in red, while down-regulated genes are shown in blue. (B) KEGG pathway enrichment analysis was performed. (C) Gene set enrichment analysis (GSEA) profiles demonstrated that genes were significantly enriched in the WNT-BETA-CATENIN, cholesterol, and MYC signaling pathways following USP20 knockdown in AML cells. (D) The heat map illustrates the expression levels of genes within the WNT-BETA-CATENIN, cholesterol, and MYC pathways in CMK cells subjected to USP20 knockdown. (E) qPCR analysis revealed decreased expression levels of related genes such as CCND2, HMGCR, and AIMP2. (F) Following USP20 knockdown in AML cells, PCR result analysis indicated downregulation of gene expression across the WNT-BETA-CATENIN, cholesterol, and MYC pathways. Data are presented as mean ± standard deviation (mean ± SD); no significant difference was observed (ns), * P < 0.05, ** P < 0.01, *** P < 0.001, **** P < 0.0001. All error bars in the text represent standard deviations.

To investigate the transcriptional regulation of USP20 in AML, we conducted RNA-seq transcriptome sequencing on USP20-knockdown CMK cells verse control CMK cells. Differential expression analysis identified 772 genes downregulated and 997 upregulated genes in the USP20 knockdown group, (|log2FoldChange|> 1.0 and p < 0.05) (Figure 6A, Supplementary Table 16). Gene enrichment analysis (GSEA) of the downregulated genes indicated significant suppression in of WNT/β-catenin, cholesterol, and MYC pathways (Figures 6B-C). The alterations in expression patterns of key genes associated with these pathways following USP20 knockdown were further illustrated in Figure 6D. Dysregulation of the WNT pathway has been well-established in AML^47^. notably, CTNNB1 expression is markedly elevated in AML cells compared to normal tissues according to data from TARGET and TCGA databases. Furthermore, high CTNNB1 leveld are correlated with poor prognosis (Supplementary Figures 18A-B), suggesting USP20-mediated regulation of β-catenin may contribute to AML pathogenesis.

As a master regulator of cellular metabolism, MYC serves as a prototypical oncogene that disrupts and reprograms various facets of cellular metabolism in AML, encompassing glucose uptake and glycolysis, glutamine catabolism, serine/glycine metabolism, and lipid biosynthesis^51^. Our RT-qPCR analysis indicated that USP20 knockdown significantly reduced expression of key metabolic regulators of WNT-BETA-CATENIN, cholesterol, and key MYC pathway genes such as NOTCH1, HMGCR, and LDHA (Figure 6E). These findings suggest USP20 as a critical modulator of oncogenic signaling in AML, particularly through its regulation of the WNT/β-catenin pathway.

### USP20 stabilizes CTNNB1 and SQSTM1 through deubiquitination in AML

**Figure 7:**
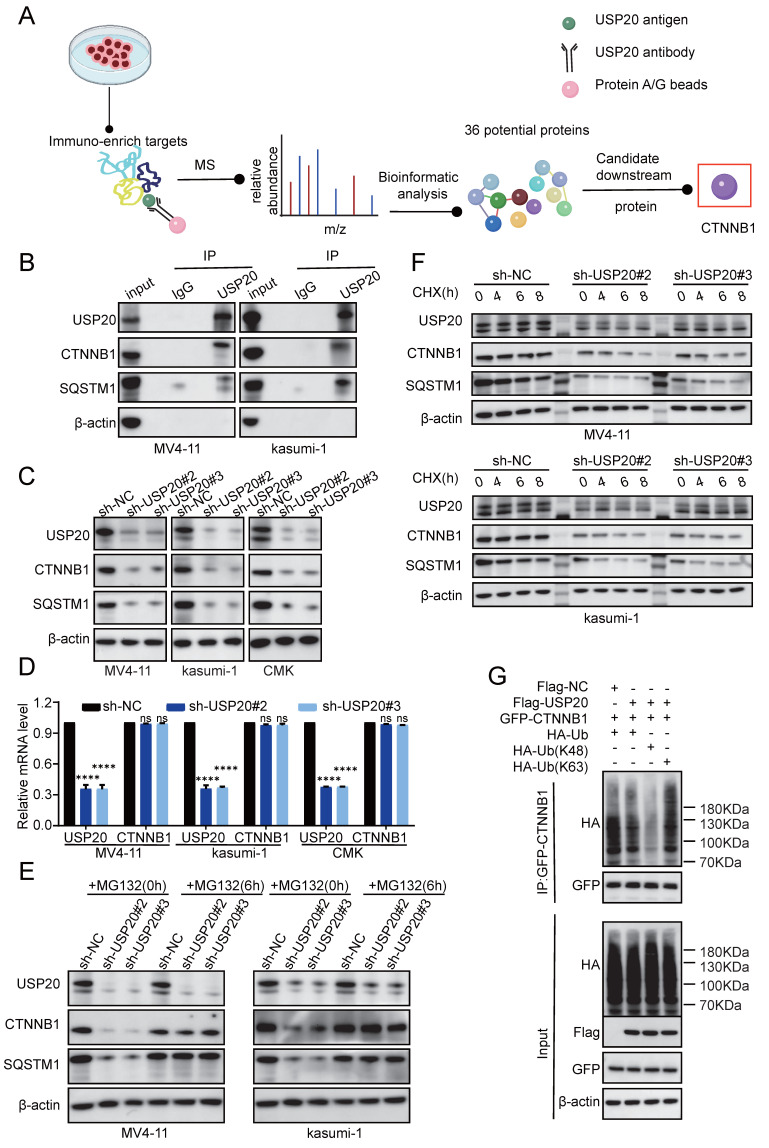
USP20 facilitates AML progression by modulating deubiquitination of CTNNB1 and SQSTM1. (A) Schematic representation of the mass spectrometry analysis. (B) Co-immunoprecipitation assays were conducted in MV4-11 and kasumi-1 cells overexpressing USP20, followed by Western blotting to detect the proteins USP20, CTNNB1, and SQSTM1. (C) The protein levels of USP20, CTNNB1, SQSTM1, and β-actin in MV4-11 kasumi-1 and CMK cells stably expressing sh-NC, sh-USP20#2, and sh-USP20#3 were assessed using Western blotting (WB). (D) mRNA expression levels of USP20 and CTNNB1 in MV4-11 and kasumi-1 cells stably expressing sh-NC, sh-USP20#2, and sh-USP20#3 were quantified through qRT-PCR. (E) Following a 6-hour incubation with 20 μM MG-132 in MV4-11 and kasumi-1 cells stably expressing sh-NC, sh-USP20#2, or sh-USP20#3, the protein levels of USP20, CTNNB1, SQSTM1, and β-actin were analyzed via Western blotting. (F) After treatment with 100 μg/ml actinomycin (CHX) for a specified duration on MV4-11 and kasumi-1 cells expressing either sh-NC or the two different USPs (sh-USP20#2 or #3), we evaluated the protein levels of USP20, CTNNB1, SQSTM1, and β-actin using Western blotting. (G) MG132 (at a concentration of 20 μM) was administered to HEK293FT cells transfected with Flag-USP20, GFP-CTNNB1, wild-type ubiquitin, Ubiquitin mutant retaining only lysine at position 48(Ub-K48), and Ubiquitin mutant retaining only lysine at position63(Ub-K63). This treatment occurred six hours prior to harvesting. Subsequently, the ubiquitination status of CTNNB1 was determined. Data are presented as mean ± standard deviation (mean ± SD); no significant difference is indicated as ns; * P < 0.05; ** P < 0.01; *** P < 0.001; **** P < 0.0001. All error bars in the text represent standard deviations.

To further investigate the regulatory mechanisms of USP20 in these pathways, mass spectrometry analysis was performed and identified 36 potential USP20-interacting proteins, including H2AC14, SQSTM1, H2BC13, CTNNB1, and HIF1A (Supplementary Table 17). Based on unique peptides, coverage, and intensity metrics, CTNNB1 and SQSTM1 were selected as primary targets for further study (Figure 7A). Subsequently, co-immunoprecipitation (CO-IP) in USP20 overexpressing MV4-11 and kasumi-1 cells confirmed the interaction between USP20 and CTNNB1 and SQSTM1 (Figure 7B). Notably, knockdown of USP20 led to decreased levels of both CTNNB1 and SQSTM1 proteins; but not mRNA, levels In MV4-11, kasumi-1, and CMK cells (Figures 7C-7D; Supplementary Figure 19A), indicating that the regulation of CTNNB1 and SQSTM1 by USP20 was not at RNA levels. Additionally, AS1517499 treatment resulted in concentration-dependent reductions in protein levels of both CTNNB1 and SQSTM1 (Supplementary Figure 19B). Given that USP20 functions as a deubiquitinating enzyme, we hypothesize that it may stabilize these proteins by modulating their ubiquitination status. Treatment with the proteasome inhibitor MG132 reversed the downregulation of CTNNB1 and SQSTM1 protein In MV4-11, kasumi-1, and CMK cells with USP20 knockdown (Figure 7E; Supplementary Figure 19C), demonstrating that USP20 post-translationally stabilizes these oncoproteins by preventing their ubiquitin-mediated degradation.

**Figure 8:**
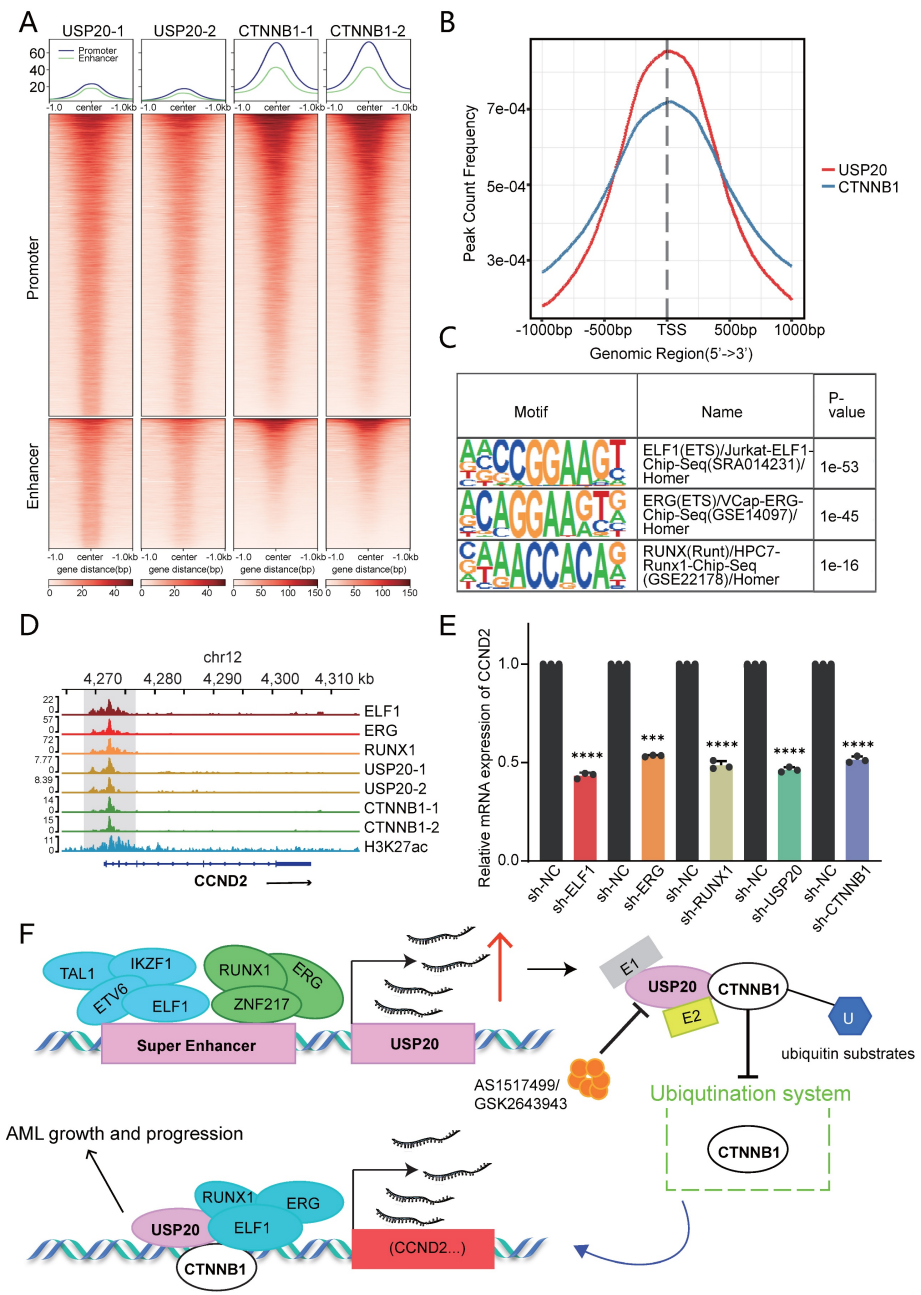
USP20 regulates gene transcription through CTNNB1. (A) Heat maps generated from the Cut&tag study demonstrate that USP20 and CTNNB1 occupy overlapping genomic loci in CMK cell lines. (B) The distribution of binding peaks for CTNNB1 and USP20 within ±1000 bp intervals surrounding the USP20 binding site is illustrated. (C) Cut&tag analysis reveals that USP20 exhibits highly similar binding motifs to ELF1, ERG, and RUNX1. (D) The IGV profile from Cut&tag shows signals for ELF1, ERG, RUNX1, USP20, CTNNB1, and H3K27ac at the CCND2 gene locus. (E) PCR analysis indicates mRNA expression levels of CCND2 following knockdown of ELF1, ERG, RUNX1, USP20, and CTNNB1. (F) Data representing mechanism pattern graphs are expressed as mean ± standard deviation (mean ± SD); no significant differences were observed (ns), * P < 0.05, ** P < 0.01, *** P < 0.001, **** P < 0.0001. All error bars in the text represent standard deviations.

The protein synthesis inhibitor actinomycin (CHX), accelerates the degradation of CTNNB1 and SQSTM1 protein levels in USP20-knockdown MV4-11 and kasumi-1 cells (Figure 7F, Supplementary Figure 19E-F), confirming USP20's role in stabilizing these proteins. Given USP20's function as a deubiquitinating enzyme, we hypothesize that it maintains CTNNB1 stability by inhibiting K48-linked polyubiquitination. To validate this hypothesis, we co-expressed Flag-empty vector/Flag-USP20, GFP-CTNNB1, wild-type ubiquitin (Ub-WT), K48 site-specific ubiquitin (Ub-K48), and K63 site-specific ubiquitin (Ub-K63) in MV4-11 cells. Western blot analysis revealed that overexpression of USP20 significantly reduced the total CTNNB1 ubiquitination (Figure 7G lane 1 vs lane 2) and specifically inhibiting K48 linked polyubiquitination while sparing K63-linked chains (Figure 7G lane 3 vs lane 4). And according to previous research, USP20 binds directly to the N-terminal region (amino acids 1-97) of CTNNB1 through its UCH domain (located at amino acids 301-480), leading to the deubiquitination and subsequent stabilization of CTNNB1^48^. Cut&tag analysis further confirmed substantial genomic co-localization between USP20 and CTNNB1 (Figure 8A-B; Supplementary Figures 20A-B; Supplementary Tables 18-21).

Cut&tag analysis revealed that USP20 shared highly similar motifs to ELF1, ERG1, and RUNX1 (Figure 8C). Furthermore, these factors exhibit substantial genomic co-occupancy with CTNNB1 in CMK cells (Supplementary Figures 21A-B). IGV visualization demonstrated that USP20 can co-regulate the transcription of Wnt-beta-catenin pathway genes, including CCND2, JAG1, and ADAM17 through its co-localization with CTNNB1, ELF1, ERG, and RUNX1 (Figure 8D; Supplementary Figures 21C-D). Consistent with this, knockdown of any of the genes—USP20, CTNNB1, ELF1, ERG or RUNX1—in MV4-11 cells leads to a reduction in mRNA levels of these target genes (Figure 8E; Supplementary Figures 21E-F). According to the Cancer Dependency Map, knockout of CCND2 in MV4-11 and kasumi-1 cells significantly suppresses cancer cell proliferation (Supplementary Figures 22). Together, these findings establish USP20 as a super-enhancer regulatory gene that promotes AML progression by informing a transcriptional regulatory network with CTNNB1, ERG, ELF1, and RUNX1 (Figure 8F).

## Discussion

Previous research conducted by Matthew Meyerson et al. indicates that in addition to enhancer hijacking, focal amplification of SE drives abnormal oncogene expression which include several cancer-related genes such as KLF5, USP12, PARD6B, and MYC^49^. Our study investigates the function and mechanisms of SE-drivern genes in acute myeloid leukemia (AML). In AML, Prdm16 acts on the super enhancers associated with myeloid transcription factors, reprogramming megakaryocyte progenitor cells into AML stem cells^10^. SE inhibitors have been shown to effectively suppress SE-regulated genes—such as MCL-1, MYC, and cyclin D1—thereby reversing the progression of AML and exerting anti-tumor effects^50^. These insights position SEs targeting as a promising strategy for AML.

However, the regulation of USP gene by SE remains unexplores. Our H3K27ac ChIP-seq analysis across seven AML cell lines and thirteen AML patient samples revealed USP20 as a SE-associated USP family member. Previous studies have indicated that USP4 can enhance ribosomal biogenesis and protein synthesis in hematopoietic stem cells (HSCs) and leukemia cells by stabilizing PES1, thereby facilitating leukemia progression^51^. In our study, we found that USP20 is an SE-regulated gene in AML. Additionally, we found that key AML transcription factors—such as ELF1, ETV6, and ZNF217—are also regulated by USP20. Clinical data confirmed positive correlations between AML-associated transcription factors ERG, ETV6, RUNX1, LDB1 and USP20. Functional validation demonstrated knockdown of these transcription factors reduced USP20-E3 enhancer activity and USP20 expression. Our data suggest that transcription factors such as ELF1, ETV6, and ZNF216 may drive USP20 overexpression through SE-mediated regulation in AML.

As a specific deubiquitinating enzyme, USP20 plays a crucial physiological role in antiviral immunity, cancer, metabolic disorders, and neurological diseases. USP20 can suppresses angiogenesis by inhibiting the NF-κB signaling pathway and the inhibition of phosphorylation at Ser334 of USP20 has been shown to have an impact on alleviating atherosclerosis^52^. In hepatocellular carcinoma, high levels of USP20 expression promotes ferroptosis resistance and reduce the toxicity of oxaliplatin (OXA) to hepatocellular carcinoma cells^53^. Additionally, USP20 is implicated in promoting metastasis in colorectal cancer (CRC) cells^54^ and in breast cancer models, USP20 has been observed to facilitates breast cancer lung colonization^55^. However, USP20 expression in gastric cancer tissues is negatively correlated with tumor size, invasiveness, and TNM stage, indicating a tumor-suppressive role of USP20 in gastric cancer^56^. This implies that, depending on the cellular context or oncogenic signaling environment, USP20 may target distinct substrates for deubiquitination. In acute myeloid leukemia (AML), the unique microenvironment may favor USP20-mediated stabilization of oncogenic proteins such as CTNNB1 and SQSTM1. However, the role of USP20 in AML remains unclear. Our study indicates that silencing of USP20 significantly enhances apoptosis, cell cycle arrest and impairs the colony-forming ability of AML cells. In AML mouse models, knockdown of USP20 prolonged survival time. Collectively, these experimental findings suggest that USP20 is a critical driver of AML progression. Therefore, our study represents the first identification of USP20 as an oncogenic factor in AML. This finding not only broadens the understanding of the functional complexity of USP20 but also lays a theoretical foundation for the development of precise and low-toxicity therapeutic strategies against AML.

The USP20 inhibitor, GSK2643943A, has been shown to overcome treatment resistance in oral squamous cell carcinoma^57^ and to reduce postprandial cholesterol levels, thereby addressing metabolic disorders^50^. our study revealed its pro-apoptosis effect in AML by inducing cleavage of PARP protein. However, our virtual screening-identified compound AS1517499 exhibits superior potency with a lower IC50 than GSK2643943A. AS1517499 not only enhances apoptosis in AML cells but also induces cell cycle arrest at significantly lower concentrations compared to GSK2643943A. *In vivo* studies demonstrate that AS1517499 markedly extends survival in AML mouse models with minimal toxicity and side effects. Our data suggest that AS1517499 represents a more promising USP20-targeted therapeutic candidate with enhanced and improved safety profile. AS1517499 may also be combined with conventional chemotherapy drugs currently in use, such as cytarabine, or with BET inhibitors in the future to pioneer a new treatment approach for AML. We employed the MV4-11 cell line to construct a mouse xenograft model for evaluating the therapeutic efficacy of AS1517499. While this approach remains a standard method for assessing tumor suppression in both past and current studies, the highly heterogeneous nature of human tumors means that a single-source tumor cell line struggles to reflect tumor heterogeneity and dynamic evolution. Furthermore, the mouse immune system cannot fully mimic the human immune environment, potentially leading to misinterpretations of tumor biology. In contrast, PDX models involve transplanting patient tumor tissue into immunodeficient or humanized mice to allow its growth within the mouse^58^. In contemporary research, PDX models have proven to be powerful tools for conducting cancer gene inventories, experimental studies, and testing the value of tumors as drug targets^59^. PDX models demonstrate superiority in reproducing cancer's spatial architecture and intratumoral heterogeneity^60^. They preserve the microenvironment of the parental tumor—including infiltrating lymphocytes, extracellular matrix, and microvasculature surrounding tumor cells—facilitating better expression of parental tumor characteristics while maintaining tumor heterogeneity. Moreover, these models exhibit high similarity (over 90%) to the patient's original tumor^61^. Using PDX models allows preservation of the primary tumor for subsequent application studies and reflects sample variations from different tumor sources^62^. However, due to time constraints, we were unable to obtain suitable AML patient specimens. Furthermore, based on our preliminary experiments, the failure rate of AML PDX models is extremely high. Therefore, we are unable to present results evaluating treatment efficacy using PDX models in this study.”

USP20 drives tumor progression through multiple oncogenic mechanisms across cancer types. USP20 promotes NF-κB signaling by removing K48-linked ubiquitin chains, thereby enhancing tumor survival and conferring drug resistance^63^. In breast cancer, USP20 enhanced migratory capabilities by deubiquitinating members of the MAPK family including lysine-free ERK3 mutant^64^. Furthermore, USP20 has been shown to deubiquitinate and stabilize β-catenin protein, promoting cancer cell proliferation, invasion, and resistance to chemotherapy^65^. Moreover, in colorectal cancer models, USP20 promotes disease metastasis and invasion by stabilization of SOX4 protein^66^. In this study, our mass spectrometry analysis and co-immunoprecipitation (CO-IP) studies identified CTNNB1 and SQSTM1 as key substrates of USP20, demonstrating USP20's role in stabilizing oncoproteins through K48-linked deubiquitination in AML.

Our studies demonstrate that knockdown of USP20 or pharmacological inhibition with AS1517499 in AML cells can enhance the stability of CTNNB1 and SQSTM1. Ubiquitination assays confirmed that USP20 stabilizes the CTNNB1 protein by modulating its K48 ubiquitin chain. Given the critical role of Wnt/β-catenin signaling pathway in self-renewal of leukemic stem cells (LSCs) derived from hematopoietic stem cells (HSCs) or more differentiated granulocyte-macrophage progenitor cells (GMPs)^67^, particularly in certain oncogene-transformed progenitor cells, we investigate USP20 and CTNNB1 interactions using Cut&tag techniques, revealing their co-localization in the nucleus. Notably, they share binding motifs with ELF1, ERG, and RUNX1. This indicates that USP20, CTNNB1, ELF1, ERG, and RUNX1 collectively localize in the nucleus as complex that cooperatively regulates the Wnt/β-catenin signaling pathway and its downstream target CCND2. Our experimental data suggest that CTNNB1 serves as a key target of USP20 in AML progression. These findings enhance our understanding of how USP20 contributes to tumorigenesis and propose that SE-USP20-CTNNB1 axis that drives leukemogenesis of AML.

## Conclusion

In summary, our study indicated that USP20 is a gene regulated by super-enhancers and acts as a prognostic marker in AML. USP20 promotes the survival of AML cells by stabilizing the CTNNB1 protein through its by removing K48-linked polyubiquitination chains. Furthermore, USP20 regulates oncogenic transcripts through genomic collaboration with CTNNB1 and other transcription factors. AS1517499, an USP20 inhibitor identified through virtual screening, demonstrated anti-leukemic effects comparable to those observed with USP20 knockdown while exhibiting fewer side effects. Therefore, these findings highlight USP20's role in AML pathogenesis and its therapeutic potential, with AS1517499 as a promising lead compound, uncovering a novel vulnerability in AML.

## Supplementary Material

Supplementary legends.

Supplementary figures.

Supplementary tables.

## Data Availability

The data that support the findings of this study are available on reasonable request from the corresponding author. USP20 RNA-seq and Cut&tag data have been submitted to the GEO database with GSE293347 and GSE293465. ELF1, ERG, ETV6, IKZF1, RUNX1, TAL1, ZNF217 Cut&tag data have been submitted to the GEO database with GSE294263. CTNNB1 Cut&tag data have been submitted to the GEO database with GSE299364. The mass spectrometry proteomics data have been deposited to the ProteomeXchange Consortium via the PRIDE partner repository with the dataset identifier PXD066445.
