# Supplementary material for: USP20, a Super-enhancer Regulated Gene, Promotes Acute Myeloid Leukemia Progression through CTNNB1 Deubiquitination
Source: Int J Biol Sci. 2026 Feb 11;22(5):2665–86. doi: 10.7150/ijbs.122898 (PMC12965243; doi:10.7150/ijbs.122898)
Supplement: Supplementary file 1 — Supplementary legends. [file ijbsv22p2665s1.pdf]

Supplementary Figure 1: Heatmap of H3K27ac chromatin immunoprecipitation sequencing (ChIP-seq) data analyzed using the ROSE method for 66 AML patients and four HSPC+ samples.

Supplementary Figure 2: Comparison of H3K27ac ChIP-seq results from 66 AML patients and four HSPC+ samples using the IGV visualization tool.

Supplementary Figure 3: (A) Evaluation of USP20, BRD4, and β-actin protein levels via Western blotting following treatment with the BRD4 inhibitor GNE987 and knockdown of BRD4. (B) Assessment of USP20 expression levels in AML cell lines using Western blotting. The correlation between USP20 expression levels and prognosis was analyzed using the TARGET database.

Supplementary Figure 4: Survival analysis of MV4-11-Cas9 and kasumi-1-Cas9 cells post-transfection with non-targeting negative control sgRNA versus E3-targeted sgRNA, evaluated through white light and fluorescence imaging.

Supplementary Figure 5: (A) Western blot results of CMK cells transfected with sgRNA-Cas9 vectors. (B) USP20 mRNA expression levels in CMK cells after transfection with sgRNA-Cas9 vectors. (C) Proliferation curves of CMK cells following transfection with non-targeting negative control sgRNA or E3-targeting sgRNA, detected using the CCK8 assay. (D) The activity of the E3 luciferase reporter gene was assessed after individual knockdown of ELF1 and ZNF217 in MV4-11 and CMK cells. (E) qRT-PCR analysis demonstrated mRNA levels of USP20 in MV4-11 and CMK cells following knockdown of transcription factors ELF1 and ZNF217, respectively. Data are presented as mean ± standard deviation (mean ± SD); no significant difference (ns), * $P < 0.05$, ** $P < 0.01$, *** $P < 0.001$, **** $P < 0.0001$. All error bars in the text represent standard deviations.

Supplementary Figure 6: Knockdown of USP20 inhibits proliferation and progression of AML *in vitro*. (A) Colony formation assay results for MV4-11, kasumi-1, and CMK cells transfected with sh-USP20#2, sh-USP20#3, or sh-NC were presented here. (B) Micromorphological changes observed in MV4-11, kasumi-1, and CMK cells subsequent to USP20 knockdown are depicted above. Data are expressed as mean ± standard deviation (mean ± SD); no significant difference (ns), * $P < 0.05$; ** $P < 0.01$; *** $P < 0.001$; **** $P < 0.0001$. All error bars in the text represent standard deviations.

Supplementary Figure 7： (A) Cell cycle distribution profiles measured by propidium iodide (PI) staining following USP20 knockdown in MV4-11, kasumi-1, and CMK cells were illustrated here. (B) Apoptosis levels in infected MV4-11, kasumi-1, and CMK cells were determined using flow cytometry analysis. (C) Western blotting revealed that Bcl-2 and Cyclin E1 were downregulated in MV4-11, kasumi-1, and CMK cells following USP20 knockdown.

Supplementary Figure 8: (A) Validation of USP20 protein and mRNA expression differences between AML patients and non-AML patients using Western blot and qRT-PCR. (B) Western blot analysis confirms the efficiency of USP20 knockdown in AML patient blast cells. (C) Flow cytometry detection of apoptosis levels in AML patient cells following USP20 interference. (D) PI staining results demonstrate the impact of USP20 knockdown on the cell cycle of AML patient blast

cells.

Supplementary Figure 9: Knockdown of USP20 Inhibits Proliferation and Progression of Acute Myeloid Leukemia (AML) *in vivo*. (A) Live bioimaging images depicting the mean radiation uptake in the liver, spleen, and bone tissue across both groups of mice. The bar chart illustrates bioluminescent signal values recorded in the liver, spleen, and bone. (B) Following USP20 knockdown, a significant reduction was observed in the number of human CD45 positive cells within the liver, spleen, and bone marrow when compared to the control group. (C) No significant weight loss was noted between the two groups of mice. (D) Representative immunohistochemical staining patterns for Ki67 in the liver, spleen, and bone marrow tissues from mice are presented. Data are expressed as mean ± standard deviation (mean ± SD); no significant difference was found (ns), * $P < 0.05$, ** $P < 0.01$, *** $P < 0.001$, **** $P < 0.0001$. All error bars in the text represent standard deviations.

Supplementary Figure 10: Hematoxylin-eosin (H&E) staining analysis revealed a marked reduction in tumor cell presence within the liver, spleen, and bone marrow of mice subjected to USP20 knockdown compared to those in the control group. Data are presented as mean ± standard deviation (mean ± SD); no significant difference was found (ns), * $P < 0.05$, ** $P < 0.01$, *** $P < 0.001$, **** $P < 0.0001$. All error bars in the text represent standard deviations.

Supplementary Figure 11: Virtual Screening Process for USP20 Inhibitors (A) Predicted Structure and Small Molecule Binding Site of USP20 (B) Virtual Screening Funnel (C) Molecular Weight-Score Distribution of Compounds. This graph illustrates the molecular weight-score distribution of the top 50% small molecules from the T001 compound library alongside their corresponding USP20 docking scores. (D) PLIF Analysis of 262 Selected Compounds. Identical amino acids are represented by the same color, with a higher vertical density indicating a greater frequency of interaction with the compounds. (E) Binding Conformation of Compound AS1517499 with the USP20 Structure. (1) An overall view depicting the binding site of compound AS1517499 on the USP20 structure, where carbon atoms are shown in green, oxygen in red, and nitrogen in blue; the protein is illustrated as a grey cartoon representation. (2) A close-up 3D view showcasing compound AS1517499 bound to the USP20 structure, highlighting carbon atoms in green, oxygen in red, and nitrogen in blue; surrounding amino acids are depicted as white sticks and labeled in black. Yellow dashed lines indicate hydrogen bonds while cyan dashes represent arene-H interactions. (3) A close-up 2D view illustrating compound AS1517499 bound to the USP20 structure, with purple arrows denoting hydrogen bond interactions. (F) Half-maximal inhibitory concentration curve of AS1517499 against USP20 protein activity.

Supplementary Figure 12: Molecular Dynamics (MD) Simulations of GSK2643943A and AS1517499 with USP20 (A) RMSD plot of the USP20–ligand complexes over 100 ns. (B) RMSF of USP20 residues in complex with AS1517499 and GSK2643943A. (C) Rg of USP20 in complex with AS1517499 and GSK2643943A. (D) Hydrogen bond analysis between USP20 and the ligands over 100 ns. (E) The Gibbs free energy landscape of USP20–ligand complexes.

Supplementary Figure 13: (A) Micromorphological alterations observed in MV4-11 cells post-treatment with AS1517499 are illustrated. (B) Flow cytometry analysis demonstrated that treatment with AS1517499 in MV4-11 cells resulted in a dose-dependent increase in apoptotic cell populations. (C) Cell cycle distribution was assessed using propidium iodide staining following AS1517499 treatment of MV4-11 cells. (D) Colony formation assay results for MV4-11 cells treated with AS1517499 are presented. Data are presented as mean ± standard deviation (mean ± SD); no significant difference was found (ns), * $P < 0.05$, ** $P < 0.01$, *** $P < 0.001$, **** $P < 0.0001$. All error bars in the text represent standard deviations.

Supplementary Figure 14: The USP20 inhibitor AS1517499 effectively inhibits the proliferation and progression of acute myeloid leukemia (AML) *in vivo*. (A) *In vivo* bioimaging revealed average radiation uptake levels in the liver, spleen, and bone tissues across two groups of mice. The accompanying bar chart displays bioluminescent signal values from these organs. (B) A significant reduction in the number of human CD45-positive cells within the bone marrow was observed in the AS1517499 treatment group compared to the control group. (C) Hematoxylin-eosin (H&E) staining analysis was conducted on liver, spleen, and bone marrow samples from both the AS1517499-treated group and solvent control group. (D) Comparative assessment of organ sizes—liver, spleen, and bone marrow—between the AS1517499-treated group and solvent control group is provided. (E) H&E staining analysis of intestinal and renal tissues indicated no damage to these organs among mice treated with AS1517499 when compared to controls. Data are expressed as mean ± standard deviation (mean ± SD); no significant difference was found (ns), * $P < 0.05$, ** $P < 0.01$, *** $P < 0.001$, **** $P < 0.0001$. All error bars in the text represent standard deviations.

Supplementary Figure 15: (A) Body weight curves of mice in the solvent control group and AS1517499 treatment group. (B) Blood Count Reports for Solvent Control and AS1517499 Treatment Groups. (C) Hepatic and Renal Biochemical Indicators in the Blood of Mice in the Solvent Control Group and AS1517499 Treatment Group

Supplementary Figure 16: Representative immunohistochemical staining for Ki67 in the liver, spleen, and bone marrow of mice treated with AS1517499 compared to the solvent control group.

Supplementary Figure 17: (A) The effects of the GSK2643943A inhibitor on AML cells were assessed using a CCK-8 assay to determine its IC50 values. (B) Flow cytometry analysis demonstrated that treatment with GSK2643943A induced apoptosis in MV4-11 cells in a dose-dependent manner. (C) Western blotting revealed alterations in PARP protein levels in MV4-11 cells following treatment with GSK2643943A. Data are expressed as mean ± standard deviation (mean ± SD); no significant difference was found (ns), * $P < 0.05$, ** $P < 0.01$, *** $P < 0.001$, **** $P < 0.0001$. All error bars in the text represent standard deviations.

Supplementary Figure 18: Elevated expression of CTNNB1 in AML cells is associated with poor prognosis. (D) Analysis from public databases indicates that mRNA expression levels of CTNNB1 are significantly higher in AML samples compared to healthy bone marrow samples. (E) In both the TCGA database and TARGET database, AML patients exhibiting high CTNNB1 expression show lower survival rates.

Supplementary Figure 19: USP20 Enhances AML Progression via Deubiquitination of CTNNB1.(A) Quantitative reverse transcription PCR (qRT-PCR) was employed to assess the mRNA levels of USP20 and SQSTM1 in MV4-11, kasumi-1, and CMK cells stably expressing sh-NC, sh-USP20#2, and sh-USP20#3.(B) Western blot analysis was conducted to evaluate the protein levels of USP20, CTNNB1, SQSTM1, and β-actin in MV4-11 cells treated with AS1517499.(C) Following a 6-hour incubation with 20 μM MG-132 in CMK cells stably expressing sh-NC, sh-USP20#2, and sh-USP20#3, the protein levels of USP20, CTNNB1, SQSTM1, and β-actin were determined using western blotting.(D) After treating CMK cells with AS1517499 for 6 hours alongside a 20 μM MG-132 incubation period, western blotting was utilized to detect the protein levels of USP20, CTNNB1,SQSTM1,and β-actin. (E) Statistical graphs were generated to illustrate changes in CTNNB1 protein levels following treatment with 100 μg/ml actinomycin (CHX) applied to MV4-11 and kasumi-1 cells expressing sh-NC or sh-USP20#2 or #3 over a specified duration. (F) A statistical graph was created to depict alterations in SQSTM1 protein levels after administering 100 μg/ml actinomycin (CHX) to MV4-11 and kasumi-1 cells harboring either sh-NC or sh-USP20#2 or #3 for a designated time frame. Data are presented as mean ± standard deviation (mean ± SD); no significant difference is indicated as ns; * $P < 0.05$; ** $P < 0.01$; *** $P < 0.001$; **** $P < 0.0001$. All error bars in the text represent standard deviations.

Supplementary Figure 20: Pie chart illustrating the distribution of DNA binding sites for various antibodies in CMK cells, as determined by the Cut&tag assay. (A) USP20 antibody / CMK cells. (B) CTNNB1 antibody / CMK cells. (C) ELF1 antibody / CMK cells. (D) ERG antibodies / CMK cells. (E) RUNX1 antibody / CMK cells.

Supplementary Figure 21: (A) The heatmap generated from the Cut&tag study indicates that ELF1, ERG, RUNX1, USP20, and CTNNB1 occupy common genomic loci within the CMK cell lines. (B) The distribution of binding peaks for ELF1, ERG, RUNX1, USP20, and CTNNB1 is shown within ±1000 bp intervals surrounding the USP20 binding site. (C) The IGV profile from Cut&tag reveals signals for ELF1, ERG, RUNX1, USP20, and CTNNB1 along with H3K27ac at the JAG1 gene locus. (D) PCR analysis demonstrated JAG1 mRNA expression levels following knockdown of ELF1, ERG, RUNX1, USP20 and CTNNB1. (E) The IGV map derived from Cut&tag displays signals for ELF1, ERG, RUNX1, USP20, CTNNB1, and H3K27ac at the ADAM17 gene locus. (F) PCR results indicated mRNA expression levels of ADAM17 after knockdown of ELF11, ERG, RUNXI, USP20, and CTTNB1. Data are presented as mean ± standard deviation (mean ± SD); no significant difference was observed (ns), * $P < 0.05$; ** $P < 0.01$; *** $P < 0.001$; **** $P < 0.000l$. All error bars in the text represent standard deviations.

Supplementary Figure 22: Calculating the cancer dependency map of the CCND2 gene in the DepMap project using the Chronos algorithm.

Supplementary Table 1: Enhancer subsequence information.
Supplementary Table 2: Information on antibodies used in Western blotting of proteins and Cut&tag
Supplementary Table 3: Primer Sequence Information.

Supplementary Table 4: Interfering Plasmid Sequence Information
Supplementary Table 5: Sequences of sg-NC and sgRNA.
Supplementary Table 6: ChIP-seq data of CMK cells -1
Supplementary Table 7: ChIP-seq data of CMK cells -2
Supplementary Table 8: Binding peaks identified in CMK by Cut&tag analysis of ELF1 -1
Supplementary Table 9: Binding peak-1 identified by Cut&tag analysis of ERG in CMK
Supplementary Table 10: Binding peak-1 identified in CMK by Cut&tag analysis of ETV6
Supplementary Table 11: Binding peak-1 identified by IKZF2 analysis through Cut&tag in CMK
Supplementary Table 12: Binding peak-1 identified by RUNX1 through Cut&tag analysis in CMK
Supplementary Table 13: Binding peak-1 identified in CMK by Cut&tag analysis of TAL-1
Supplementary Table 14: Binding peak-1 identified by Cut&tag analysis of ZNF217 in CMK
Supplementary Table 15: List of inhibitors for virtual screening and their IC50
Supplementary Table 16: Results of Gene set enrichment analysis (GSEA) after USP20 knockout in CMK cells.
Supplementary Table 17: Results from mass spectrometry and software analysis.
Supplementary Table 18: Binding peak-1 detected in CMK cells by Cut&tag analysis of USP20.
Supplementary Table 19: Binding peak-1 detected in CMK cells by Cut&tag analysis of USP20.
Supplementary Table 20: Binding peak-1 detected in CTNNB1 by Cut&tag analysis in CMK
Supplementary Table 21: Binding peak-2 detected in CTNNB1 in CMK cells by Cut&tag analysis.
