# Supplementary material for: USP20, a Super-enhancer Regulated Gene, Promotes Acute Myeloid Leukemia Progression through CTNNB1 Deubiquitination
Source: Int J Biol Sci. 2026 Feb 11;22(5):2665–86. doi: 10.7150/ijbs.122898 (PMC12965243; doi:10.7150/ijbs.122898)
Supplement: Supplementary file 2 — Supplementary figures. [file ijbsv22p2665s2.zip › 附图/Supplementary Figure15.pdf]

A

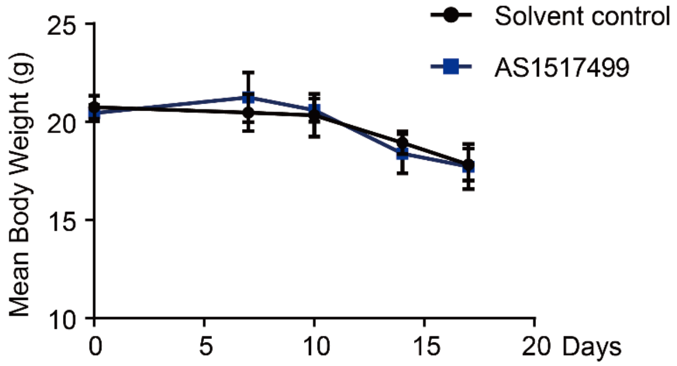

B

Abbreviation	Unit	Solvent control-1	Solvent control-2	AS1517499 -1	AS1517499 -2	Reference Range
WBC	10^9/L	1.5	1.6	1.3	2.1	0.8-10.6
Lymph#	10^9/L	1	0.6	0.6	0.5	0.6-8.9
Mon#	10^9/L	0	0.1	0	0.1	0.04-1.4
Gran#	10^9/L	0.5	0.9	0.7	1.5	0.23-3.6
Lymph%	%	64.4	40.1	43.1	24.1	40-92
Mon%	%	5.1	8.3	6.7	5.9	0.9-18
Gran%	%	30.5	51.6	50.2	70	6.5-50
RBC	10^12/L	8.06	7.42	7.38	7.52	6.5-11.5
HGB	g/L	168	157	150	155	110-165
HCT	%	45.7	40.2	40	40.9	35-55
MCV	fL	56.8	54.3	54.3	54.5	41-55
MCH	pg	20.8	21.1	20.3	20.6	13-18
MCHC	g/L	367	390	375	378	300-360
RDW	%	19.9	15.7	14.8	14.5	12-19
PLT	10^9/L	935	1206	1006	1276	400-1600
MPV	fL	4.6	5.2	5.8	5.9	4.0-6.2
PDW		16.8	16.8	17.7	17.2	12.0-17.5
PCT	%	0.43	0.627	0.583	.***	0.100-0.780

C

SampleId	Unit	Solvent control-1	Solvent control-2	AS1517499 -1	AS1517499 -2	Reference Range
ALT	U/L	14	14	13	13	10.06-96.47
AST	U/L	38	36	30	28	36.31-235.48
ALP	U/L	146	147	159	133	22.52-474.35
γ-GGT	U/L	1	1	1	1.2	0-7.78
TP	g/L	40.8	43.5	42.1	41.5	38.02-75.06
ALB	g/L	59.3	63.1	60.3	58.9	21.22-39.15
TB	umol/L	5.62	9.17	8.18	9.26	6.09-53.06
DB	umol/L	2.84	4.47	4.41	4.5	0.45-33.89
UREA	mmol/L	9.98	8.91	9.56	8.84	10.81-34.74
CREA	umol/L	14.948	12.252	15.11	16.474	10.91-85.09
UA	umol/L	63	59	76	76	44.42-224.77
