# Supplementary material for: USP20, a Super-enhancer Regulated Gene, Promotes Acute Myeloid Leukemia Progression through CTNNB1 Deubiquitination
Source: Int J Biol Sci. 2026 Feb 11;22(5):2665–86. doi: 10.7150/ijbs.122898 (PMC12965243; doi:10.7150/ijbs.122898)
Supplement: Supplementary file 2 — Supplementary figures. [file ijbsv22p2665s2.zip › 附图/Supplementary figure20.pdf]

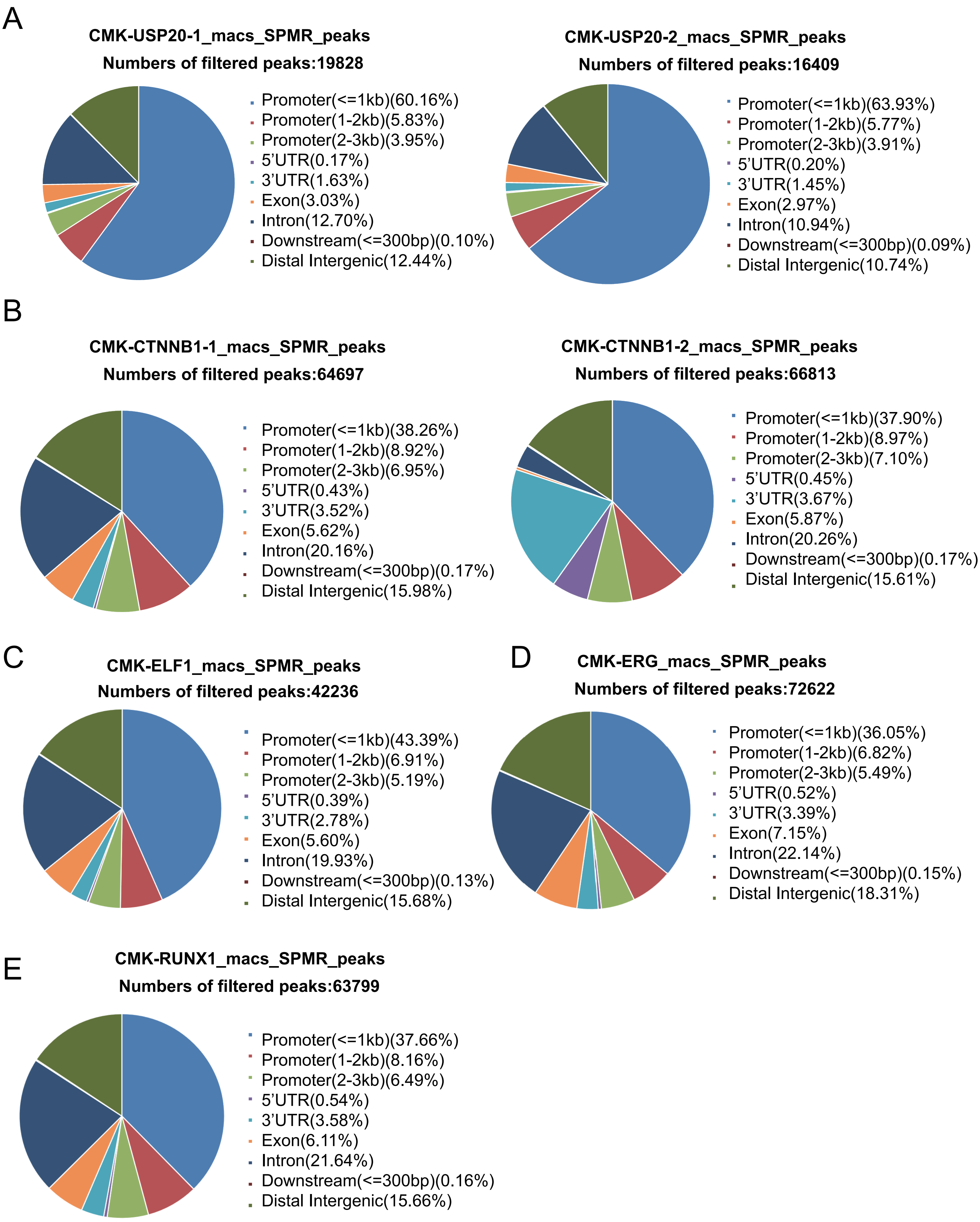
A
CMK-USP20-1_macs_SPMR_peaks
Numbers of filtered peaks:19828
Promoter(<=1kb)(60.16%)
Promoter(1-2kb)(5.83%)
Promoter(2-3kb)(3.95%)
5’UTR(0.17%)
3’UTR(1.63%)
Exon(3.03%)
Intron(12.70%)
Downstream(<=300bp)(0.10%)
Distal Intergenic(12.44%)
CMK-USP20-2_macs_SPMR_peaks
Numbers of filtered peaks:16409
Promoter(<=1kb)(63.93%)
Promoter(1-2kb)(5.77%)
Promoter(2-3kb)(3.91%)
5’UTR(0.20%)
3’UTR(1.45%)
Exon(2.97%)
Intron(10.94%)
Downstream(<=300bp)(0.09%)
Distal Intergenic(10.74%)
B
CMK-CTNNB1-1_macs_SPMR_peaks
Numbers of filtered peaks:64697
Promoter(<=1kb)(38.26%)
Promoter(1-2kb)(8.92%)
Promoter(2-3kb)(6.95%)
5’UTR(0.43%)
3’UTR(3.52%)
Exon(5.62%)
Intron(20.16%)
Downstream(<=300bp)(0.17%)
Distal Intergenic(15.98%)
CMK-CTNNB1-2_macs_SPMR_peaks
Numbers of filtered peaks:66813
Promoter(<=1kb)(37.90%)
Promoter(1-2kb)(8.97%)
Promoter(2-3kb)(7.10%)
5’UTR(0.45%)
3’UTR(3.67%)
Exon(5.87%)
Intron(20.26%)
Downstream(<=300bp)(0.17%)
Distal Intergenic(15.61%)
C
CMK-ELF1_macs_SPMR_peaks
Numbers of filtered peaks:42236
Promoter(<=1kb)(43.39%)
Promoter(1-2kb)(6.91%)
Promoter(2-3kb)(5.19%)
5’UTR(0.39%)
3’UTR(2.78%)
Exon(5.60%)
Intron(19.93%)
Downstream(<=300bp)(0.13%)
Distal Intergenic(15.68%)
D
CMK-ERG_macs_SPMR_peaks
Numbers of filtered peaks:72622
Promoter(<=1kb)(36.05%)
Promoter(1-2kb)(6.82%)
Promoter(2-3kb)(5.49%)
5’UTR(0.52%)
3’UTR(3.39%)
Exon(7.15%)
Intron(22.14%)
Downstream(<=300bp)(0.15%)
Distal Intergenic(18.31%)
E
CMK-RUNX1_macs_SPMR_peaks
Numbers of filtered peaks:63799
Promoter(<=1kb)(37.66%)
Promoter(1-2kb)(8.16%)
Promoter(2-3kb)(6.49%)
5’UTR(0.54%)
3’UTR(3.58%)
Exon(6.11%)
Intron(21.64%)
Downstream(<=300bp)(0.16%)
Distal Intergenic(15.66%)
