# Supplementary material for: USP20, a Super-enhancer Regulated Gene, Promotes Acute Myeloid Leukemia Progression through CTNNB1 Deubiquitination
Source: Int J Biol Sci. 2026 Feb 11;22(5):2665–86. doi: 10.7150/ijbs.122898 (PMC12965243; doi:10.7150/ijbs.122898)
Supplement: Supplementary file 2 — Supplementary figures. [file ijbsv22p2665s2.zip › 附图/Supplementary Figure22.pdf]

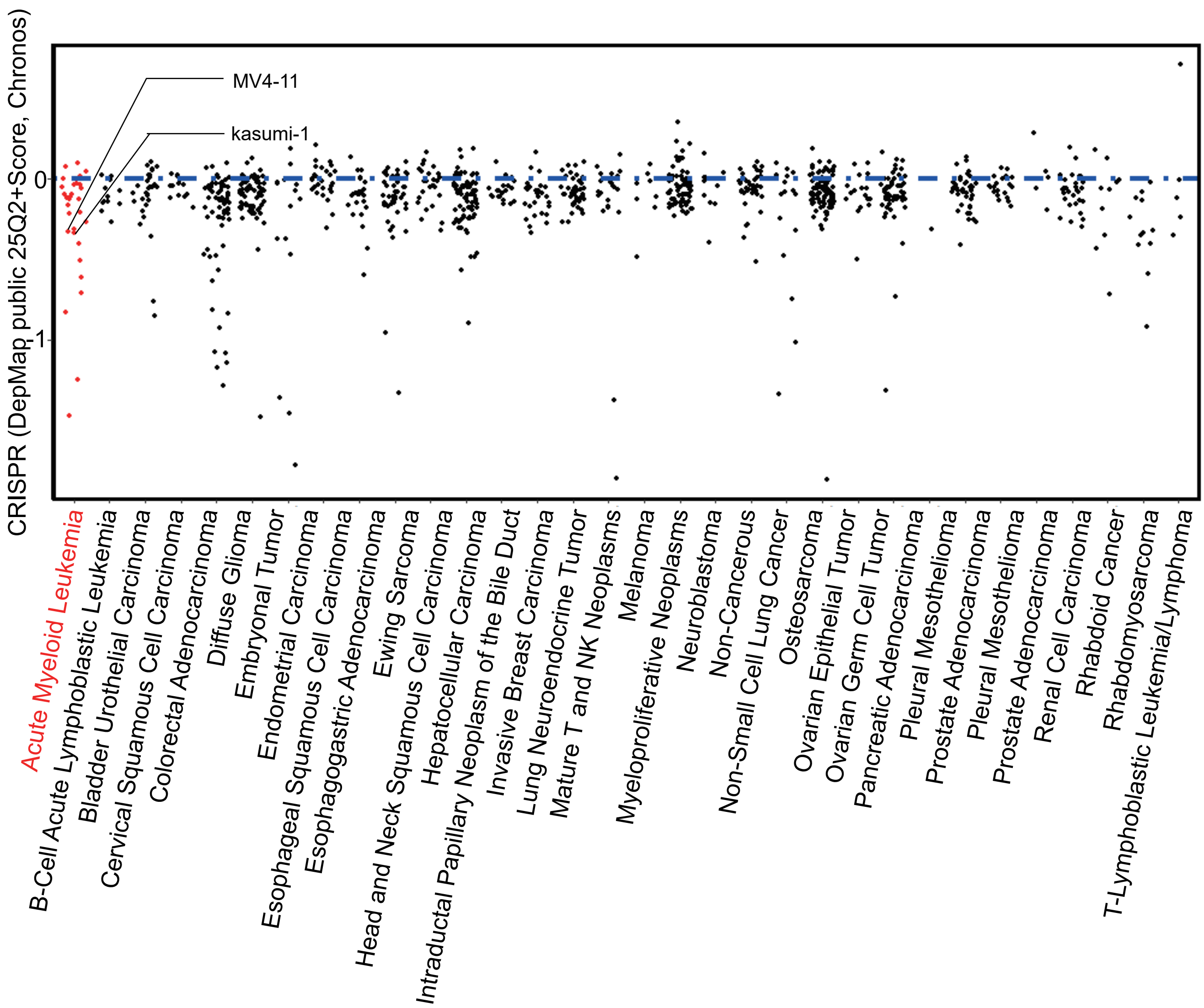

CRISPR (DepMap public 25Q2+Score, Chronos)
0
-1
MV4-11
kasumi-1
Acute Myeloid Leukemia
B-Cell Acute Lymphoblastic Leukemia
Bladder Urothelial Carcinoma
Cervical Squamous Cell Carcinoma
Colorectal Adenocarcinoma
Diffuse Glioma
Embryonal Tumor
Endometrial Carcinoma
Esophageal Squamous Cell Carcinoma
Esophagogastric Adenocarcinoma
Ewing Sarcoma
Head and Neck Squamous Cell Carcinoma
Hepatocellular Carcinoma
Intraductal Papillary Neoplasm of the Bile Duct
Invasive Breast Carcinoma
Lung Neuroendocrine Tumor
Mature T and NK Neoplasms
Melanoma
Myeloproliferative Neoplasms
Neuroblastoma
Non-Cancerous
Non-Small Cell Lung Cancer
Osteosarcoma
Ovarian Epithelial Tumor
Ovarian Germ Cell Tumor
Pancreatic Adenocarcinoma
Pleural Mesothelioma
Prostate Adenocarcinoma
Pleural Mesothelioma
Prostate Adenocarcinoma
Renal Cell Carcinoma
Rhabdoid Cancer
Rhabdomyosarcoma
T-Lymphoblastic Leukemia/Lymphoma
