# Supplementary figures and images for: USP20, a Super-enhancer Regulated Gene, Promotes Acute Myeloid Leukemia Progression through CTNNB1 Deubiquitination
Source: Int J Biol Sci. 2026 Feb 11;22(5):2665–86. doi: 10.7150/ijbs.122898 (PMC12965243; doi:10.7150/ijbs.122898)

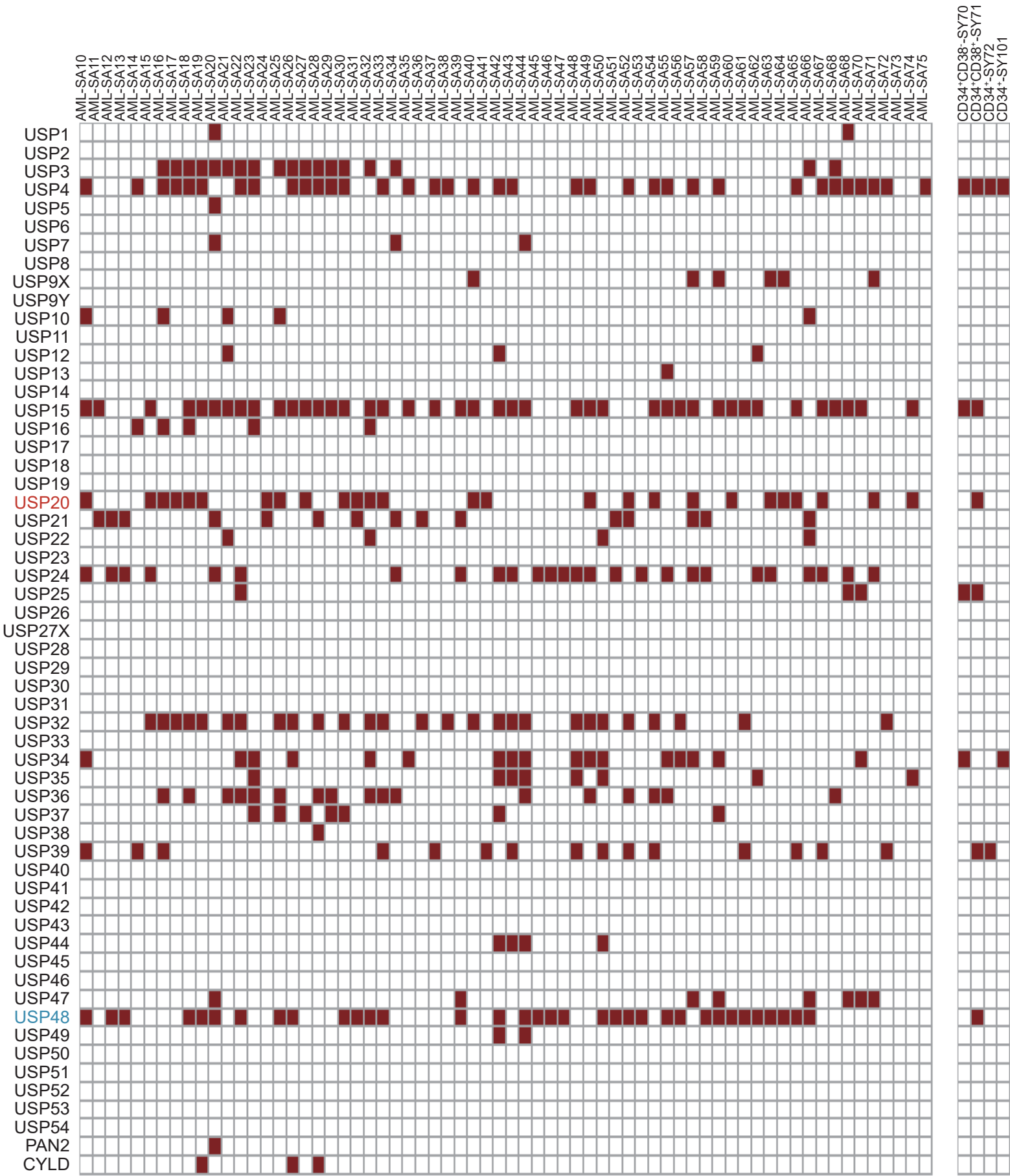

Supplement: Supplementary file 2 — Supplementary figures. [file ijbsv22p2665s2.zip › 附图/Supplementary Figure1.pdf]

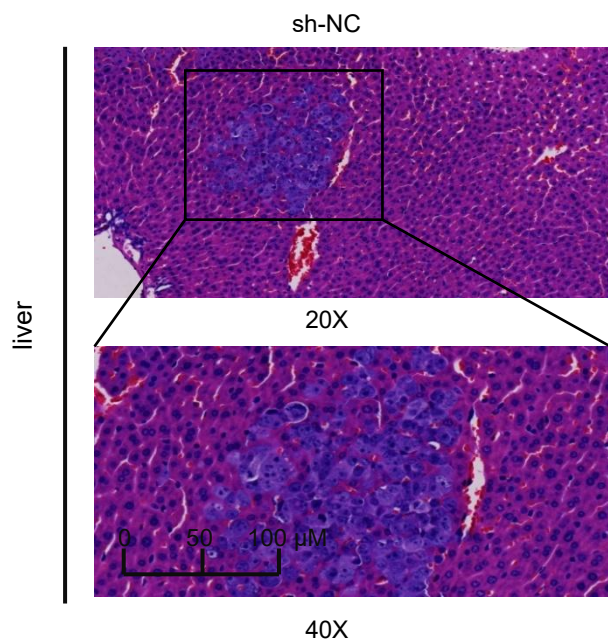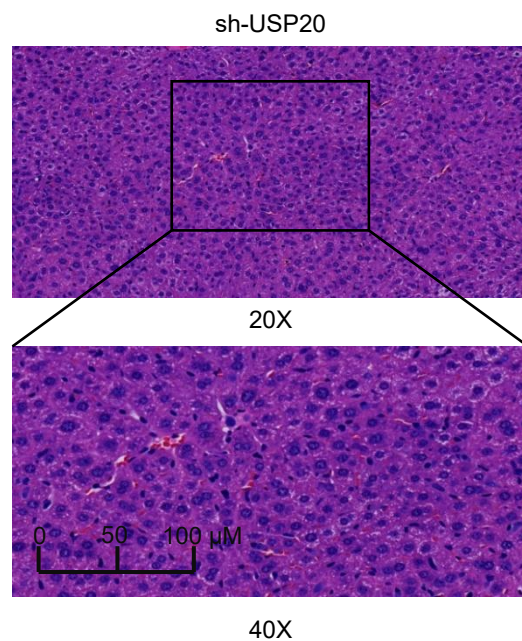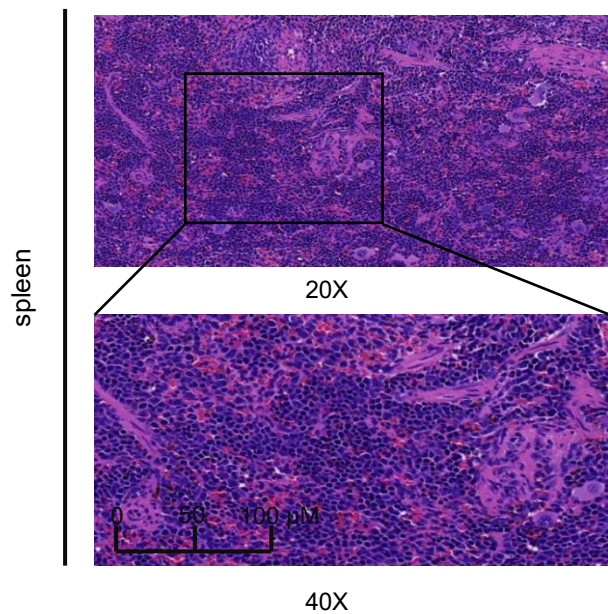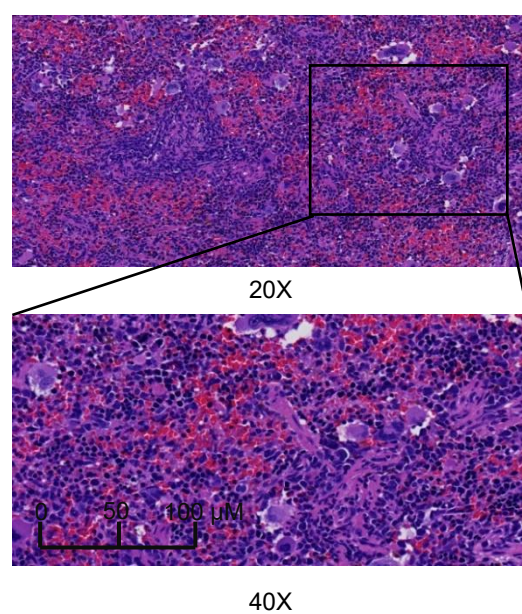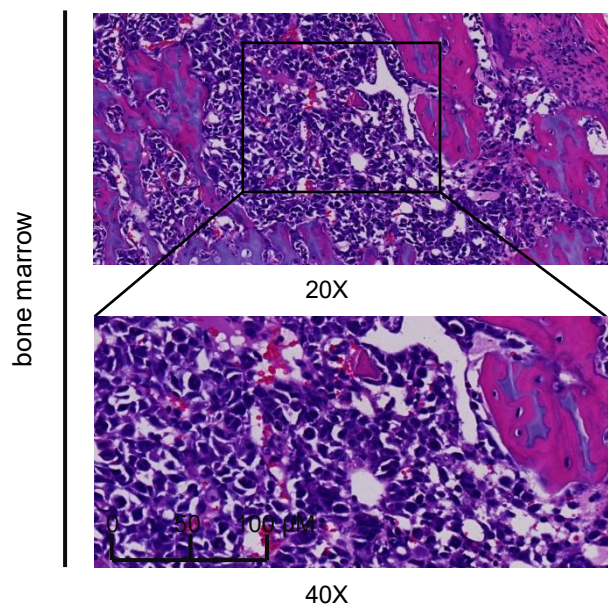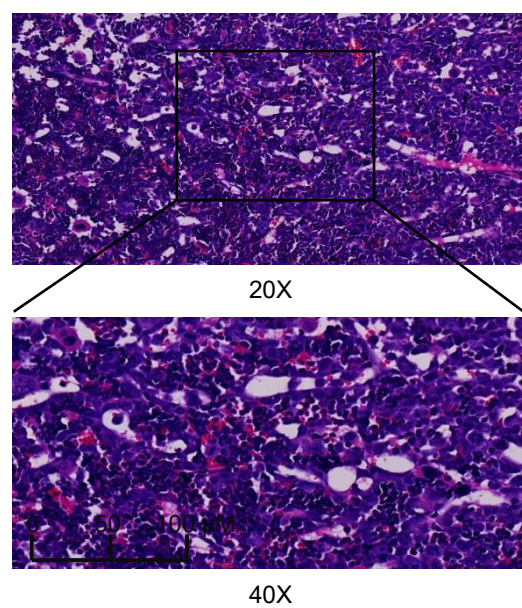

Supplement: Supplementary file 2 — Supplementary figures. [file ijbsv22p2665s2.zip › 附图/Supplementary Figure10.pdf]

A

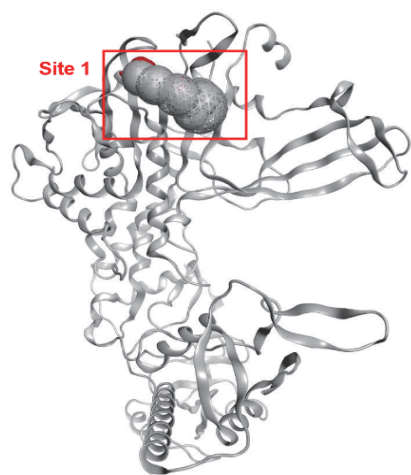

B

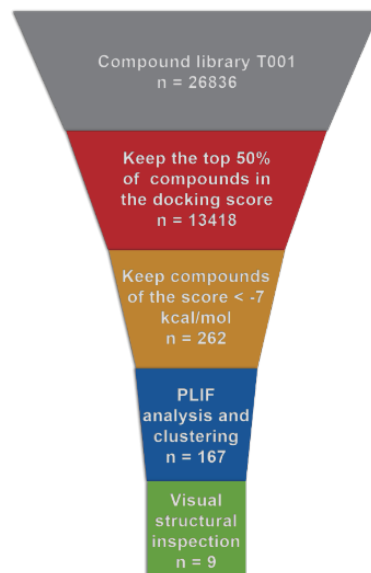

C

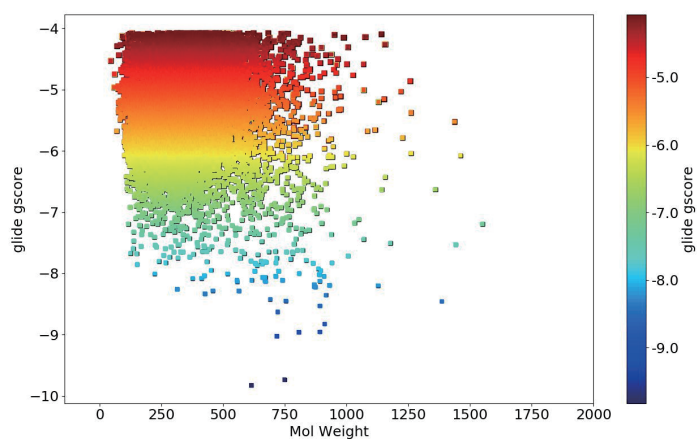

D

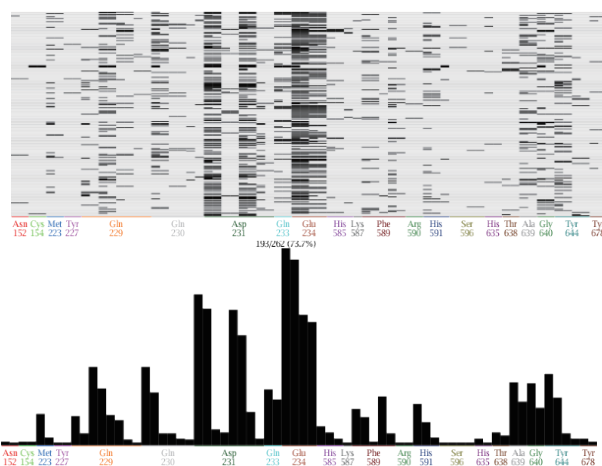

E

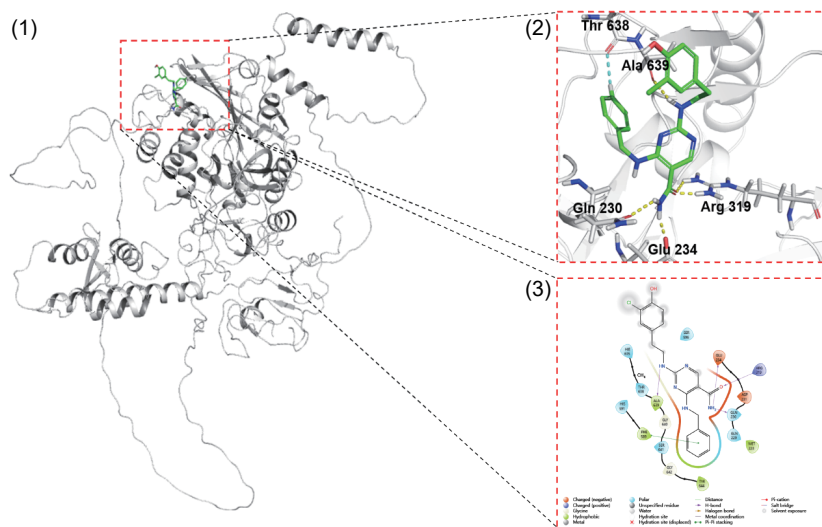

F

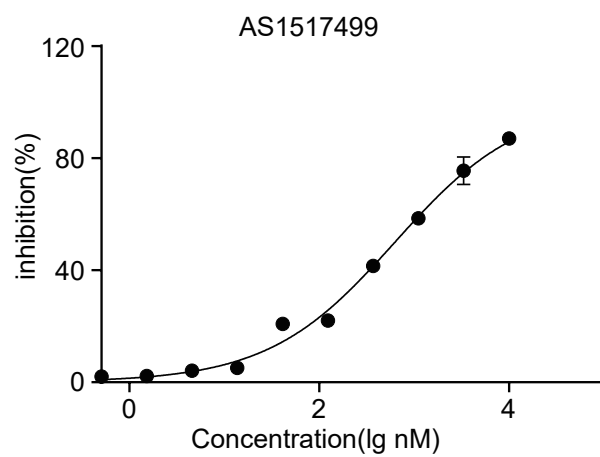

Supplement: Supplementary file 2 — Supplementary figures. [file ijbsv22p2665s2.zip › 附图/Supplementary Figure11.pdf]

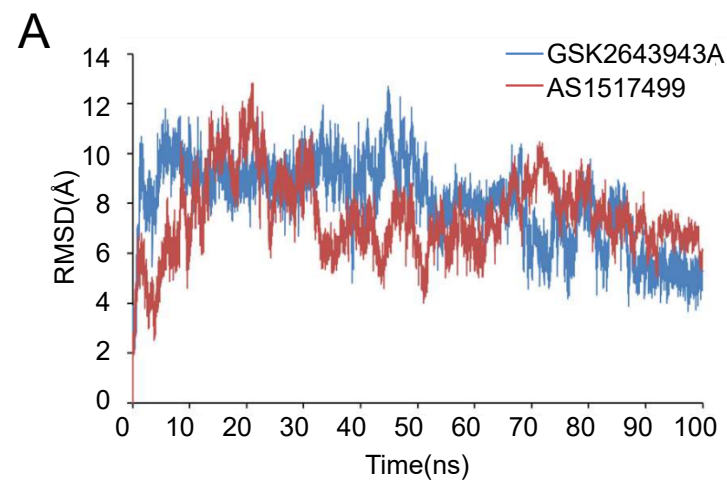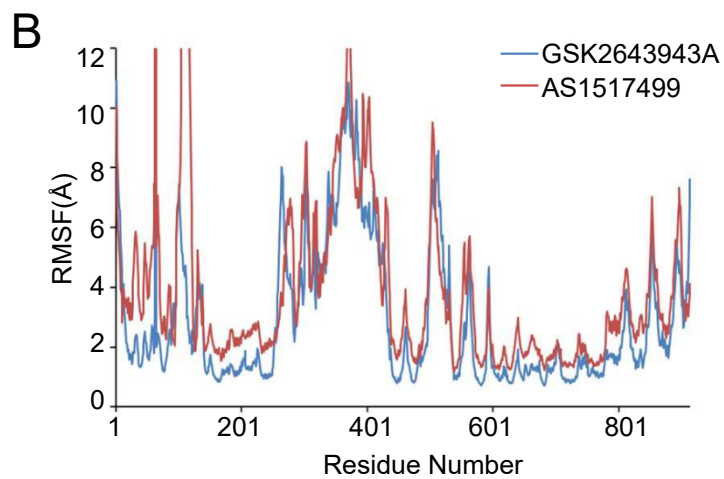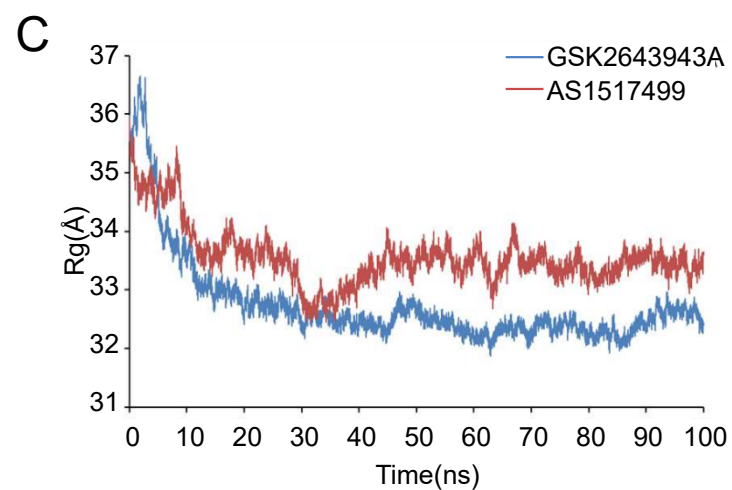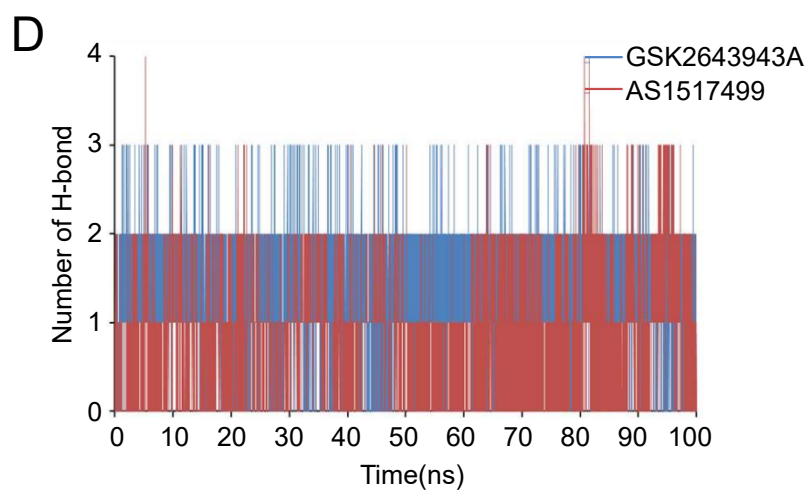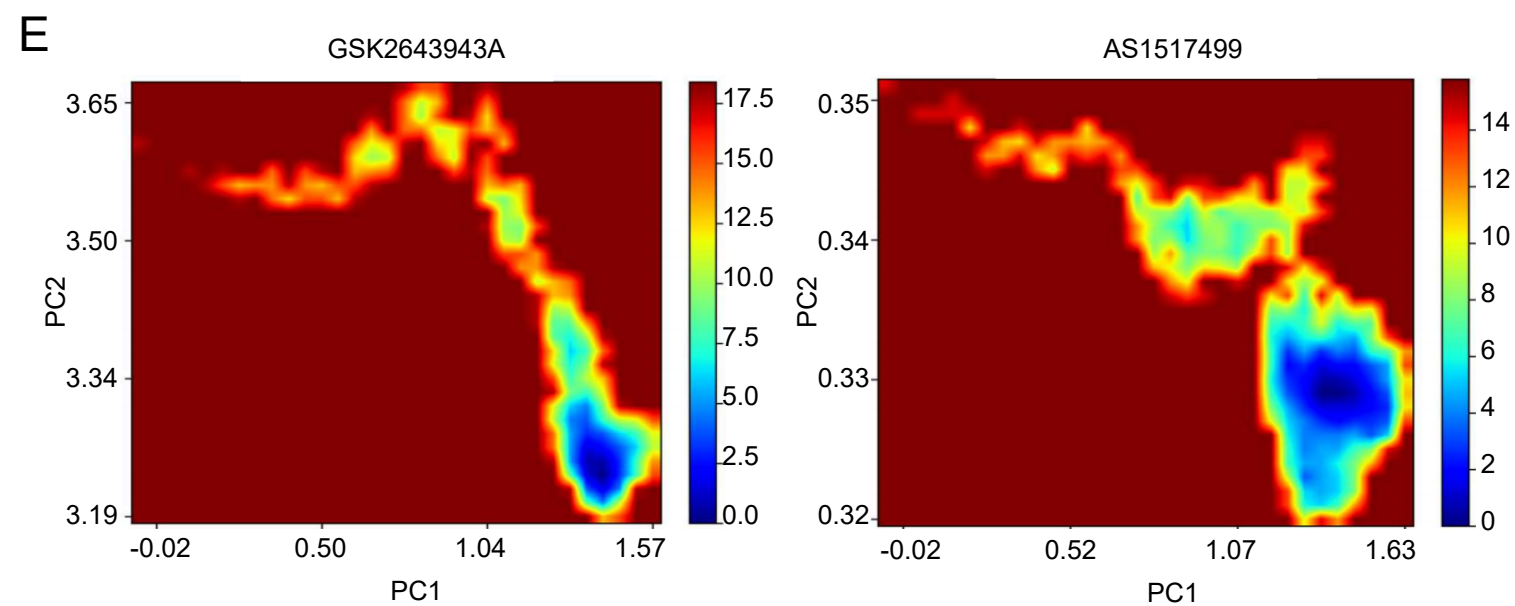

Supplement: Supplementary file 2 — Supplementary figures. [file ijbsv22p2665s2.zip › 附图/Supplementary Figure12.pdf]

A

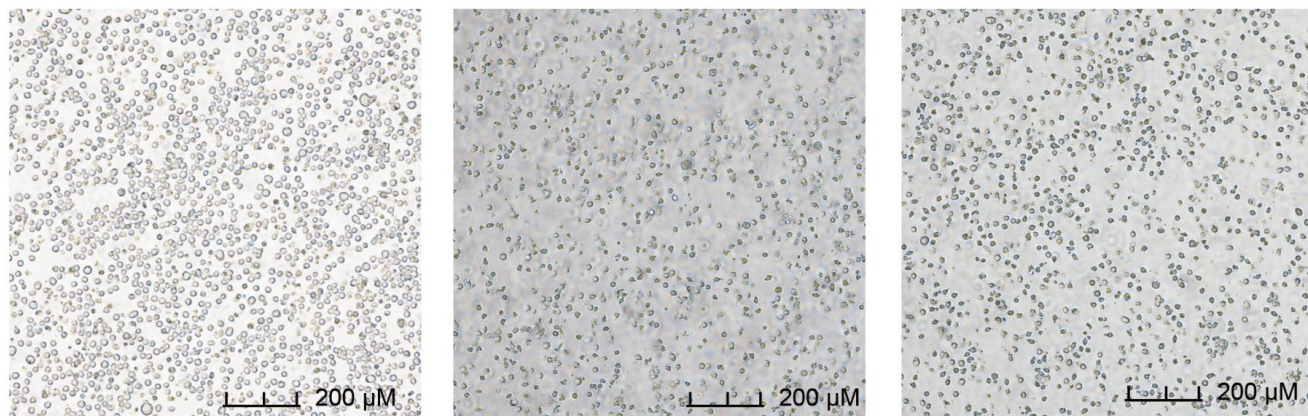

B

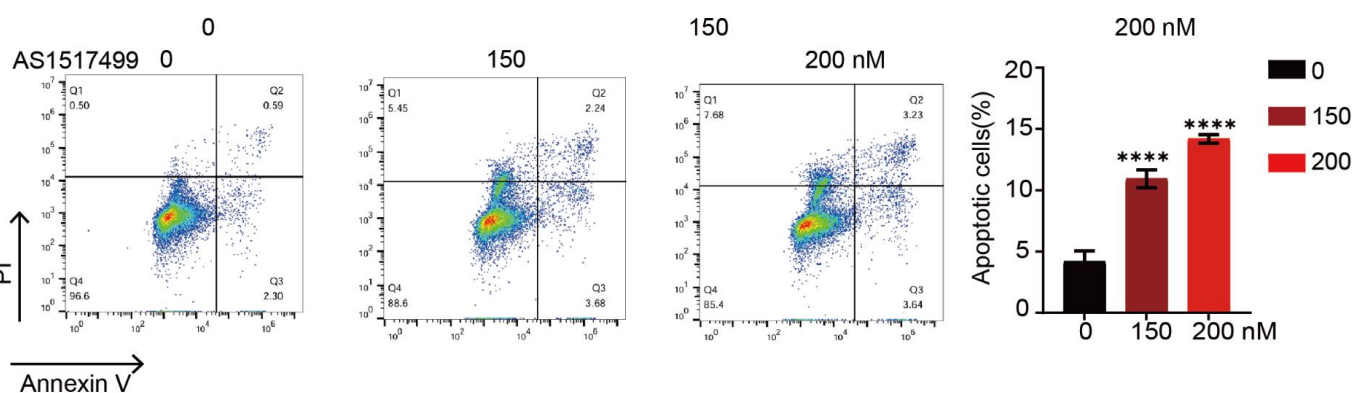

C

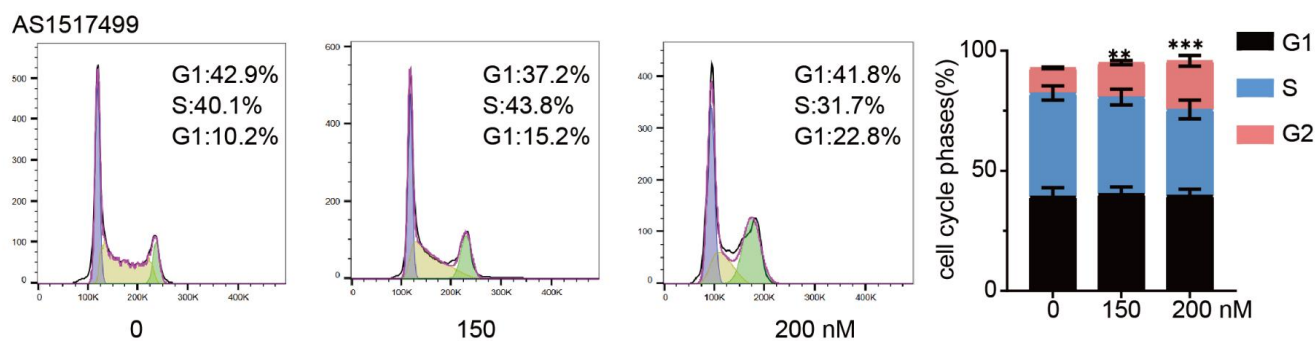

D

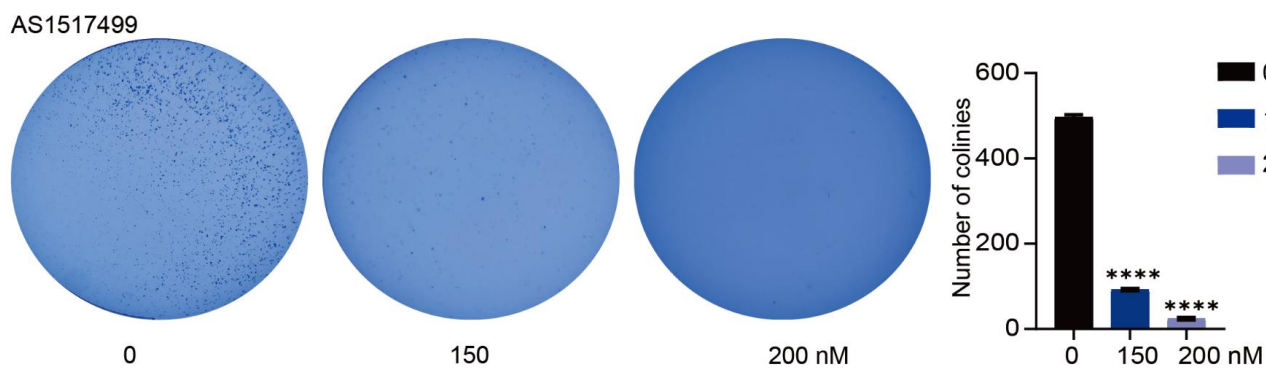

Supplement: Supplementary file 2 — Supplementary figures. [file ijbsv22p2665s2.zip › 附图/Supplementary Figure13.pdf]

**A**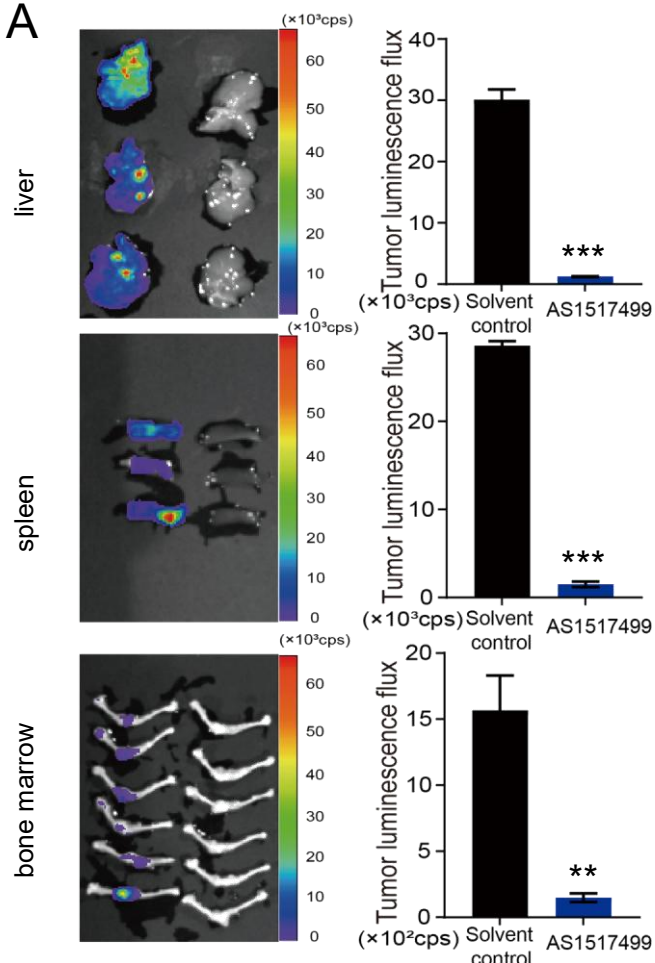**B**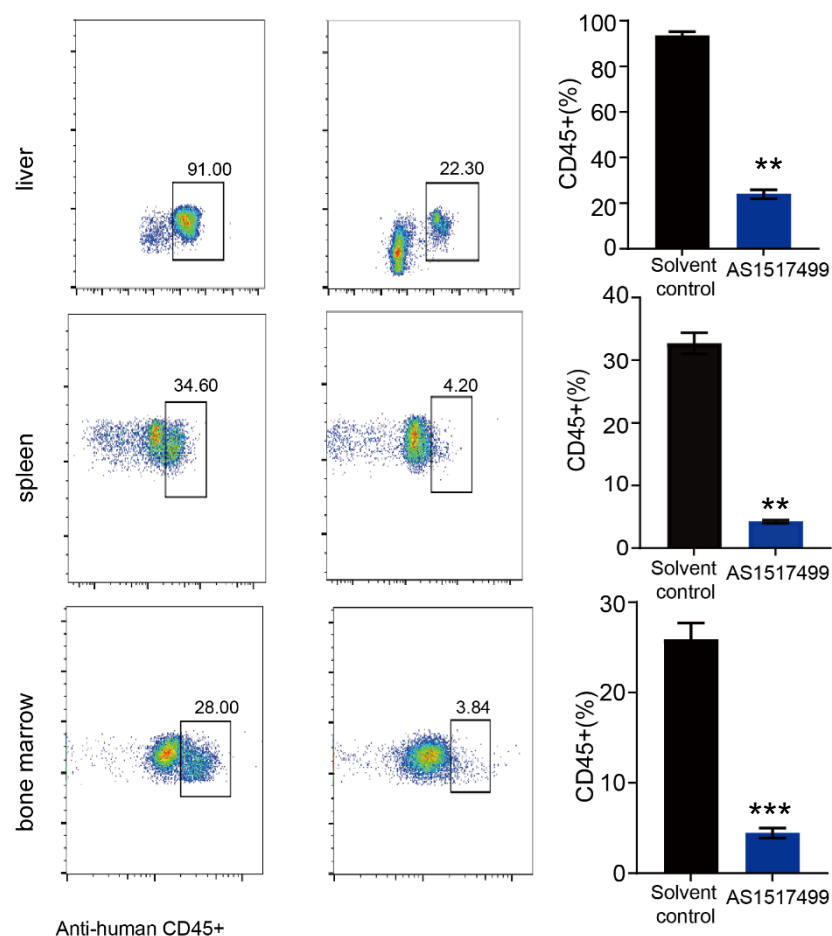**C**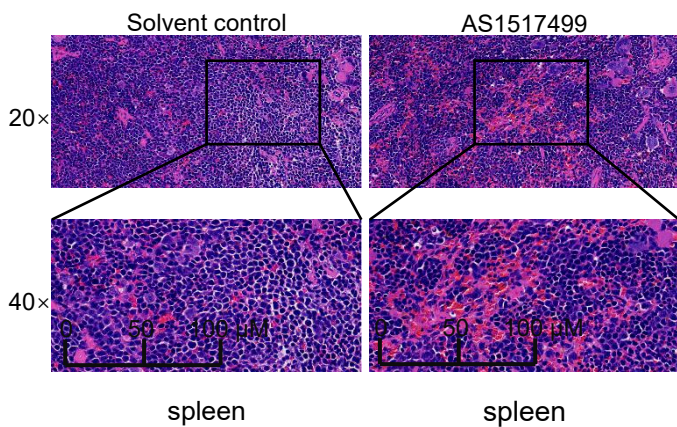**D**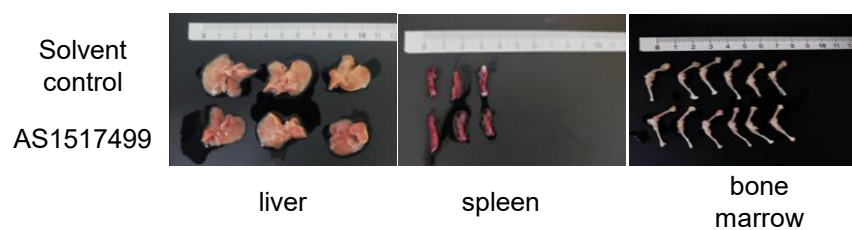**E**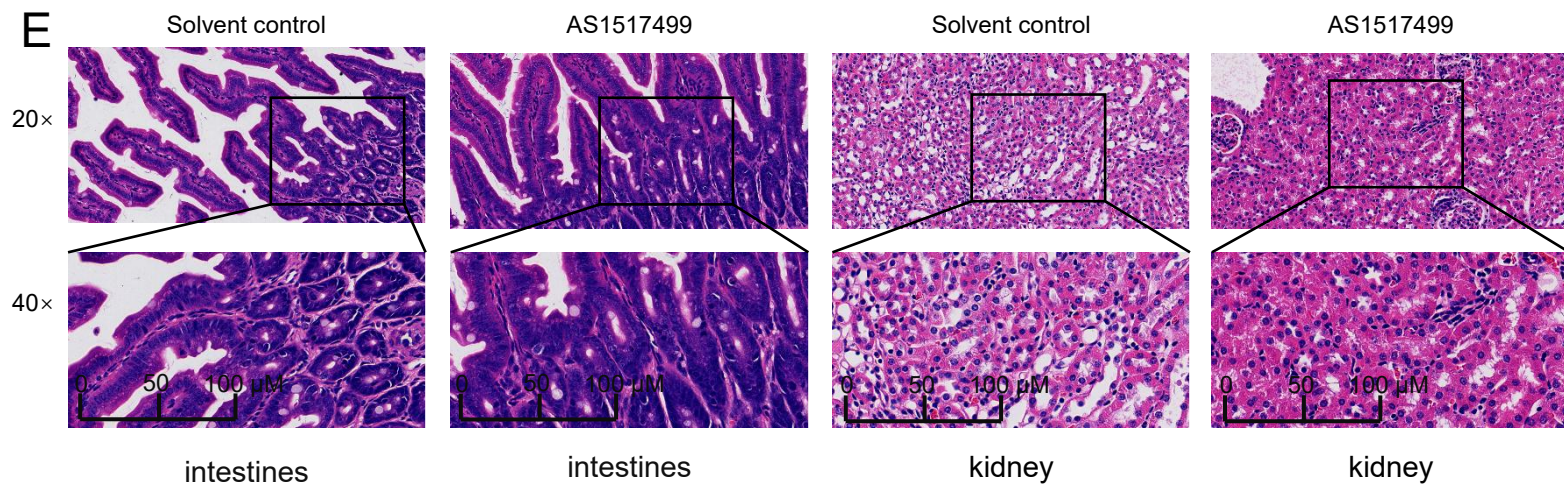

Supplement: Supplementary file 2 — Supplementary figures. [file ijbsv22p2665s2.zip › 附图/Supplementary Figure14.pdf]

Solvent control

AS1517499

liver

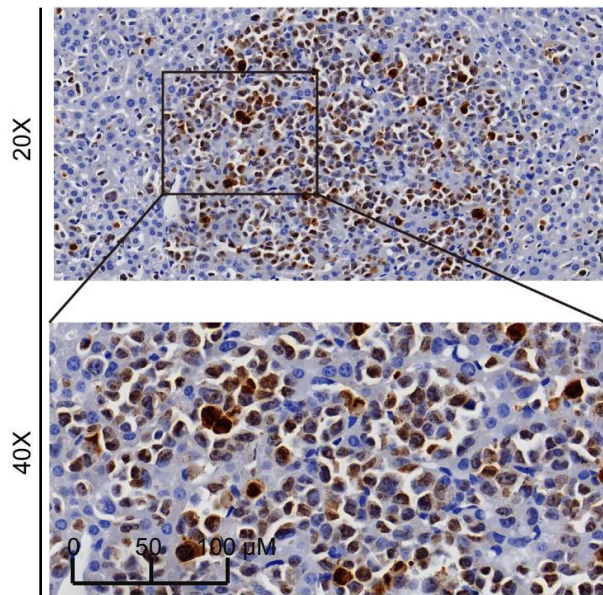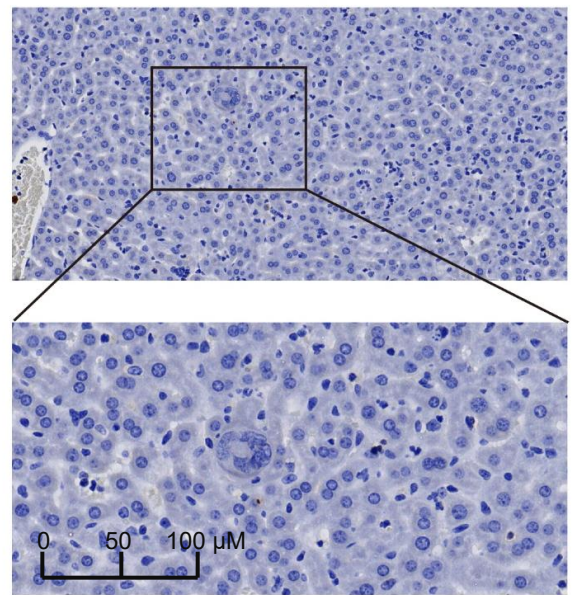

spleen

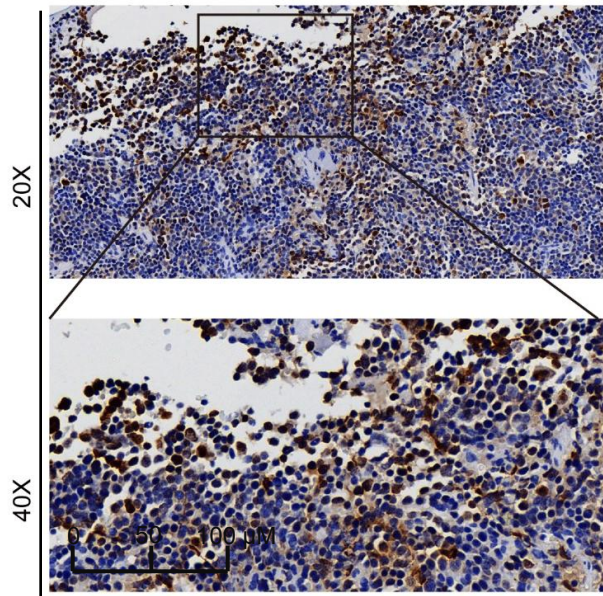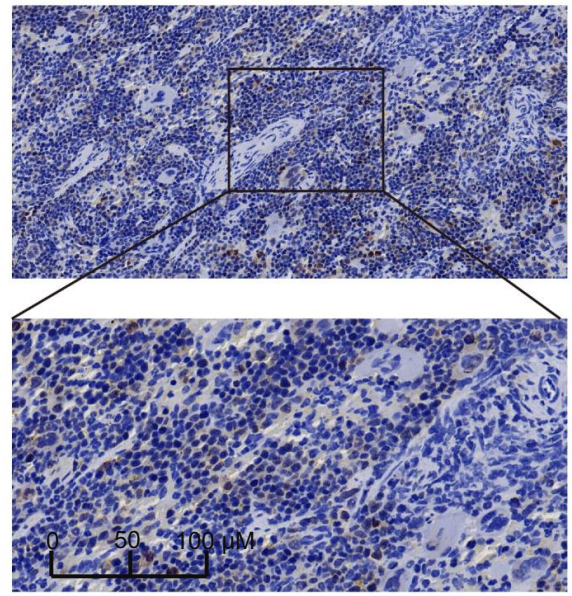

bone marrow

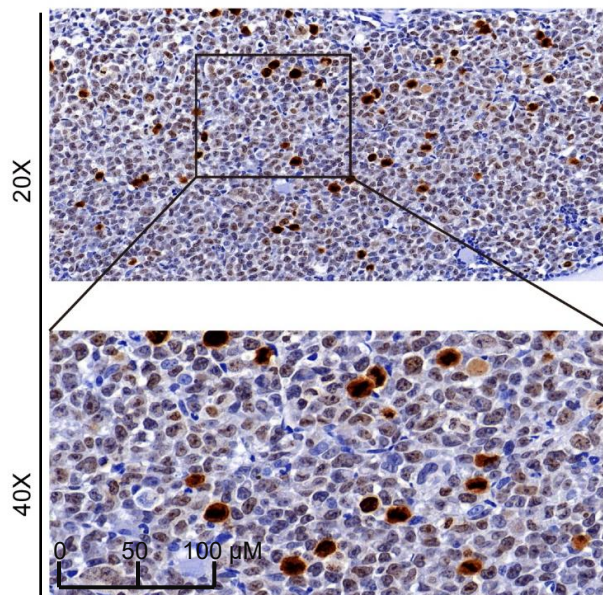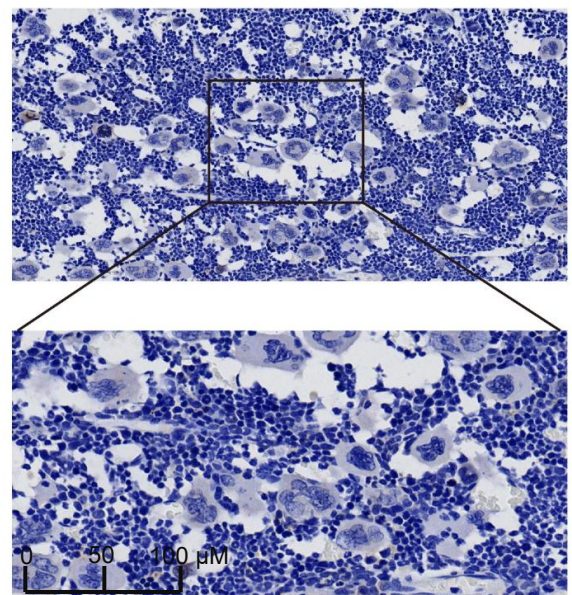

Supplement: Supplementary file 2 — Supplementary figures. [file ijbsv22p2665s2.zip › 附图/Supplementary Figure16.pdf]

A

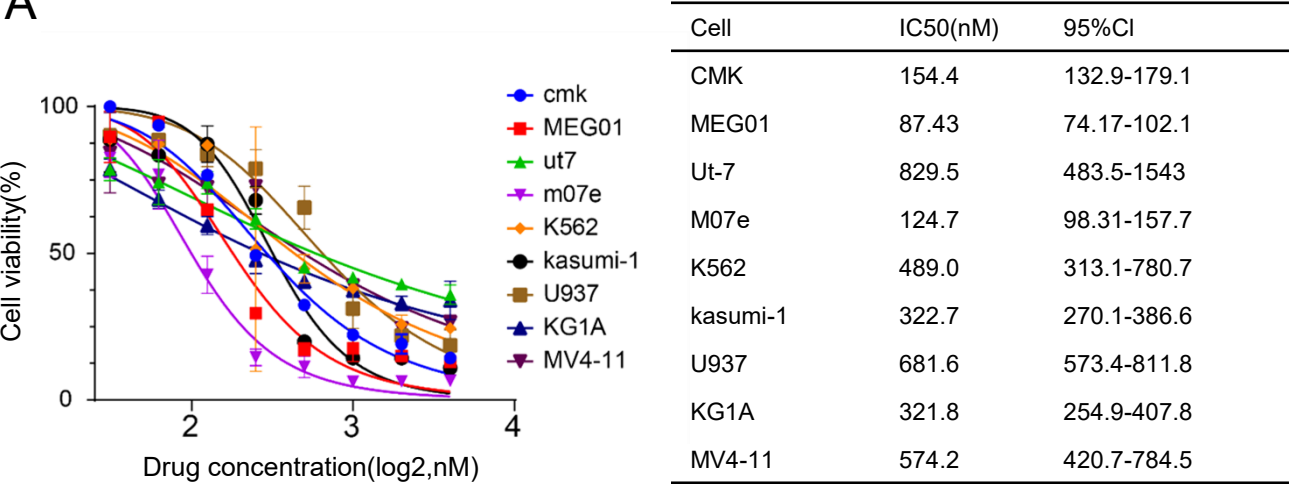

B

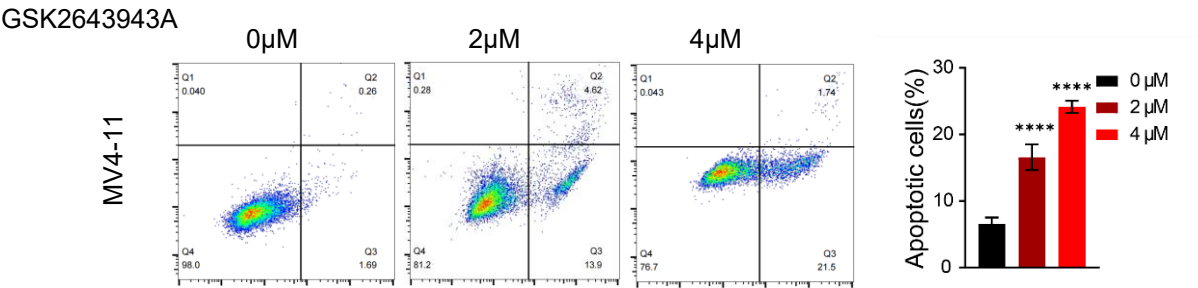

C

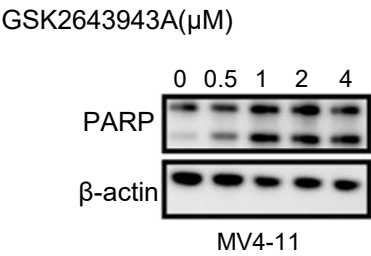

Supplement: Supplementary file 2 — Supplementary figures. [file ijbsv22p2665s2.zip › 附图/Supplementary Figure17.pdf]

A

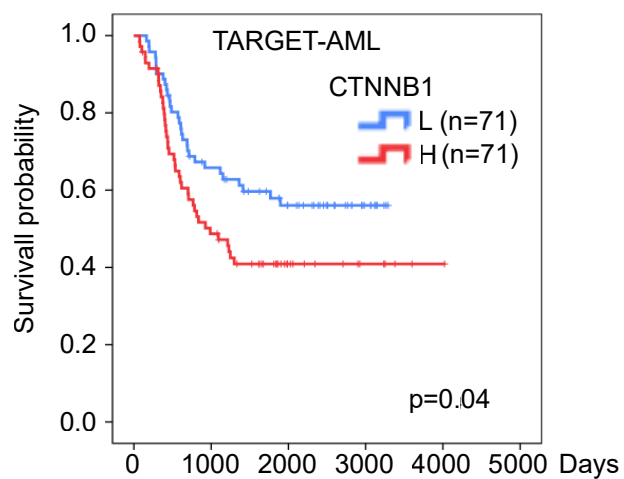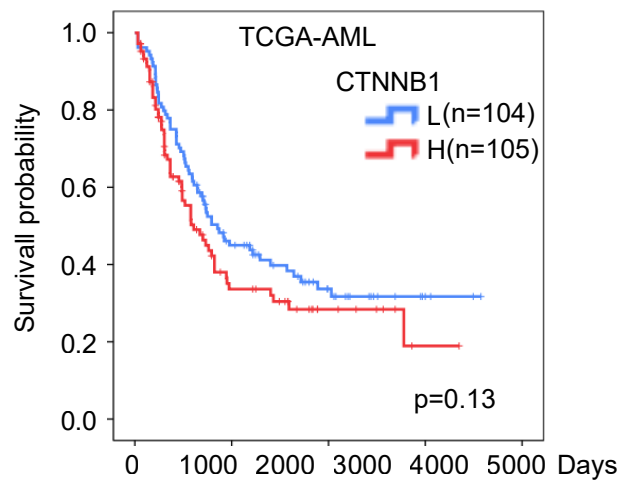

B

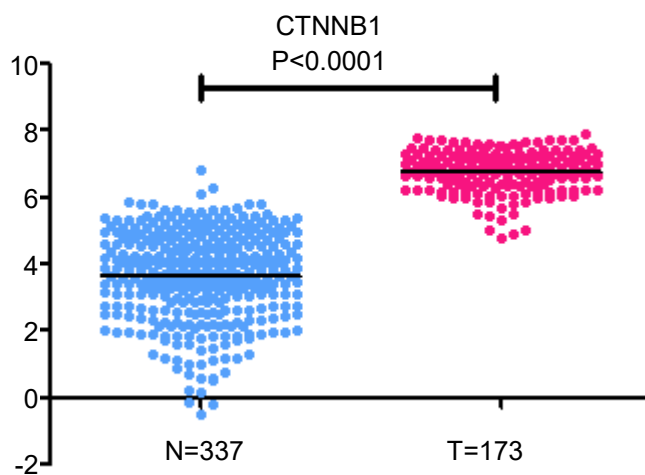

Supplement: Supplementary file 2 — Supplementary figures. [file ijbsv22p2665s2.zip › 附图/Supplementary Figure18.pdf]

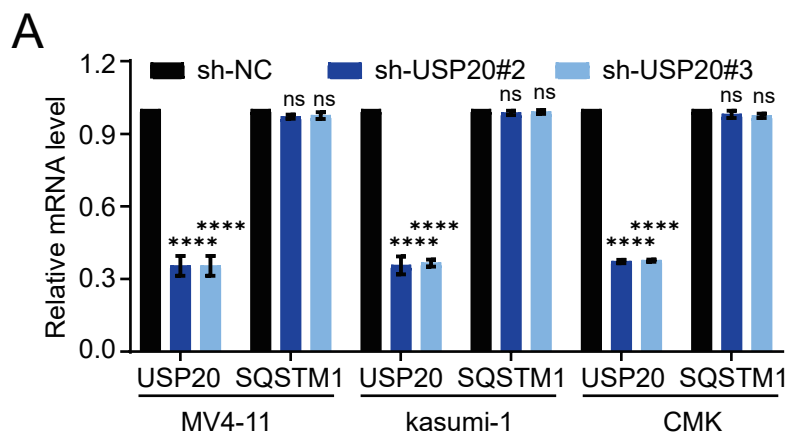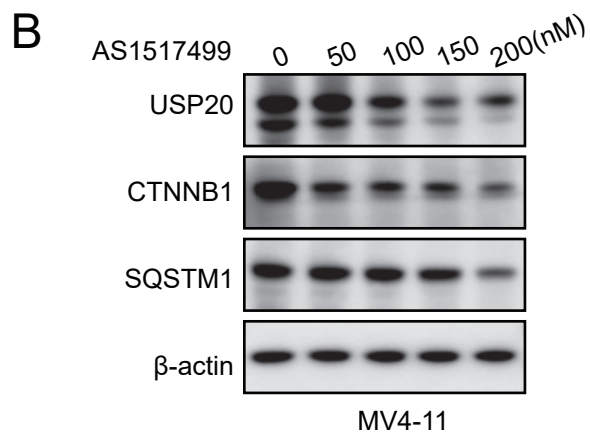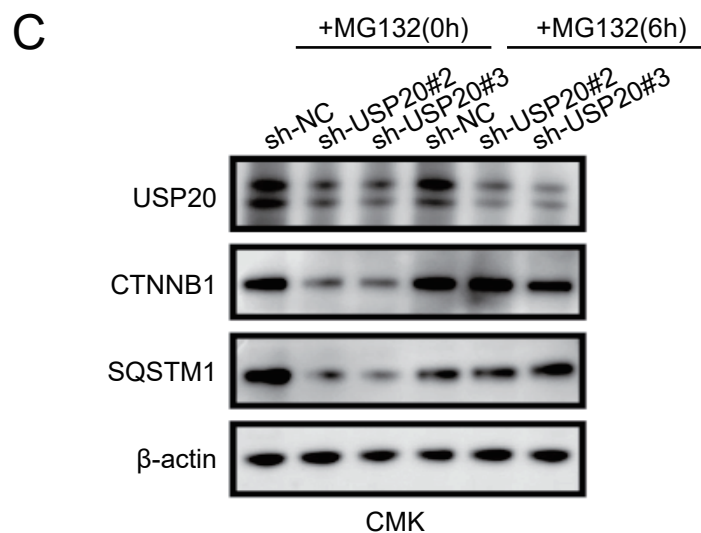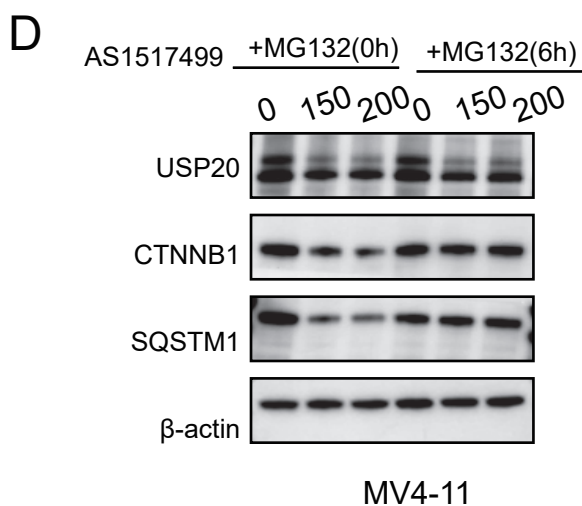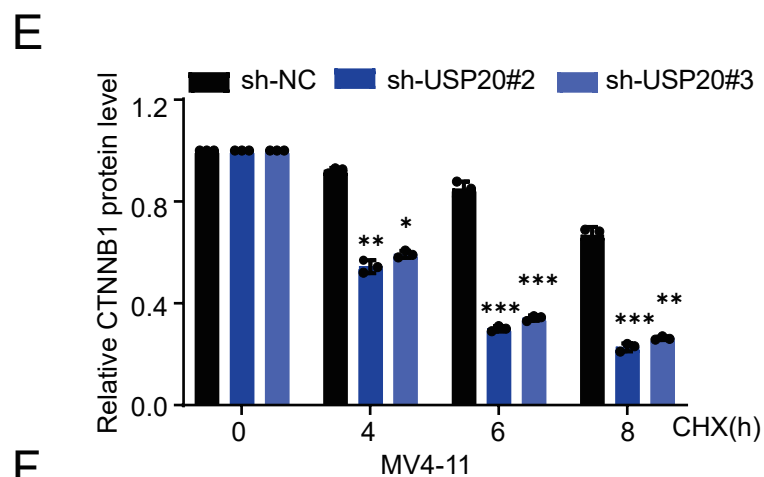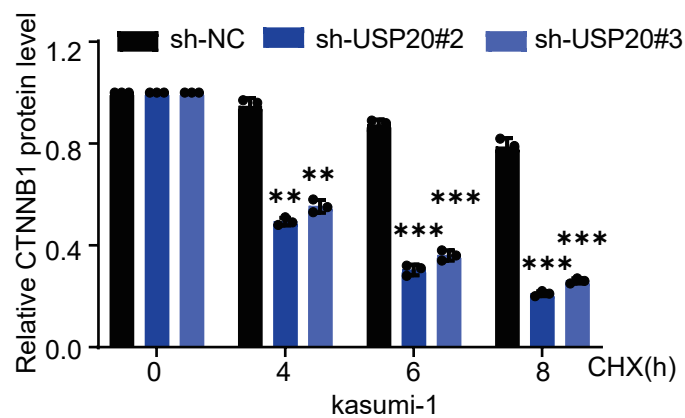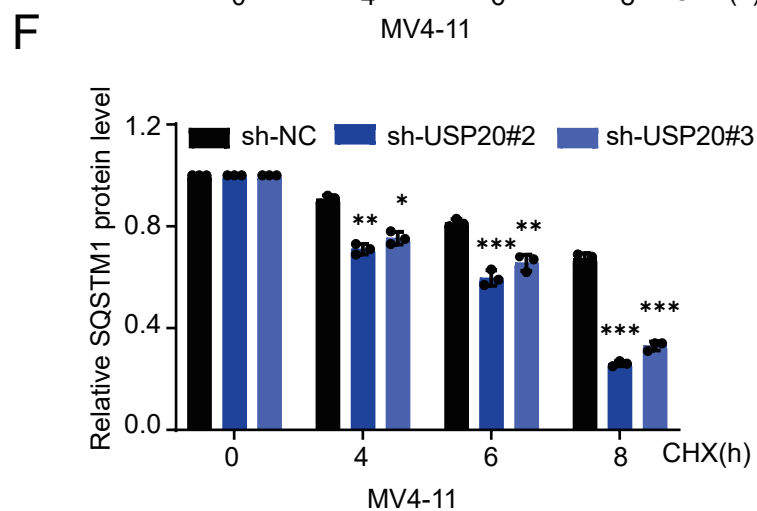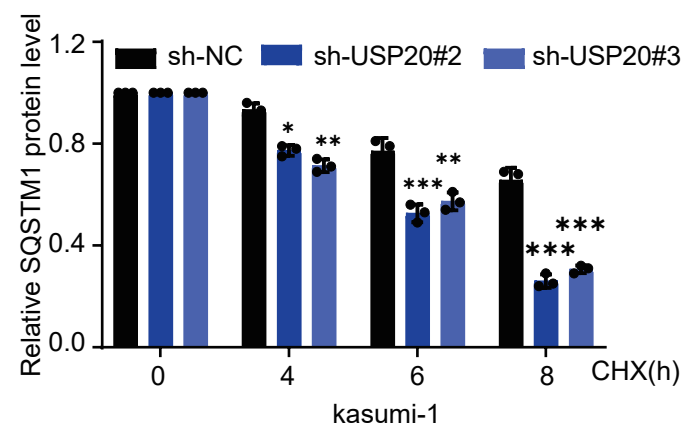

Supplement: Supplementary file 2 — Supplementary figures. [file ijbsv22p2665s2.zip › 附图/supplementary Figure19.pdf]

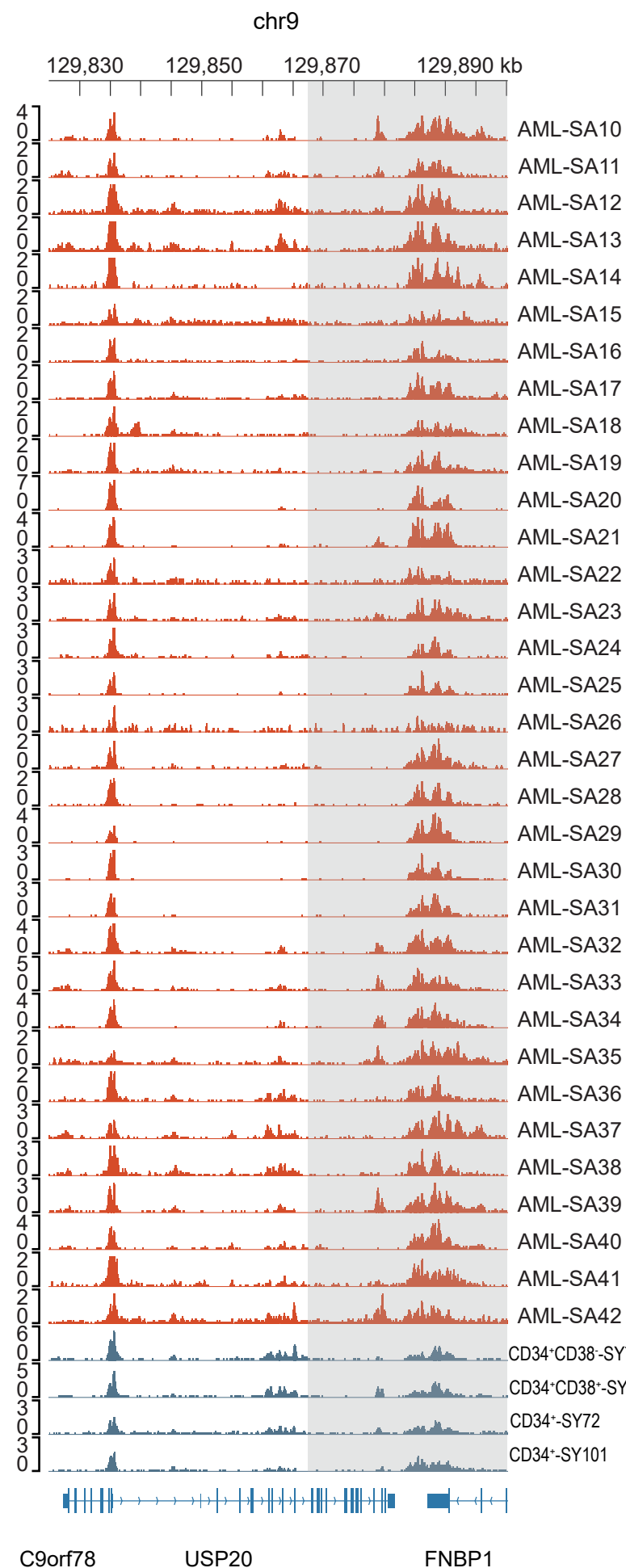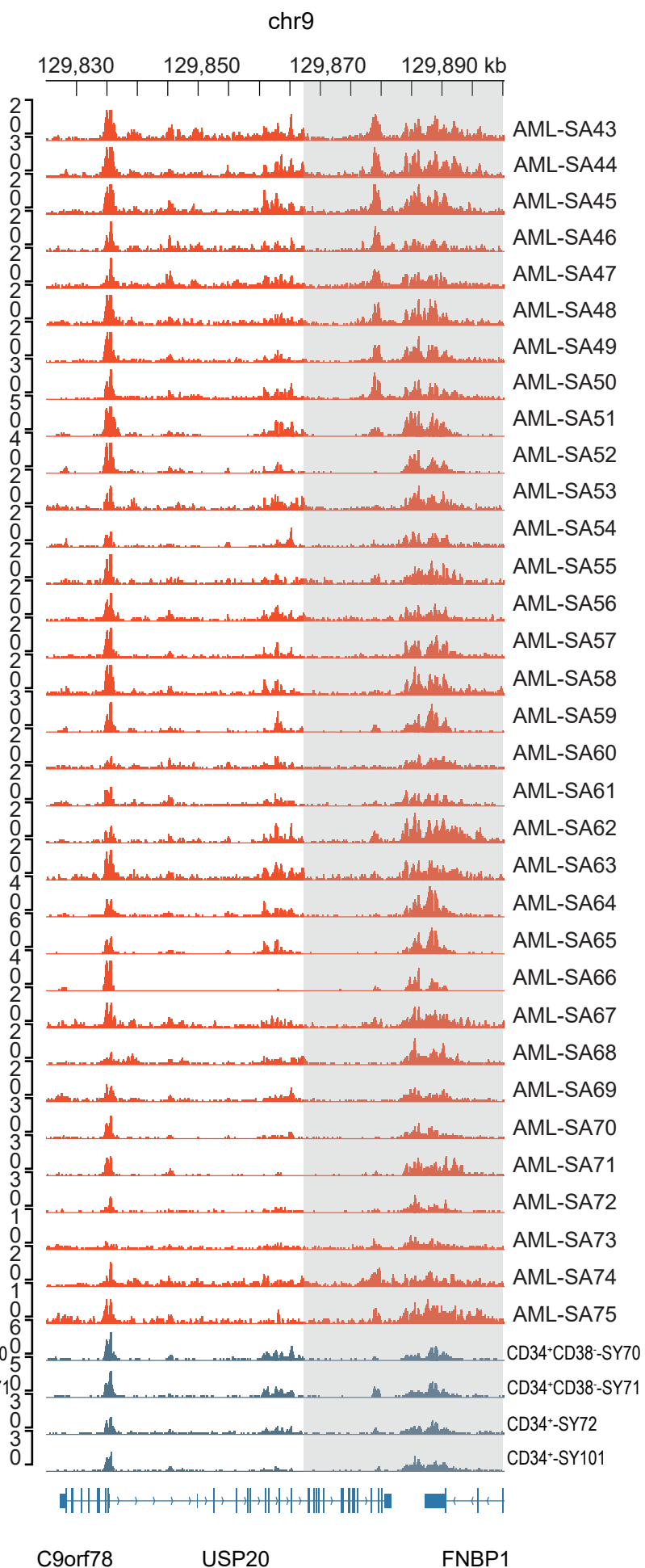

Supplement: Supplementary file 2 — Supplementary figures. [file ijbsv22p2665s2.zip › 附图/Supplementary Figure2.pdf]

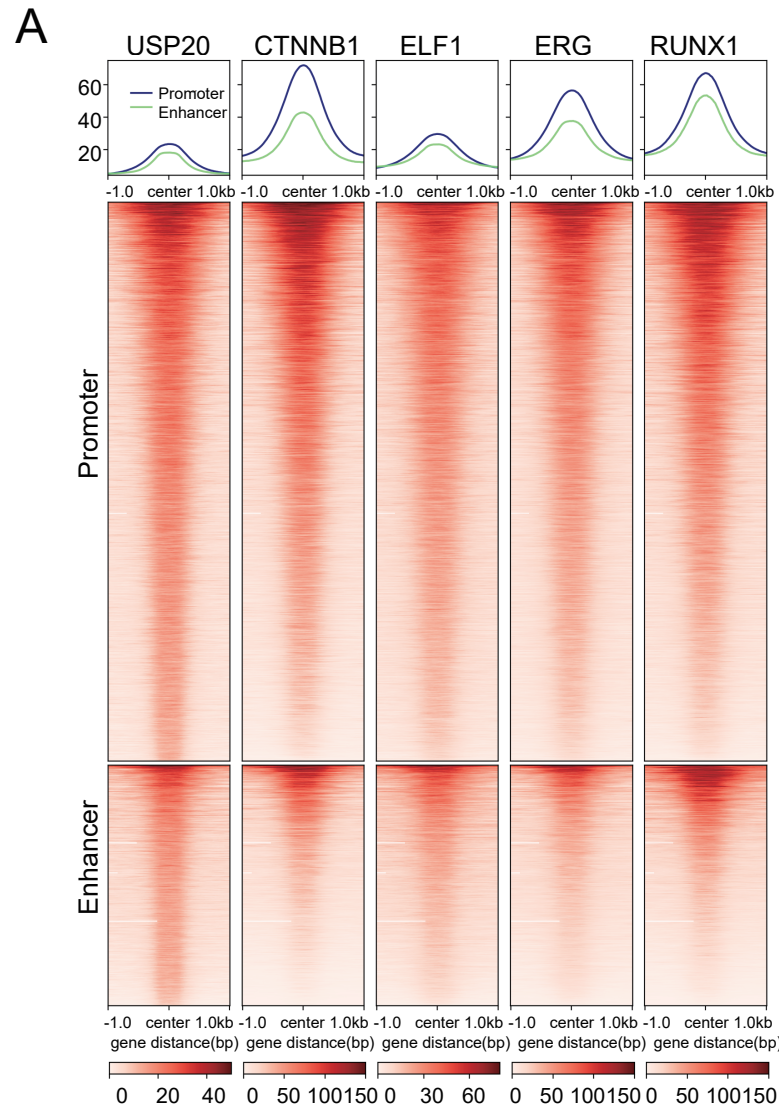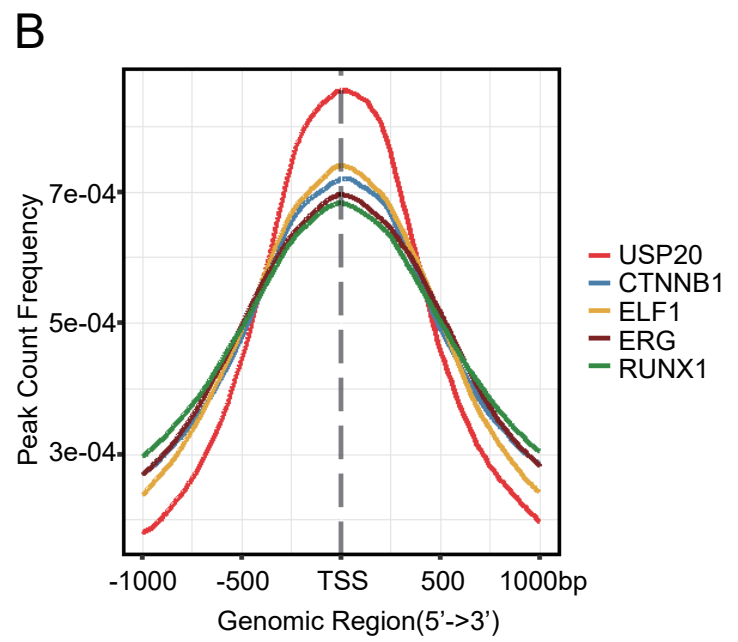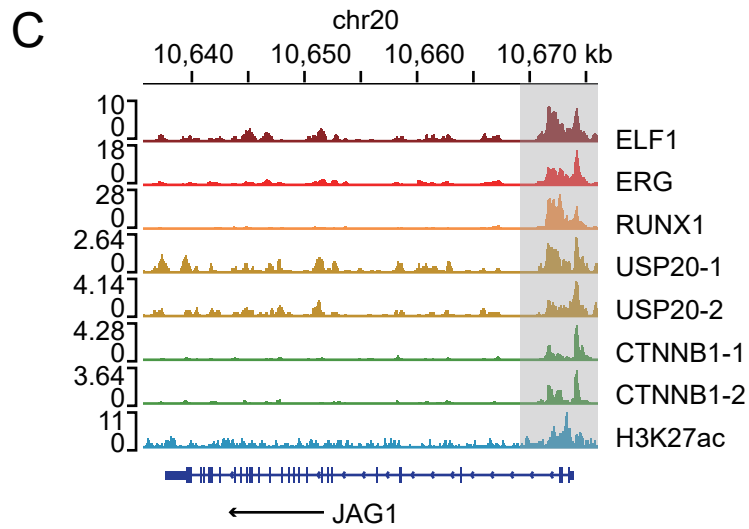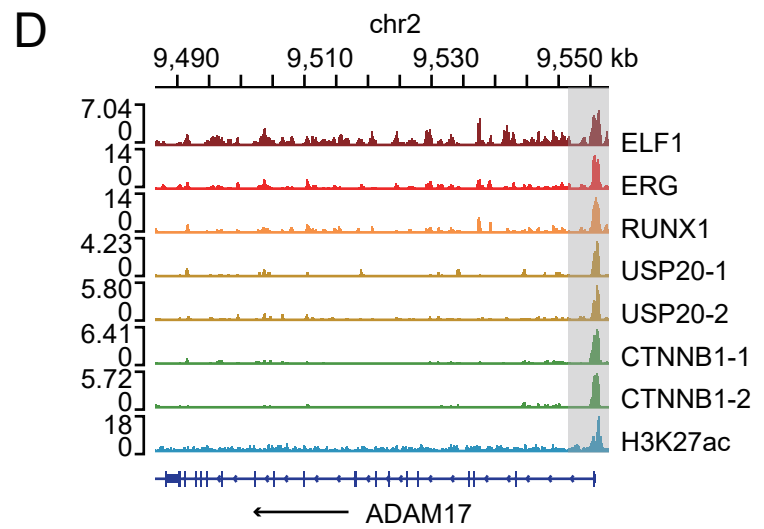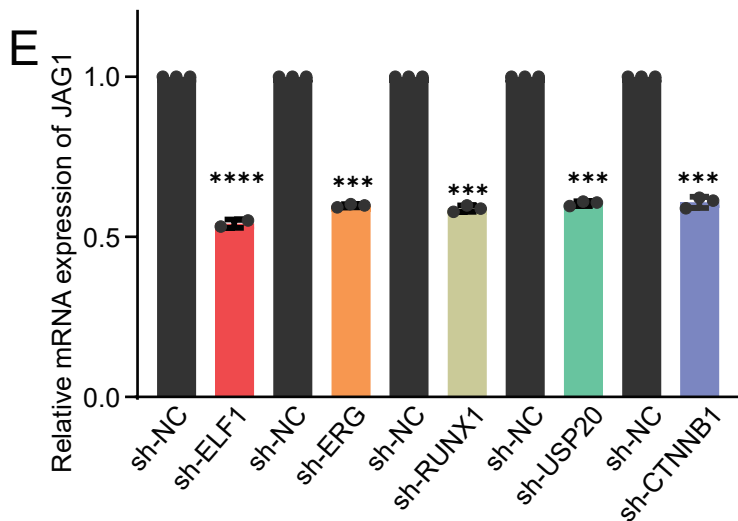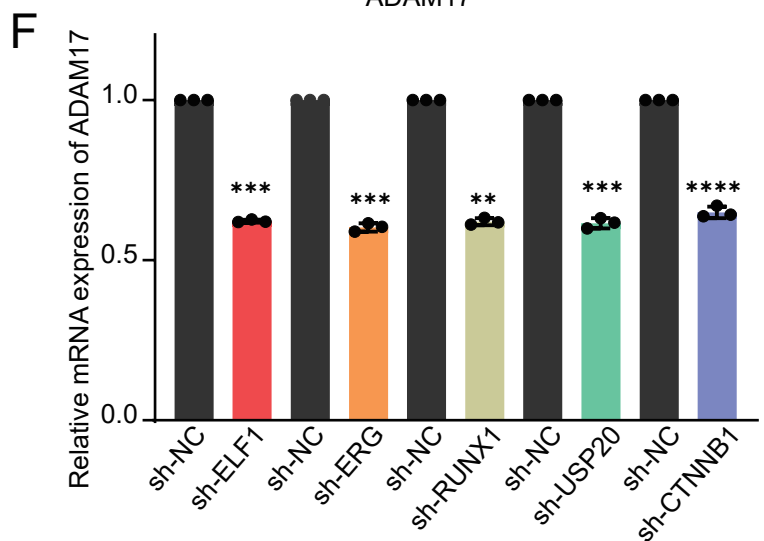

Supplement: Supplementary file 2 — Supplementary figures. [file ijbsv22p2665s2.zip › 附图/Supplementary Figure21.pdf]

A

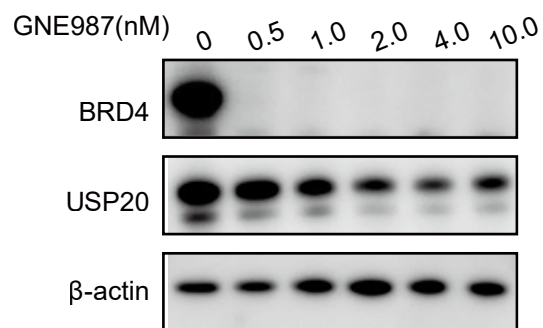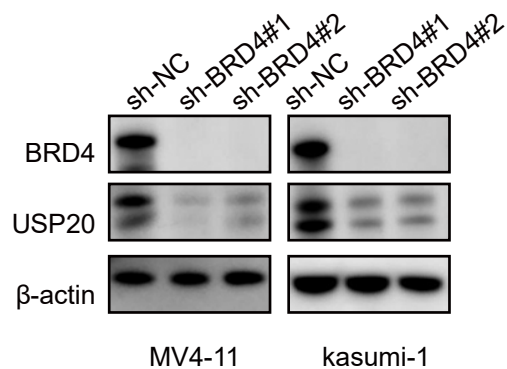

B

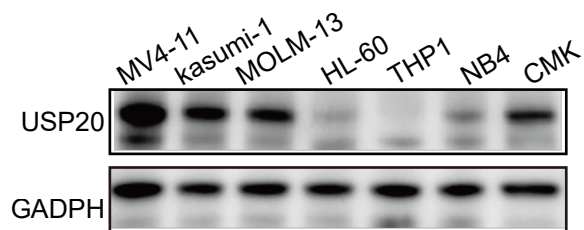

C

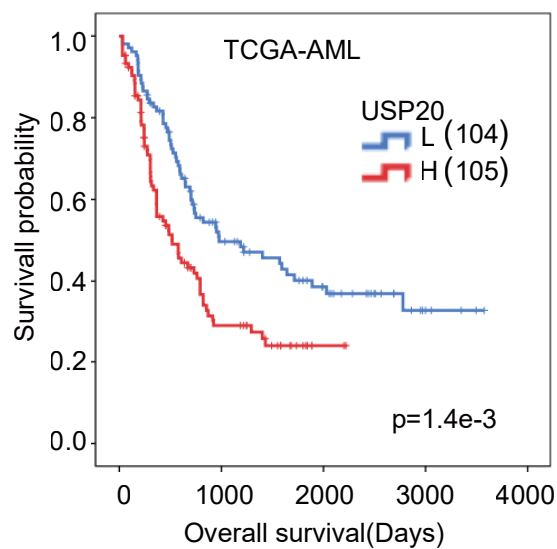

Supplement: Supplementary file 2 — Supplementary figures. [file ijbsv22p2665s2.zip › 附图/Supplementary Figure3.pdf]

MV4-11-CAS9

Sg-NC

E3-sgRNA2

E3-sgRNA4+5

E3-sgRNA6+7

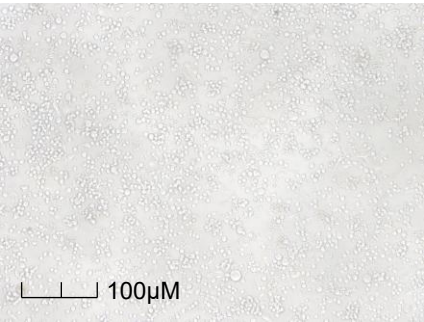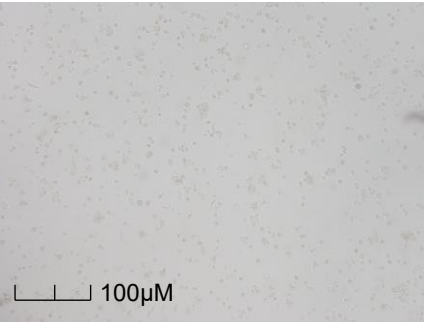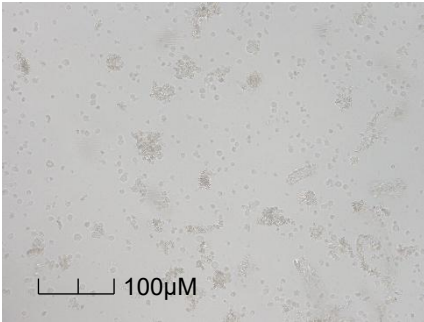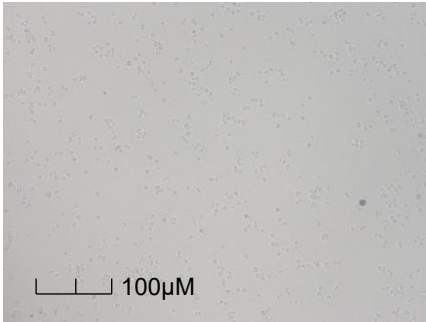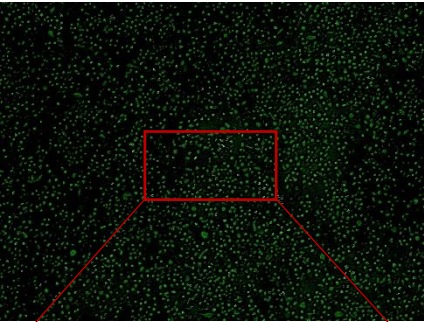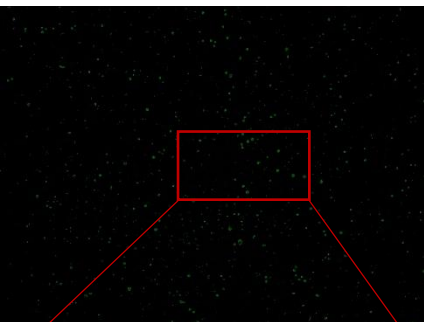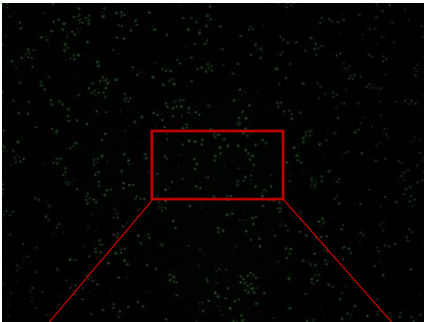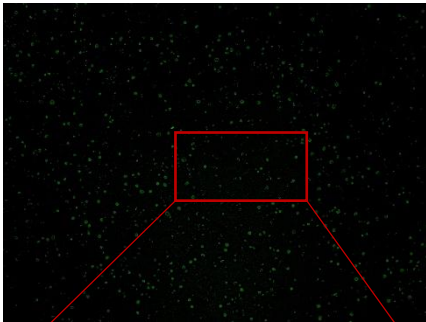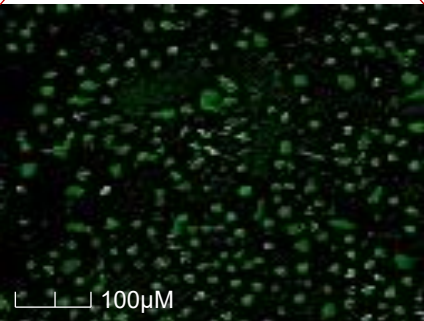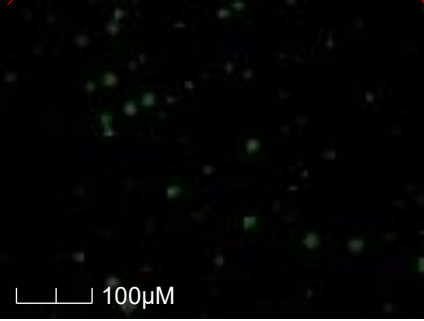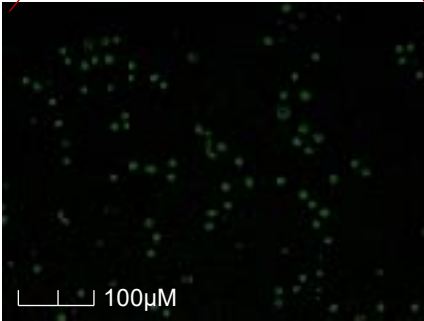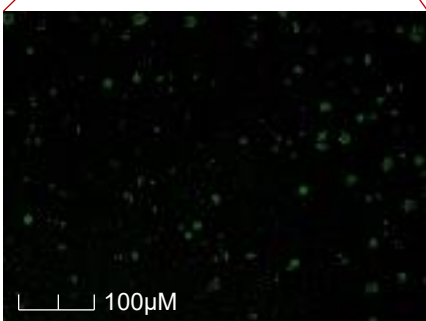

Kasumi-1-CAS9

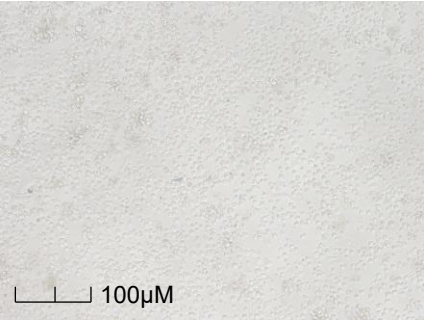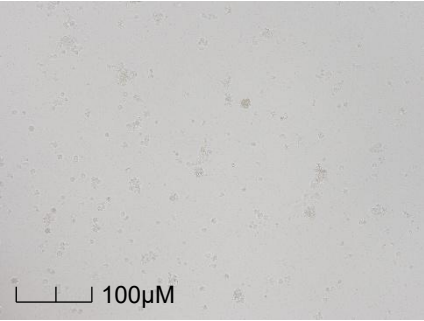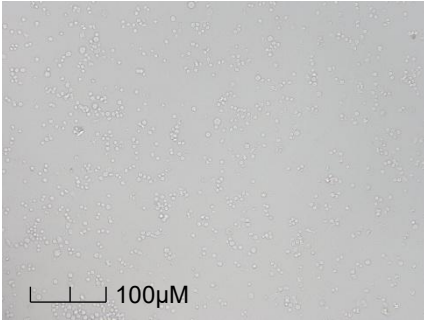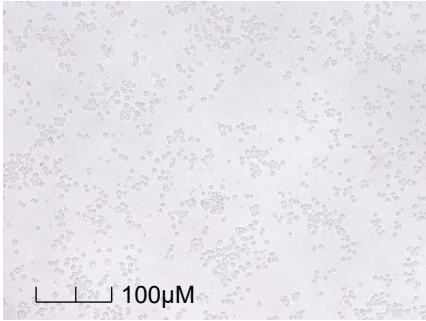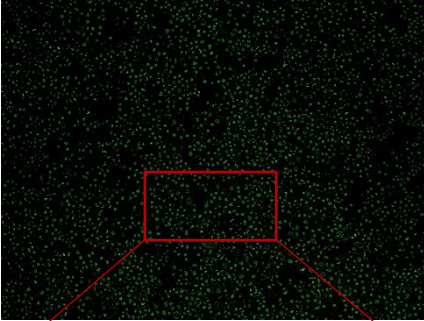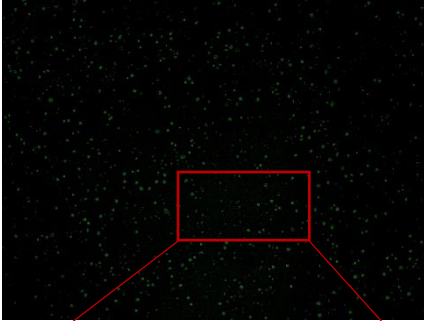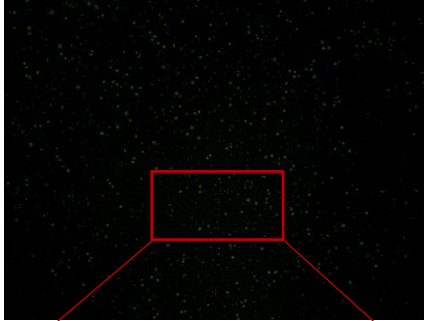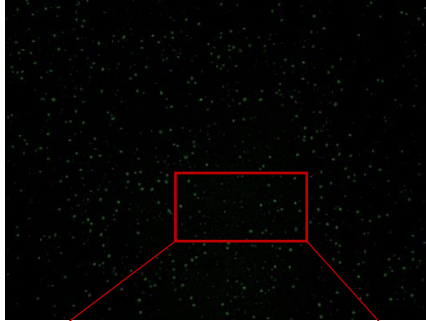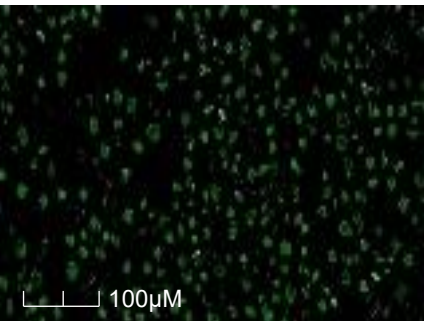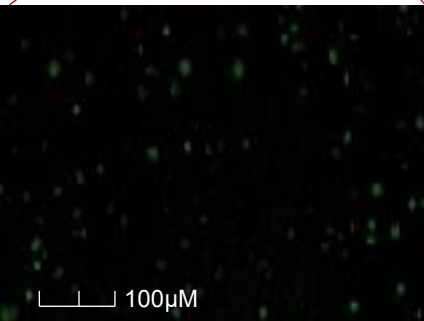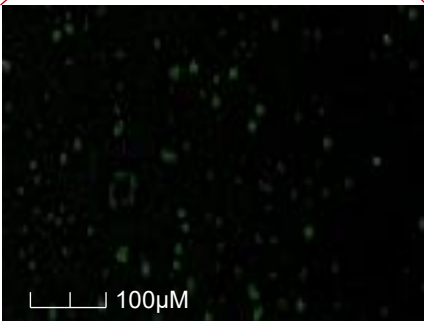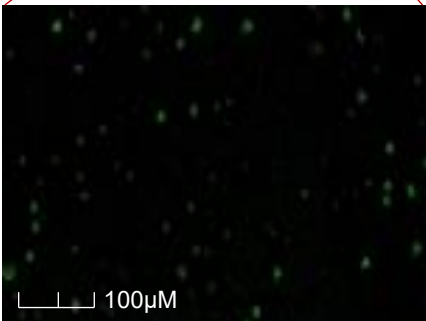

Supplement: Supplementary file 2 — Supplementary figures. [file ijbsv22p2665s2.zip › 附图/Supplementary Figure4.pdf]

A

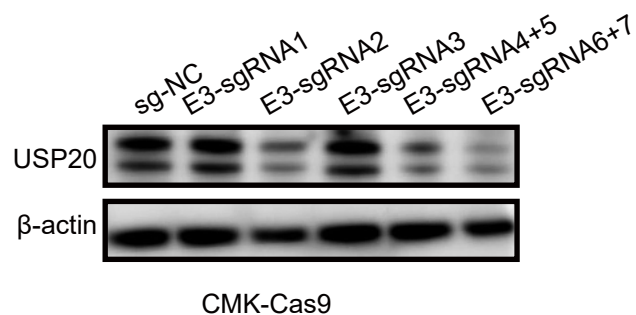

B

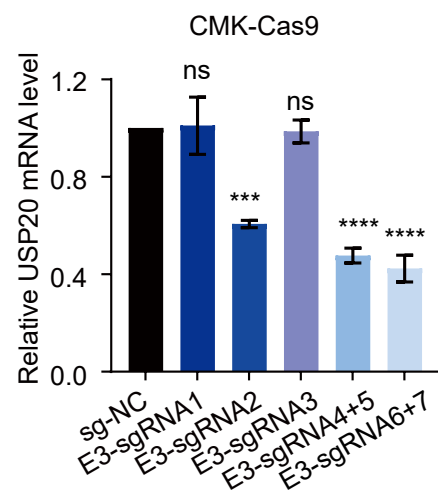

C

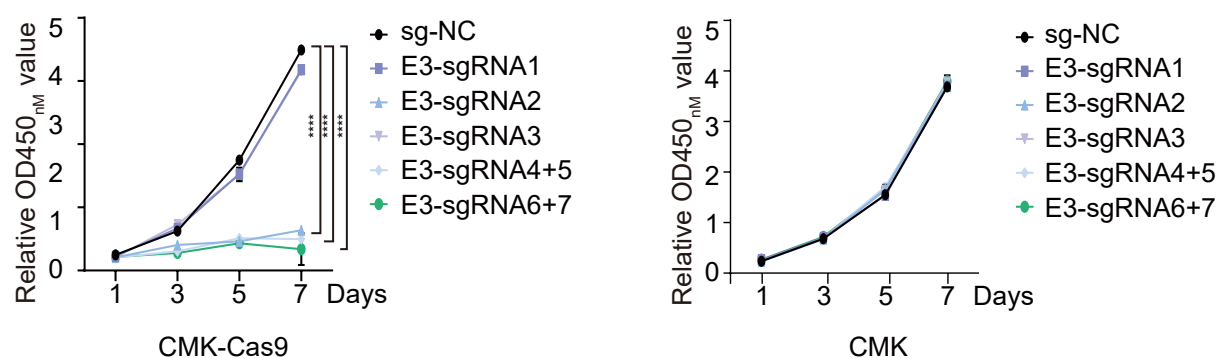

D

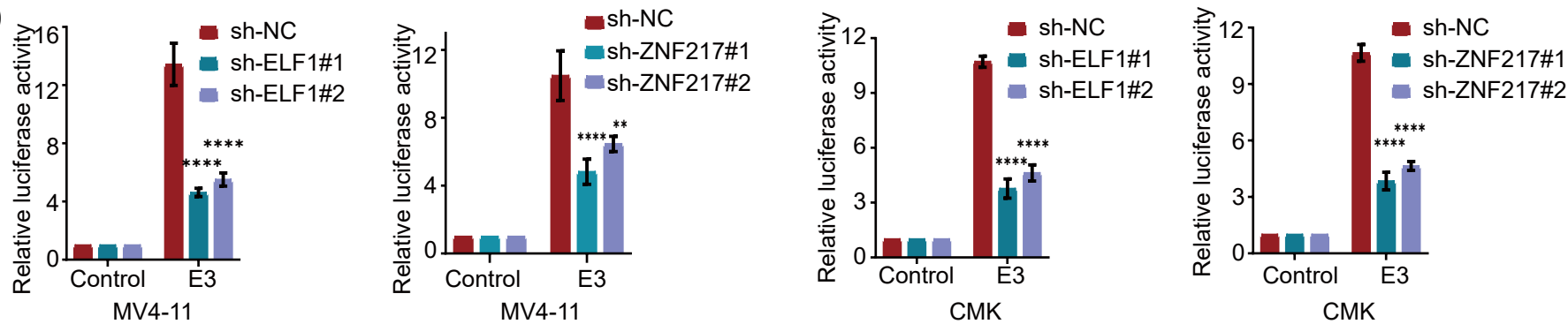

E

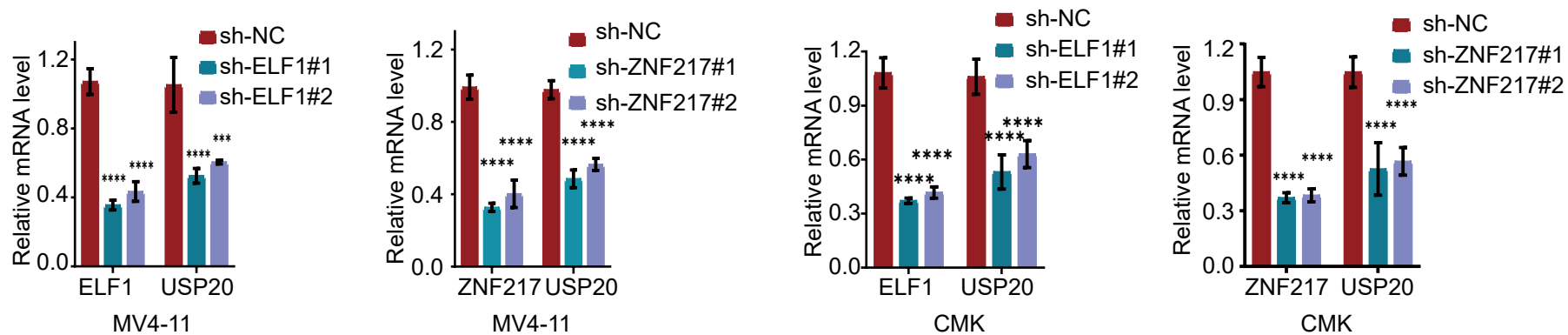

Supplement: Supplementary file 2 — Supplementary figures. [file ijbsv22p2665s2.zip › 附图/Supplementary Figure5.pdf]

MV4-11

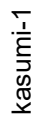

CMK

MV4-11

kasumi-1

CMK

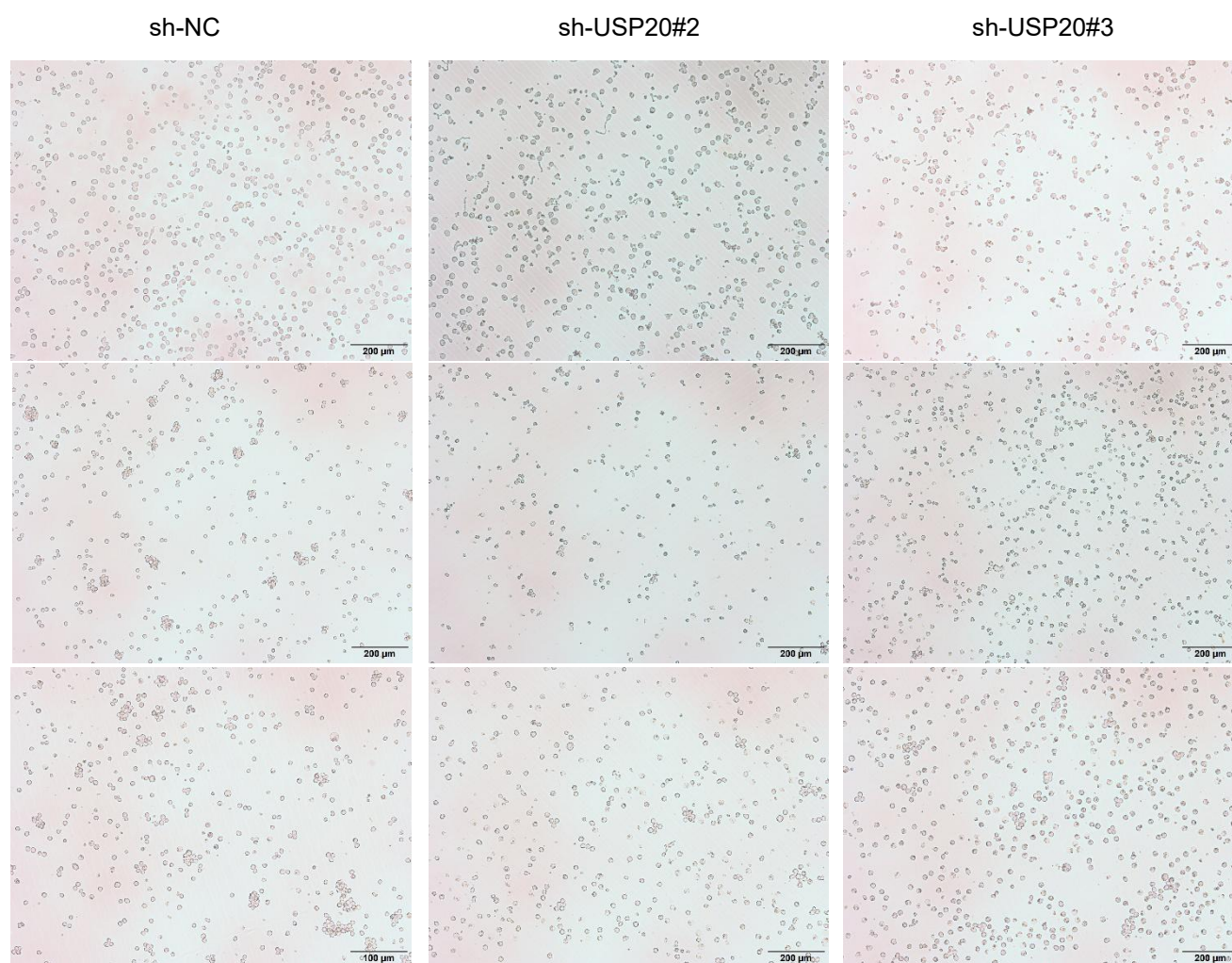

Supplement: Supplementary file 2 — Supplementary figures. [file ijbsv22p2665s2.zip › 附图/Supplementary Figure6.pdf]

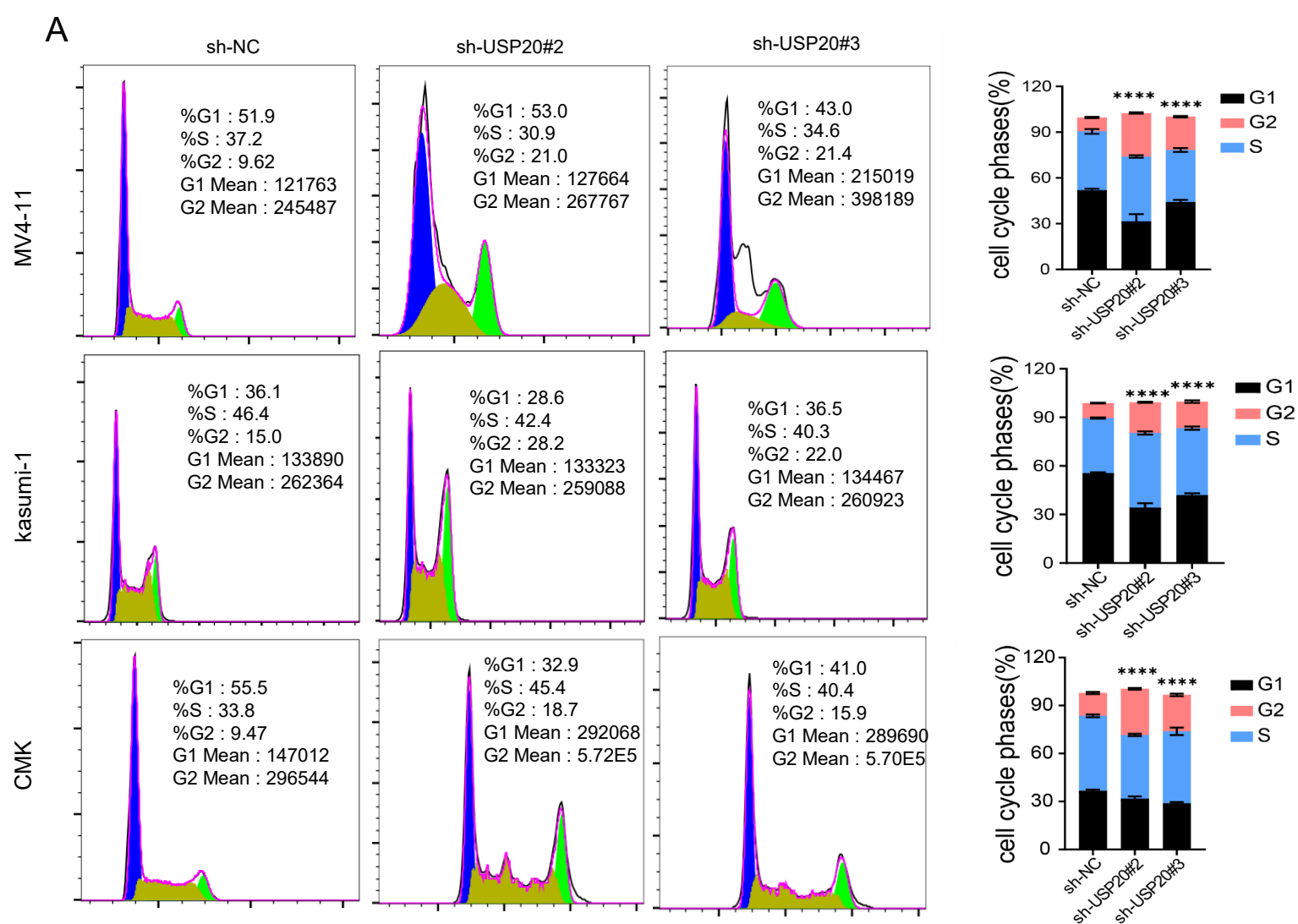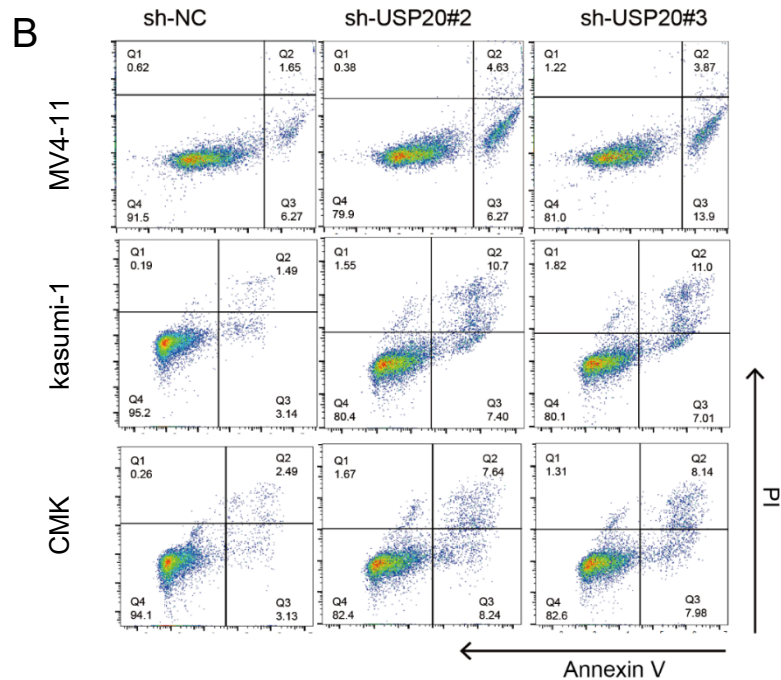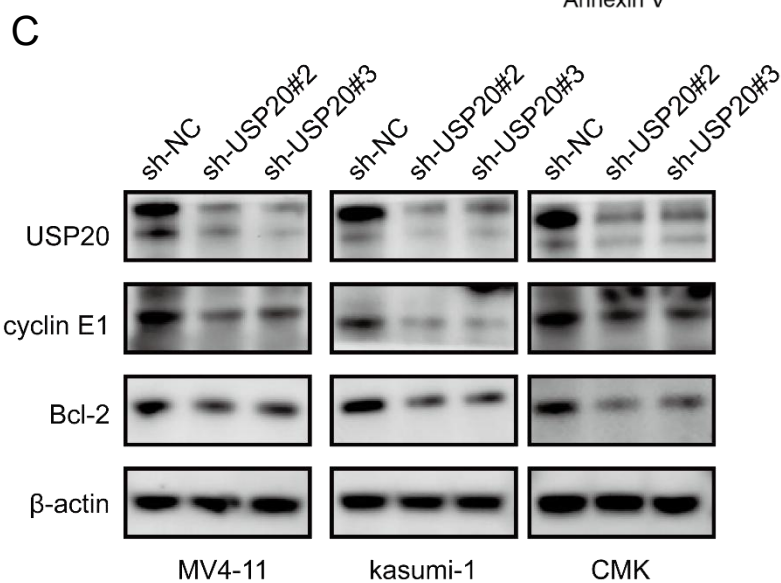

Supplement: Supplementary file 2 — Supplementary figures. [file ijbsv22p2665s2.zip › 附图/Supplementary Figure7.pdf]

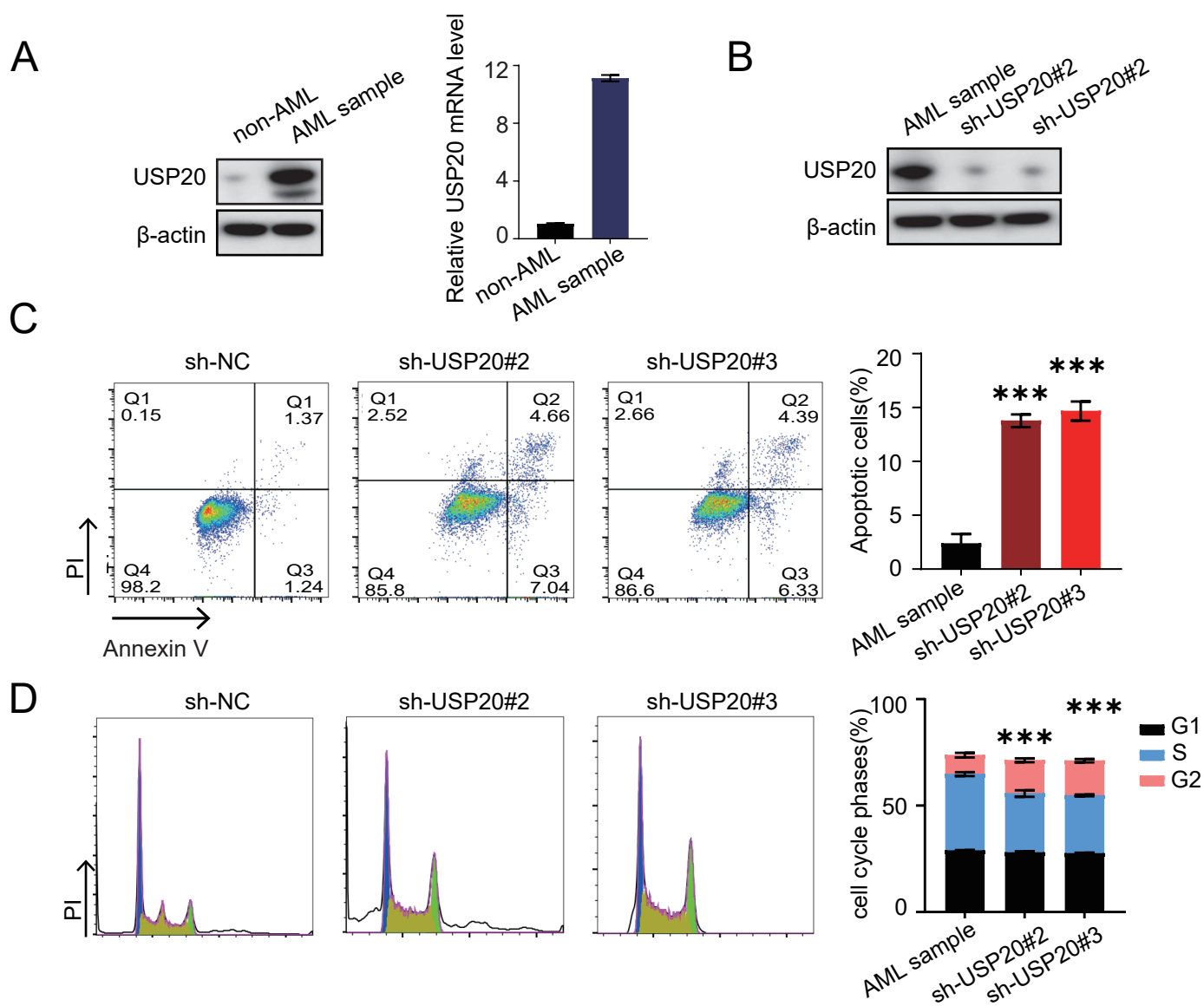

Supplement: Supplementary file 2 — Supplementary figures. [file ijbsv22p2665s2.zip › 附图/Supplementary Figure8.pdf]

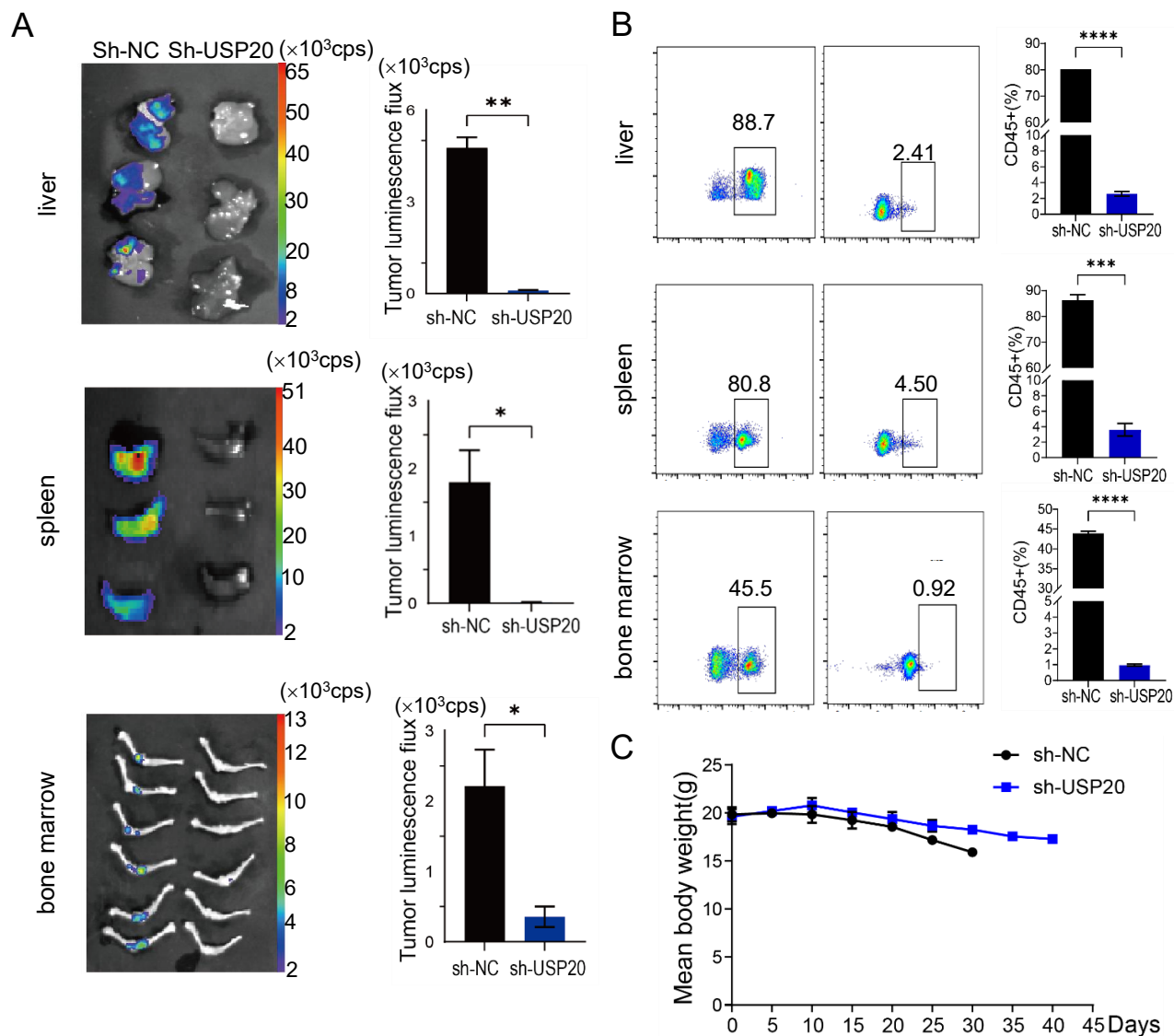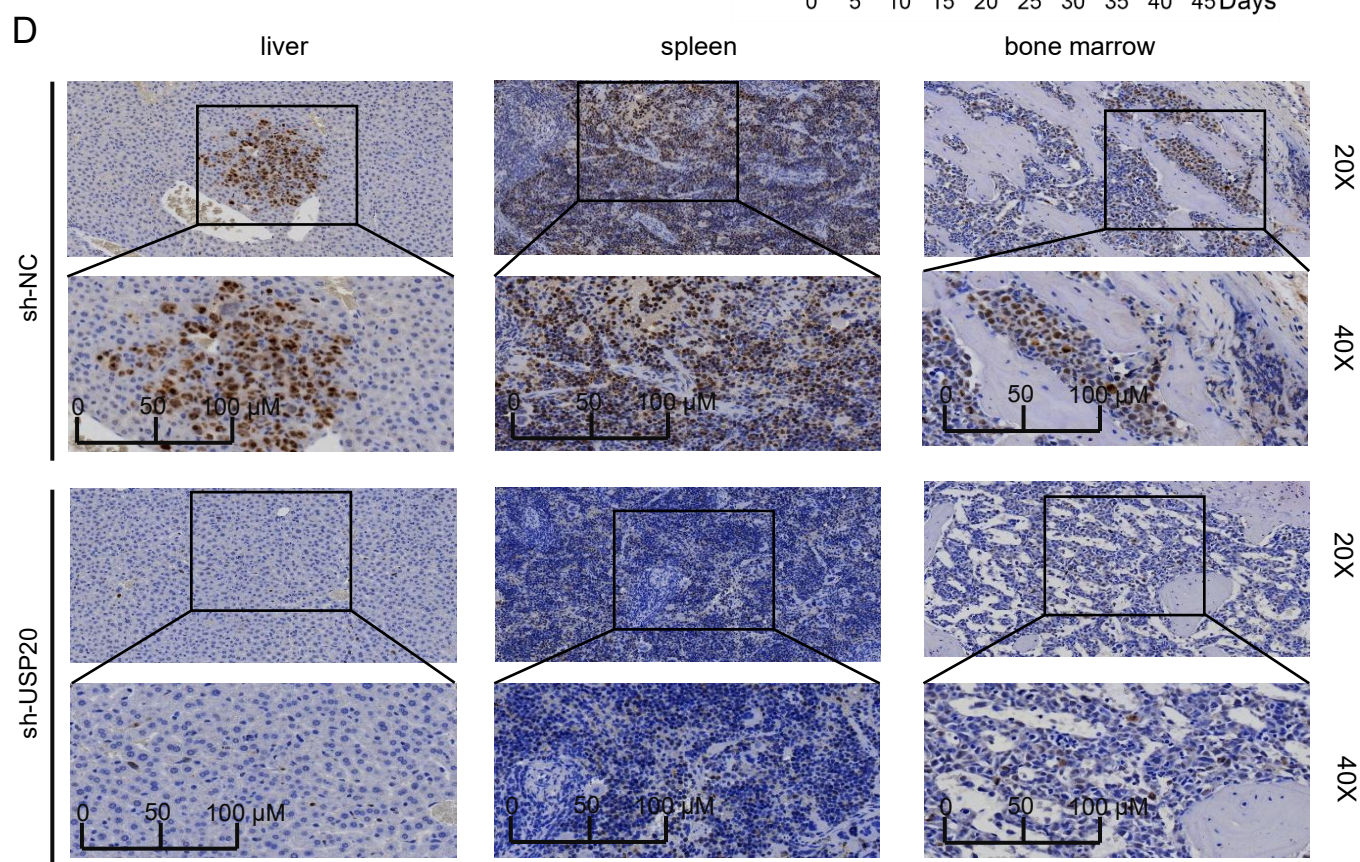

Supplement: Supplementary file 2 — Supplementary figures. [file ijbsv22p2665s2.zip › 附图/Supplementary Figure9.pdf]
